# Raptin, a sleep-induced hypothalamic hormone, suppresses appetite and obesity

**DOI:** 10.1038/s41422-025-01078-8

**Published:** 2025-01-29

**Authors:** Ling-Qi Xie, Biao Hu, Ren-Bin Lu, Ya-Lun Cheng, Xin Chen, Jie Wen, Yao Xiao, Yu-Ze An, Ning Peng, Yu Dai, Genqing Xie, Qi Guo, Hui Peng, Xiang-Hang Luo

**Affiliations:** 1 Department of Endocrinology, Endocrinology Research Center, Xiangya Hospital of Central South University, Changsha, Hunan China; 2 Department of Endocrinology, The First People’s Hospital of Xiangtan City, Xiangtan, Hunan China; 3 National Clinical Research Center for Geriatric Disorders, Xiangya Hospital, Changsha, Hunan China; 4 Key Laboratory of Aging-related Bone and Joint Diseases Prevention and Treatment, Ministry of Education, Xiangya Hospital, Central South University, Changsha, Hunan China; 5 FuRong Laboratory, Changsha, Hunan China

**Keywords:** Cell biology, Molecular biology

## Abstract

Sleep deficiency is associated with obesity, but the mechanisms underlying this connection remain unclear. Here, we identify a sleep-inducible hypothalamic protein hormone in humans and mice that suppresses obesity. This hormone is cleaved from reticulocalbin-2 (RCN2), and we name it Raptin. Raptin release is timed by the circuit from vasopressin-expressing neurons in the suprachiasmatic nucleus to RCN2-positive neurons in the paraventricular nucleus. Raptin levels peak during sleep, which is blunted by sleep deficiency. Raptin binds to glutamate metabotropic receptor 3 (GRM3) in neurons of the hypothalamus and stomach to inhibit appetite and gastric emptying, respectively. Raptin-GRM3 signaling mediates anorexigenic effects via PI3K-AKT signaling. Of note, we verify the connections between deficiencies in the sleeping state, impaired Raptin release, and obesity in patients with sleep deficiency. Moreover, humans carrying an *RCN2* nonsense variant present with night eating syndrome and obesity. These data define a unique hormone that suppresses food intake and prevents obesity.

## Introduction

Sleep deficiency is prevalent in modern society and has become one of the major causes of metabolic diseases.^1–3^ Previous studies demonstrated that insufficient sleep increases energy intake, while its effects on energy expenditure are conflicting.^4–6^ Recent clinical research has shown that populations with sleep deprivation display a greater energy intake but with no difference in energy expenditure.^7^ Thus, the mechanisms by which sleep deficiency contributes to obesity deserve further study.

Sleep, a circadian behavior, is involved in body rhythms and hormonal homeostasis.^8,9^ The disruption of the circadian rhythm caused by sleep deficiency can disrupt the levels of appetite hormones, including ghrelin, leptin, orexins, PYY and GLP-1.^10–14^ The hypothalamus, as a critical brain region of hormone secretion, can be affected by disrupted sleep–wakefulness.^15^ The paraventricular nucleus (PVN) of the hypothalamus is a vital area of the brain that synthesizes and releases hormones. The hypothalamic suprachiasmatic nucleus (SCN), a circadian center, entrains rhythms into the PVN to time the daily release of hypothalamic hormones,^16,17^ thus playing critical roles in energy homeostasis.^18^ Therefore, identifying new hypothalamic hormones that are affected by circadian rhythms, including those of sleep, should provide key new insights into the treatment of obesity.

Here, we identified Raptin as a sleep-inducible hypothalamic hormone. Using several mouse models, we found that Raptin was produced in the PVN and regulated by AVP^+^ neurons in the SCN to exert anorexigenic effects by acting on the functional receptor glutamate metabotropic receptor 3 (GRM3) in the neurons of the PVN and stomach. We also collected and analyzed clinical data to show that patients with obesity and sleep deficiency had a negative correlation with Raptin levels while patients who underwent sleep restriction therapy (SRT) showed an increase in Raptin levels. Moreover, we found that patients with obesity carrying a heterozygous *RCN2* nonsense variant had lower Raptin levels during the sleep phase and exhibited evening hyperphagia. Therefore, to the best of our knowledge, Raptin represents a new rhythmic protein hormone that suppresses appetite, providing new insights into body weight control.

## Results

### Raptin is a sleep-induced hypothalamic hormone in mice and humans

To identify the mechanisms by which long-term sleep disturbances contribute to obesity, we utilized the previously reported sleep fragmentation (SF) mouse model that involves the disruption of continuous sleep from Zeitgeber (ZT) 0 to ZT18. Our data showed that a 2-momth SF intervention accelerated body weight gain and increased food intake (Supplementary information, Fig. S1a, b). However, we found no significant difference in energy expenditure, prompting us to focus on energy intake (Supplementary information, Fig. S1c, d).

**Fig. 1:**
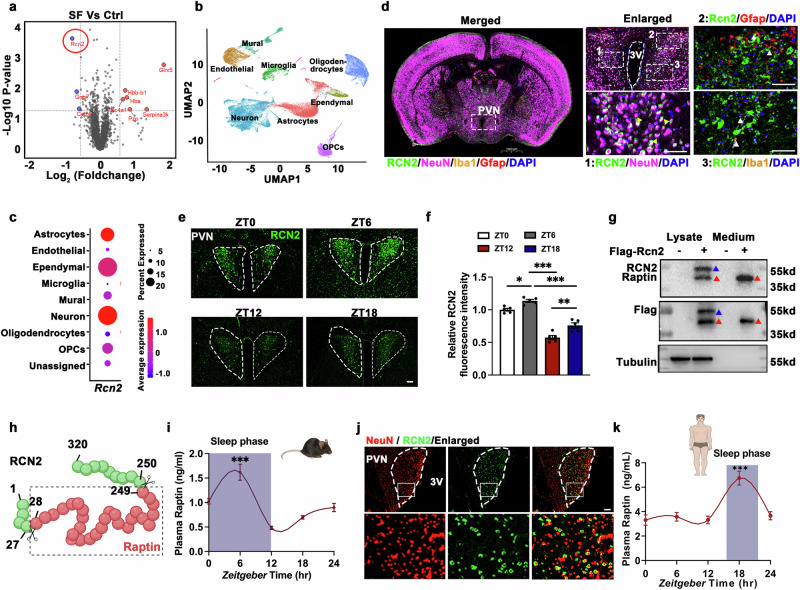
Raptin is a sleep-induced hypothalamic hormone in mice and humans. **a** Volcano plots of dysregulated factors ( ≥ 1.5-fold change) identified in the hypothalamus from sleep fragmentation mice (SF) compared to age-matched controls through proteomics and MS analysis (*n* = 3 per group). The red circle indicates Rcn2. **b** UMAP plot showing the clustering of cell types in the hypothalamus by analyzing multiple public scRNA-seq data from the GSE87544, GSE119960, GSE132355, GSE132730 and GSE148568 datasets. **c** Dot plots displaying *Rcn2* expression in different cell types of mouse hypothalamus from scRNA-seq data. **d** Representative image of RCN2 (green) expression in the neurons (NeuN, violet, yellow arrows indicate the colocalization), microglia (IBA1, orange, white arrows) and astrocytes (GFAP, red, white arrows) of mouse brain slices. Scale bars, 50 μm. **e**, **f** Representative images (**e**) and quantification of RCN2 (green, **f**) expression in the PVN of mice at different time points (ZT0, ZT6, ZT12, ZT18). Scale bar, 50 μm (*n* = 5 per group). **g** Representative western blot of RCN2 expression in cell lysates and the concentrated culture medium of hypothalamic GT1-7 neurons transfected with the Flag-*Rcn2* plasmid. The red triangle indicates Raptin (MW: 35–55 kD), and blue triangle indicates full-length RCN2 (MW: ~55 kD). **h** Schematic diagram illustrating the cleavage of RCN2 into Raptin. Raptin is spanning from 28 to 249 amino acids of full-length RCN2. **i** Plasma Raptin levels of 3-month male mice monitored at ZT0, ZT6, ZT12 and ZT18 (the purple area indicates the sleep phase: ZT0–ZT12). **j** Representative images of RCN2 (green) and NeuN (red) expression in the human hypothalamus section. Scale bar, 200 μm. **k** Plasma Raptin levels of humans monitored at ZT0, ZT6, ZT12 and ZT18 (purple area indicates sleep phase). Data are shown as the mean ± SEM. *P < 0.05, **P < 0.01, ***P < 0.001 by one-way ANOVA (**f**, **i**, **k**). See also Supplementary information, Figs. S1, S2.

The hypothalamus serves as a central hub for both the neuroendocrine system and the regulation of the sleep–wake cycle.^9,19^ Appetite-regulating hormones play a crucial role in linking energy intake to hypothalamic activity.^11,20^ To identify whether there were additional unknown factors contributing to SF-induced metabolic dysfunction, we performed mass spectrometry (MS) analysis of the hypothalami from control and SF mice. We found 3 downregulated and 6 upregulated factors in the hypothalami from the SF mice compared to the control mice (Fig. 1a). Among the 9 changed proteins, only three proteins (SERPINA3K, PZP, RCN2) were secreted proteins.^21–23^ SERPINA3K and PZP have both been reported to be mainly expressed in the liver, however, RCN2 was reported to be mostly expressed in the hypothalamus based on our analysis of a publicly available database. RCN2 is an endoplasmic reticulum-localized calcium-binding protein with reported roles in bone formation and cancer,^22,24^ which we have studied in the past.^22^ Thus, we selected RCN2 for our further study here.

By western blot analysis, we confirmed higher RCN2 abundance in the hypothalamus compared to a range of other tissues (Supplementary information, Fig. S1e, f). To further elucidate its cellular origin in the hypothalamus, we integrated data from 5 publicly available hypothalamus single-cell RNA-sequencing (scRNA-seq) datasets based on mice and found that *Rcn2* was predominantly expressed in neurons (Fig. 1b, c). By immunofluorescence-based microscopy, we found that RCN2 was highly expressed in the PVN rather than other nuclei of the hypothalamus or thalamus in mice (Supplementary information, Fig. S1g, h). Moreover, RCN2 was predominantly expressed in neurons (NeuN^+^), rather than microglia (IBA1^+^) or astrocytes (GFAP^+^), in the PVN (Fig. 1d).

We further confirmed a significantly higher RCN2 expression in the PVN during the sleep phase (ZT0 and ZT6) in contrast to the active phase (ZT12 and ZT18) (Fig. 1e, f). As the PVN is a key region for the release of hypothalamic hormones, we hypothesized that RCN2 might be a novel hypothalamic hormone. To test this hypothesis, we constructed a plasmid harboring full-length *Rcn2* fused with a Flag-tag and transfected the construct into hypothalamic GT1-7 neurons. Subsequently, we identified a shorter fragment in the concentrated cell culture medium (Fig. 1g), suggesting that it might be cleaved from full-length RCN2 and then secreted. Then, we identified the amino acid sequence of this secreted protein by MS analysis and confirmed the conservation of RCN2 spaning amino acids 28–249. We named this cleaved fragment of RCN2 as “Raptin” (Fig. 1h; Supplementary information, Fig. S2a).

We next measured the daily plasma rhythms of Raptin in mice and found that its peak secretion occurred during sleep period (ZT0–ZT12) (Fig. 1i). In addition, our investigation revealed robust expression of RCN2 in PVN neurons in human hypothalamus sections and the peak of plasma Raptin aligning with the sleep period, notably at ZT18 (1 AM) (Fig. 1j, k).

To identify the enzyme that cleaves RCN2 between R249 and L250, we searched for the candidate endopeptidases that were predicted to cleave the NDGR-LDPQ sequence and identified kallikrein-related peptidase (including KLK15, 14, 1, 4) as the potential proteases responsible for RCN2 cleavage. We found that the secretion of Raptin was abrogated by aprotinin, a non-specific proprotein convertase inhibitor that acts on serine proteases, including KLKs^25^ (Supplementary information, Fig. S2b, c). Overexpression of KLK1 or KLK4 dramatically enhanced the level of Raptin (Supplementary information, Fig. S2d, e). Both KLK4 and KLK1 were detected in the hypothalamus and hypothalamic neurons (Supplementary information, Fig. S2f–i), and they were co-stained with RCN2 (Supplementary information, Fig. S2j, k). Of note, neither KLK1 nor KLK4 expression displayed a rhythmic change in the PVN of mice (Supplementary information, Fig. S2l–o), suggesting that the circadian variation of Raptin secretion levels is likely caused by a change in precursor expression rather than by its circadian cleavage.

Collectively, our data suggest that Raptin might be a sleep-inducible hormone both in mice and humans.

### The circadian rhythm of Raptin release is controlled by SCN^AVP^ neurons

To mark RCN2-positive neurons in the PVN, we performed stereotaxic microinjections of recombinant adeno-associated virus (AAV) vectors carrying a 2.3 kb region of the *Rcn2* promoter and an *EGFP* reporter gene (AAVs-Rcn2-Cre-EGFP) into the PVN of mice. Three weeks later, by in situ hybridization (ISH) using a *Rcn2* RNA probe, we found robust co-localization of EGFP and *Rcn2* mRNA, which suggested the effectiveness of the virus in labeling RCN2-positive neurons in the PVN (Supplementary information, Fig. S3a, b).

**Fig. 2:**
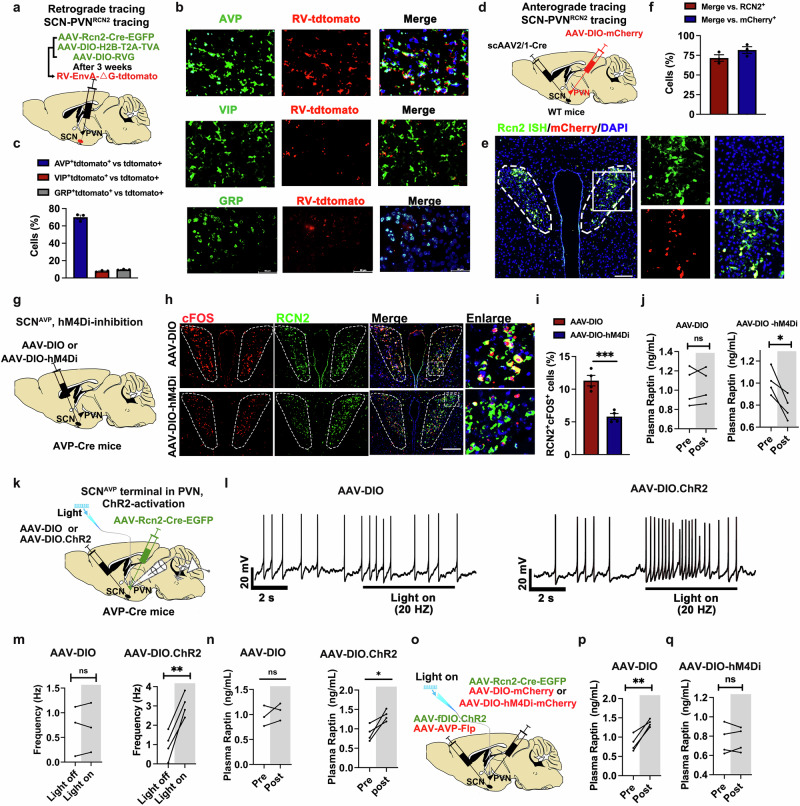
The circadian rhythm of Raptin release is controlled by SCN^AVP^ neurons. **a** Schematic diagram of the retrograde tracing system in SCN-innervating PVN^RCN2^. AAV-Rcn2-Cre-EGFP, AAV-DIO-H2B-T2A-TVA and AAV-DIO-RVG were mixed and then bilaterally injected into PVN. Three weeks later, rabies virus (RV)-EnvA-tdTomato (red) were bilaterally injected into PVN. **b**, **c** Representative images (**b**) and quantification (**c**) of colocalization with RV-tdTomato (red) by immunofluorescence staining of AVP^+^ neurons (top line, green), VIP^+^ neurons (middle line, green) and GRP^+^ neurons (bottom line, green) in SCN. Scale bars, 50 μm (*n* = 3 per group). **d** Schematic diagram of the anterograde tracing system in SCN-innervating PVN. The WT mice were bilaterally injected with scAAV2/1-hSyn-Cre into SCN and AAV-DIO-mCherry into PVN. **e**, **f** Representative images (**e**) and quantification (**f**) of colocalization with mCherry (red) by fluorescence ISH of *Rcn2* mRNA (green) in PVN neurons. Scale bar, 50 μm (*n* = 3). **g** Schematic diagram of chemogenetic inhibition of SCN^AVP^ neuron of *Avp*-Cre mice. AAV-DIO or AAV-DIO-hM4Di was injected into SCN of *Avp*-Cre mice. Mice were intraperitoneally injected with CNO at a dose of 2 mg/kg body weight. **h**, **i** Representative images (**h**) and quantification (**i**) of co-staining of c-Fos (red) and RCN2 (green) in PVN after CNO injection for 60 min to induce SCN^AVP^ neuron inhibition in *Avp*-Cre mice. Scale bar, 50 μm (*n* = 4 per group). **j** Plasma Raptin levels of mice injected with AAV-DIO or AAV-DIO-hM4Di before and after intraperitoneal injection of CNO for 1 h (*n* = 4 per group). **k** Schematic diagram illustrating the optogenetic activation of SCN^AVP^ neuron terminal in PVN, followed by the detection of PVN^RCN2^ neuron activity. AAV-DIO or AAV-DIO.ChR2 was bilaterally injected into SCN, whereas AAV-Rcn2-Cre-EGFP was injected into the PVN of *Avp*-Cre mice. The fiber was implanted into the PVN to stimulate SCN^AVP^ neuronal terminal. **l**, **m** Representative traces (**l**) and action potential frequency (**m**) of PVN^RCN2^ neurons in brain slice before and during optogenetic activation of SCN^AVP^ neurons (*n* = 3 ~ 4 per group). **n** The plasma Raptin levels before and after optogenetic activation of SCN^AVP^ neurons in vivo (*n* = 3 ~ 4 per group). **o** Schematic diagram illustrating the experimental workflow for SCN^AVP^ neuron activation and PVN^RCN2^ neuron inhibition. AAV-DIO-mCherry or AAV-DIO-hM4Di-mCherry with AAV-Rcn2-Cre-EGFP were injected into PVN for chemogenetic manipulation of PVN^RCN2^ neuron. CNO was intraperitoneally injected into mice at a dose of 2 mg/kg body weight. AAV-fDIO.ChR2 with AAV-Avp-Flp were injected into SCN for optogenetic activation of SCN^AVP^ neurons. The fiber was implanted into the PVN to stimulate SCN^AVP^ neuronal terminal in PVN. **p,** **q** The plasma Raptin levels in mice injected with AAV-DIO (**p**) or AAV-DIO-hM4Di (**q**) before and after CNO treatment (*n* = 4 per group). Data are shown as the mean ± SEM. *P < 0.05, **P < 0.01 by a two-tailed paired Student’s *t*-test (**j**, **m**, **n**, **p**, **q**) or by a two-tailed unpaired Student’s *t*-test (**i**). See also Supplementary information, Figs. S3, S4.

The SCN, serving as the central circadian pacemaker, exhibits a robust innervation to the PVN, orchestrating the circadian rhythm of hormone secretion from the PVN.^16^ The SCN mainly harbors three neuronal subtypes, including vasopressin (AVP)-, vasoactive intestinal polypeptide (VIP)- and gastrin releasing peptide (GRP)-expressing neurons.^26^ *Trans*-synaptic retrograde tracing was conducted by injecting a modified rabies virus (RV-EnvA-ΔG-tdTomato), helper viruses (AAV-DIO-RVG and AAV-DIO-H2B-T2A-TVA) and PVN^RCN2^ labeling virus (AAV-Rcn2-Cre-EGFP) into the PVN of wild-type (WT) mice, which enabled labeling of input neurons that synapse onto PVN^RCN2^ neurons (Fig. 2a). Immunofluorescence staining of the SCN showed that AVP neurons were more strongly co-stained with tdTomato than the other two neuronal subtype (VIP and GRP) neurons, suggesting extensive inputs from SCN-AVP (SCN^AVP^) neurons to PVN-RCN2 (PVN^RCN2^) neurons (Fig. 2b, c). Moreover, we performed anterograde tracing by injecting the scAAV2/1-hSyn-Cre into the SCN and AAV-DIO-mCherry into the PVN of WT mice (Fig. 2d). Quantitative analysis revealed that ~72% of RCN2^+^ neurons in the PVN were connected to SCN^AVP^ neurons and ~82% of mCherry-positive neurons in the PVN were RCN2^+^ neurons (Fig. 2e, f).

To further determine the role of SCN^AVP^ neurons in regulating PVN^RCN2^ neuron activity, we injected AAV-EF1α-DIO-GCaMp6s-EGFP and AAV-Rcn2-Cre into the PVN, while concurrently injecting control (AAV-fDIO) or excitatory (AAV-fDIO-hM3Dq) or inhibitory (AAV-fDIO-hM4Di) chemogenetic virus with AAV-AVP-Flp (for selective manipulation of AVP neurons) into the SCN (Supplementary information, Fig. S3c). The success of localized injection and the expression of the virus were confirmed by ISH and immunofluorescence staining (Supplementary information, Fig. S3d–f). The calcium activity was elevated in PVN^RCN2^ neurons upon clozapine-N-oxide (CNO)-induced SCN^AVP^ neuron activation, whereas it was decreased upon SCN^AVP^ neuron inhibition (Supplementary information, Fig. S3g, h). These findings demonstrate that SCN^AVP^ neurons can control the activity of PVN^RCN2^ neurons.

To ascertain the involvement of SCN^AVP^ neurons in regulating Raptin release in the PVN, we conducted chemogenetic manipulations by injecting control (AAV-DIO) or inhibitory (AAV-DIO-hM4Di) chemogenetic viruses into the SCN of *Avp*-Cre mice (Fig. 2g). Compared to the control group, the hM4Di-inhibition group showed a lower activity of SCN^AVP^ neurons after intraperitoneal injection of 2 mg/kg CNO, as indicated by the lower colocalization of c-Fos with AVP in the SCN (Supplementary information, Fig. S3i, j). Consistent with these findings, CNO-induced inhibition of SCN^AVP^ neurons resulted in inhibition of PVN^RCN2^ neurons (Fig. 2h, i). Moreover, we found that SCN^AVP^ neuron inhibition resulted in less Raptin release (Fig. 2j) and greater food intake (Supplementary information, Fig. S3k).

We also investigated the role of SCN^AVP^ neurons in regulating PVN^RCN2^ neuron activity using optogenetic technology. AAV-Rcn2-Cre-EGFP was injected into the PVN while the Cre-dependent ChR2-expressing virus (AAV-DIO-ChR2) or control virus (AAV-DIO) was injected into the SCN of *Avp*-Cre mice, and an optic fiber was implanted in the PVN to stimulate SCN^AVP^ neuronal terminals (Fig. 2k). Optogenetic activation of SCN^AVP^ neuron terminals at the PVN resulted in the activation of PVN^RCN2^ neurons, as reflected by enhanced firing rate of PVN^RCN2^ neurons (Fig. 2l, m). In line with this, the plasma Raptin levels were augmented upon optogenetic activation of SCN^AVP^ neuronal terminals (Fig. 2n). The optogenetic activation of SCN^AVP^ neuronal terminals also effectively lowered the amount of 1-h food intake (Supplementary information, Fig. S4a).

To further confirm that PVN^RCN2^ neurons affect plasma Raptin levels, we investigated whether the optogenetic stimulation-induced SCN^AVP^ neuron activation still affected Raptin release in the PVN-specific *Rcn2* deletion mice. Thus, we crossed *Rcn2*-floxed mice (*Rcn2*^*flox*/*flox*^ mice) with *Sim1*-Cre mice and confirmed the effective knockout of *Rcn2* in the PVN (Supplementary information, Fig. S4b–e). Next, we injected AAV-Avp-Cre-mCherry (for selective manipulation of AVP neurons) and AAV-DIO-ChR2 into the SCN of PVN-specific *Rcn2* deletion mice (Supplementary information, Fig. S4f). We then confirmed the success of the localized injection and expression of the virus (Supplementary information, Fig. S4g, h). As expected, we found that the activation of SCN^AVP^ neuron terminals in the PVN failed to increase plasma Raptin levels in PVN-specific *Rcn2* deletion mice (Supplementary information, Fig. S4i). To further confirm the necessity of the PVN^RCN2^ neurons in this pathway, we firstly evaluated the effect of PVN^RCN2^ neurons on Raptin release via chemogenetic manipulation (Supplementary information, Fig. S4j). CNO-induced activation of PVN^RCN2^ neurons resulted in greater levels of plasma Raptin, while PVN^RCN2^ neuron inhibition lowered them (Supplementary information, Fig. S4k). Next, we manipulated PVN^RCN2^ neurons by a chemogenetic approach while activating SCN^AVP^ neuronal terminals via optogenetic stimulation (Fig. 2o). We found an increase in the levels of plasma Raptin when only SCN^AVP^ neurons were activated (Fig. 2p). However, the increase of plasma Raptin levels was abolished upon SCN^AVP^ neuron activation along with PVN^RCN2^ neuron inhibition (Fig. 2q).

Together, our results reveal that Raptin release is controlled by a circuit from the SCN^AVP^ neurons to the PVN^RCN2^ neurons.

### Hypothalamus-derived Raptin suppresses food intake and prevents obesity

The results above identified Raptin as a candidate sleep-induced hypothalamic hormone. Indeed, upon SF, there was less RCN2 expression in the PVN neurons and significantly lower plasma Raptin levels in mice compared to control male mice at ZT6 (Supplementary information, Fig. S5a–c). Considering the significant effect of light on the activity of SCN neurons,^27^ we next determined whether RCN2 rhythmic expression within the PVN is affected by light. However, we found that the rhythmicity of Raptin secretion persisted in constant darkness but was disrupted under SF conditions (Supplementary information, Fig. S5d), indicating that its secretion is regulated by the endogenous circadian activity of SCN^AVP^ neurons.

**Fig. 3:**
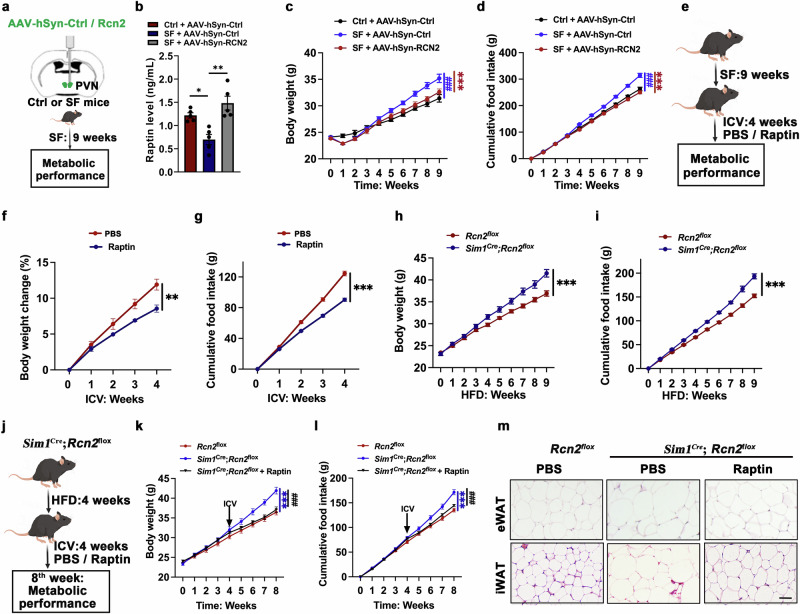
Hypothalamus-derived Raptin suppresses food intake and protects against obesity. **a** Schematic diagram of *Rcn2* overexpression in PVN of mice with SF intervention. AAV-hSyn-Ctrl or AAV-hSyn-Rcn2 was bilaterally injected into the PVN of WT male mice, followed by SF intervention for 9 weeks. **b** The plasma Raptin levels of 4-month male control or SF mice injected with AAV-hSyn-Rcn2 or AAV-hSyn-Ctrl (*n* = 5 per group). **c**, **d** Body weight (**c**) and cumulative food intake (**d**) of 4-month male control mice, SF mice and SF mice with *Rcn2* overexpression. ###*P* < 0.001 when control mice were compared to SF mice. ***P < 0.001 when SF mice were compared to SF mice with *Rcn2* overexpression (*n* = 5 per group). **e** Schematic diagram illustrating the 4-week ICV infusion of Raptin (40 ng/h/g body weight) or PBS in 4-month SF male mice. **f**, **g** Body weight change (**f**) and cumulative food intake (**g**) of 4-month SF male mice with ICV infusion of Raptin or PBS for 4 weeks (*n* = 4 per group). **h**, **i** Body weight (**h**) and cumulative food intake (**i**) of 2-month *Rcn2*^*flox/flox*^ and *Sim1*^*Cre*^*;Rcn2*^*flox/flox*^ male mice fed an HFD for 9 weeks (*n* = 6 per group). **j** Schematic diagram of 2-month *Sim1*^*Cre*^*;Rcn2*^*flox/flox*^ mice or *Rcn2*^*flox/flox*^ male mice with 8-week HFD and 4-week ICV infusion of Raptin (40 ng/h/g body weight) or PBS. **k**, **l** Body weight (**k**) and cumulative food intake (**l**) of *Rcn2*^*flox/flox*^ and *Sim1*^*Cre*^*;Rcn2*^*flox/flox*^ male mice with ICV infusion of PBS or Raptin (*n* = 6 per group). ***P < 0.001 when *Rcn2*^*flox/flox*^ mice were compared to *Sim1*^*Cre*^*;Rcn2*^*flox/flox*^ mice. ###*P* < 0.001 when *Sim1*^*Cre*^*;Rcn2*^*flox/flox*^ mice were compared to *Sim*^*Cre*^*;Rcn2*^*flox/flox*^ mice treated with Raptin. **m** Representative H&E staining of eWAT and iWAT of 4-month control mice or *Sim*^*Cre*^*;Rcn2*^*flox/flox*^ male mice with ICV infusion of PBS or Raptin. Scale bar, 100 μm (*n* = 6 per group). Data are shown as the mea*n* ± SEM. *P < 0.05, **P < 0.01, ***/###*P* < 0.001 by two-way ANOVA (**b**–**d**, **f**–**i**, **k**, **l**). See also Supplementary information, Figs. S5–S7.

To test whether hypothalamus-derived Raptin is involved in SF-induced obesity, we overexpressed *Rcn2* in the PVN of SF mice. We found that Raptin levels were elevated in *Rcn2*-overexpressing mice compared to SF male mice (Fig. 3a, b; Supplementary information, Fig. S5e–g). The overexpression of Raptin in the PVN significantly alleviated excessive weight gain and reduced food intake of SF mice to the levels seen in the control mice (Fig. 3c, d). In addition, PVN-specific *Rcn2* overexpression rescued the SF-induced impairment in glucose tolerance, insulin sensitivity and fat accumulation (Supplementary information, Fig. S5h–l).

To further validate the metabolic benefits of Raptin, we generated and purified recombinant mouse Raptin protein with a His-tag, whose validity was confirmed by silver staining (Supplementary information, Fig. S6a). We selected the dose of Raptin (40 ng/h/g body weight) for intracerebroventricular (ICV) infusion based on its elevated range in plasma during the sleep phase (Supplementary information, Fig. S6b). Moreover, either the ICV infusion of Raptin or intraperitoneal injection of Raptin had suppressive effects on appetite and gastric emptying (Supplementary information, Fig. S6c, d). Next, we performed ICV infusion of Raptin for 4 weeks in SF male mice to explore the effect of Raptin on SF-induced metabolic dysfunction (Fig. 3e). The sustained supplementation of Raptin successfully rescued the metabolic disturbance in SF mice, as indicated by less food intake and weight gain compared to vehicle-treated mice (Fig. 3f, g).

Previous studies revealed that high-fat diet (HFD)-induced obesity impairs the sleep–wake cycle.^28^ Consistent with those findings, we found that HFD-induced obese male mice exhibited more wake time and less non-rapid eye movement (NREM) sleep time than normal diet (ND)-fed mice (Supplementary information, Fig. S6e). Moreover, we found that HFD-induced obese mice had lower plasma Raptin levels than ND-fed mice (Supplementary information, Fig. S6f). Therefore, we applied ICV infusion of Raptin into HFD-induced obese male mice. Raptin treatment resulted in weight loss and appetite suppression in WT mice fed an HFD (Supplementary information, Fig. S6g–i). Pair feeding (caloric matching) of PBS-treated mice showed a similar body weight gain to Raptin-treated mice, indicating that the reduction in food intake is a major factor by which Raptin induces weight loss (Supplementary information, Fig. S6g–i).

To further verify that the appetite inhibition was mediated by direct effects in PVN but not the arcuate nucleus (ARC), we incubated brain slices from Cas9-EGFP[KI/+];*Pomc*-Cre male mice and Cas9-EGFP[KI/+];*AgRP*-Cre male mice with Raptin. Then, we detected the activity of POMC^+^ neurons and AgRP^+^ neurons (canonical neurons for appetite control in ARC)^29^ before and after Raptin treatment. The results indicated that Raptin failed to activate either the POMC^+^ neurons or the AgRP^+^ neurons (Supplementary information, Fig. S6j–m). Collectively, our results raised the possibility that Raptin has a prophylactic effect on obesity.

We then assessed the metabolic effects in PVN-specific *Rcn2* deletion male mice. *Rcn2* deficiency in the PVN resulted in significantly decreased Raptin level, especially in the sleep phase (Supplementary information, Fig. S7a). *Rcn2* deficiency in the PVN did not impair the sleep–wakefulness cycle as indicated by a nonsignificant change of wakefulness, NREM sleep duration and rapid eye movement (REM) sleep duration (Supplementary information, Fig. S7b). However, there was a slight increase in body weight in the PVN-specific *Rcn2* knockout male mice even during ND feeding (Supplementary information, Fig. S7c). When fed a HFD, PVN-specific *Rcn2* knockout mice showed a greater increase in body weight compared to littermate control mice (Fig. 3h). Compared to *Rcn2*^*flox*/*flox*^ mice fed an HFD, PVN-specific *Rcn2* knockout mice showed a greater increase in food intake both during the light phase or the night phase (Fig. 3i; Supplementary information, Fig. S7d). Additionally, PVN-specific *Rcn2* knockout male mice showed decreased oxygen consumption and energy expenditure during the dark period even though they were not changed in the day period or total period (Supplementary information, Fig. S7e–h). Importantly, no alterations of feeding-related hormones were detected in the PVN-specific *Rcn2* knockout male mic (Supplementary information, Fig. S7i–m). Moreover, there was no difference in the activity of POMC^+^ and AgRP^+^ neurons (Supplementary information, Fig. S7n–q), further indicating that RCN2 did not influence these canonical appetite-regulatory pathways.

To further confirm the effect of Raptin on body weight loss and appetite suppression, we performed a rescue experiment by ICV infusion of Raptin in PVN-specific *Rcn2*-deficient male mice (Fig. 3j). Sustained Raptin release effectively delayed the rapid weight gain caused by *Rcn2* deficiency and lowered food intake to the levels of *Rcn2*^*flox/flox*^ mice (Fig. 3k, l). By histological staining, we found that fat accumulation in epididymal white adipose tissue (eWAT) and inguinal white adipose tissue (iWAT) of the knockout mice was abolished by treatment with Raptin (Fig. 3m). In addition, Raptin rescued the impaired glucose homeostasis of the *Rcn2*-deficient mice (Supplementary information, Fig. S7r–u). Moreover, Raptin showed no effects on leptin and orexin A levels (Supplementary information, Fig. S7v, w) and did not influence the physical activity of mice (Supplementary information, Fig. S7x).

Together, these findings suggest that hypothalamus-derived Raptin inhibits food intake and prevents obesity.

### Raptin binds to GRM3 in neurons of the PVN and stomach to suppress food intake

**Fig. 4:**
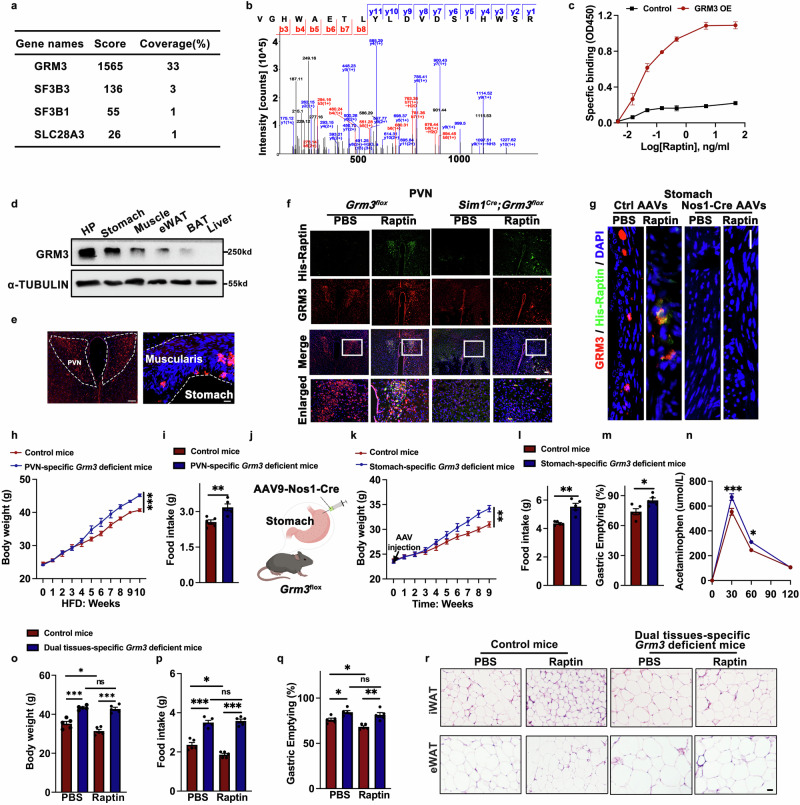
Raptin binds to GRM3 in neurons of the PVN and stomach to suppress food intake. **a** List of candidate membrane receptors of Raptin through MS analysis. The cell lysate of hypothalamic neurons was incubated with His-Raptin. The proteins pulled down by anti-His antibody were tested by MS analysis. **b** Representative mass spectrogram of GRM3 (The identified sequence of GRM3 peptide fragment: VGHWAETLYLDVDSIHWSR). **c** Effect of Raptin on GRM3 binding in HEK293T cells transfected either with control plasmid or *Grm3* plasmid. **d** Representative western blot of GRM3 expression in different tissues. **e** Representative images of GRM3 expression (red) in the PVN and muscular layer of the stomach. Scale bars, 50 μm. **f** Binding assay on frozen tissue sections of PVN from *Grm3*^*flox/flox*^ or *Sim1*^*Cre*^*;Grm3*^*flox/flox*^ mice. His-Raptin or PBS was pre-incubated with frozen tissue slices. Scale bar, 50 μm. **g** Binding assay on frozen tissue sections of the stomach from *Grm3*^*flox/flox*^ mice intragastrically injected with AAV-Ctrl or AAV-Nos1-Cre. His-Raptin or PBS was pre-incubated with frozen tissue slices. Scale bar, 50 μm. **h**, **i** Body weight (**h**) and 24-h food intake (**i**) of 4-month control (*Grm3*^*flo/flox*^ mice) and PVN-specific *Grm3*-deficient male mice (*Sim1*^*Cre*^*;Grm3*^*flox/flox*^ mice) with 10-week HFD (*n* = 5 per group). **j** Schematic diagram illustrating the injection of AAV-Nos1-Cre into the muscle layer of the stomach of 2-month *Grm3*^*flox/flox*^ male mice to generate stomach-specific *Grm3*-deficient mice. Control male mice are *Grm3*^*flox/flox*^ mice. **k**, **l** Body weight (**k**) and food intake (**l**) of 4-month control or stomach-specific *Grm3*-deficient male mice (*n* = 5 per group). **m** Gastric emptying of 4-month control or stomach-specific *Grm3*-deficient male mice measured via phenol red test (*n* = 5 per group). **n** Gastric emptying of 4-month control or stomach-specific *Grm3*-deficient male mice measured via acetaminophen absorption test (*n* = 5 per group). **o**–**q** Body weight (**o**), food intake (**p**) and gastric emptying (**q**) of 4-month controls (*Grm3*^*flox/flox*^ mice) and dual tissue-specific *Grm3*-deficient male mice (*Sim1*^*Cre*^*;Grm3*^*flox/flox*^ mice with injection of AAV-Nos1-Cre into stomach) with or without treatment of Raptin. Mice were fed an HFD. Raptin was injected into mice via the tail vein at a dose of 1 mg/kg body weight every other day for 8 weeks (*n* = 5 per group). **r** H&E staining of the eWAT and iWAT of the control and dual tissue-specific *Grm3*-deficient male mice treated with PBS or Raptin. Scale bar, 100 μm (*n* = 5 per group). Data are shown as the mean ± SEM. *P < 0.05, **P < 0.01, ***P < 0.001 by two-way ANOVA (**h**, **k**, **n**, **o**–**q**) or by a two-tailed unpaired Student’s *t*-test (**i**, **l**, **m**). See also Supplementary information, Figs. S8, S9.

To identify the receptor for Raptin, we performed MS analysis using cell lysate extracted from hypothalamic GT1-7 neurons incubated with His-labeled Raptin. This approach did not enrich our previously identified receptor complex (neuropilin-2 and integrin beta-1) from the bone marrow adipose tissues.^22^ However, we identified GRM3 as a new receptor of Raptin, and chose it for further evaluation because of its highest score and coverage among identified canonical receptors (Fig. 4a, b). The binding between Raptin and GRM3 was confirmed via saturation binding assay (Fig. 4c).

It has been reported that GRM3 is composed of an extracellular venus flytrap domain (VFT), a transmembrane domain (TMD) consisting of seven TM helices, and a cysteine-rich domain (CRD) connecting VFT and TMD.^30^ Therefore, we performed a molecular dynamics clustering analysis between GRM3 VFT and RCN2 and found a representative conformation with their binding detail. The GRM3 residues binding to RCN2 included Asn495, Ser479, Lys477, Arg59 and Gln360 (Supplementary information, Fig. S8a). In line with these results, we verified that Raptin bound to the VFT of GRM3 in GT1-7 cells through immunoprecipitation assay (Supplementary information, Fig. S8b). Moreover, immunoprecipitation assay and specific binding assay further showed that the mutation at N495 of GRM3 disrupted its binding to Raptin, suggesting that Asn495 is the key residue for this interaction (Supplementary information, Fig. S8c, d).

We next found that GRM3 was highly expressed in the hypothalamus and stomach (Fig. 4d, e). To further confirm the target tissue of Raptin, we performed binding assays on frozen tissue sections by incubating purified His-Raptin with different tissues. The results revealed that Raptin targeted GRM3 in the PVN and the muscular layer of the stomach (Fig. 4f, g; Supplementary information, Fig. S8e, f). In the PVN, like RCN2, GRM3 was mostly expressed in neurons, rather than in astrocytes or microglia (Supplementary information, Fig. S8g, h). However, the immunofluorescence colocalization between RCN2 and GRM3 was weak (Supplementary information, Fig. S8i), raising the possibility that Raptin functions via a paracrine manner. Therefore, we generated PVN-specific *Grm3* knockout mice (*Sim*^*Cre*^*;Grm3*^*flox/flox*^ mice) and verified the successful deletion of *Grm3* in the PVN (Supplementary information, Fig. S9a, b). We observed that Raptin displayed strong binding signals in the PVN, while the binding signals disappeared in the *Grm3-*deficient PVN (Fig. 4f).

The neurons innervating the gastrointestinal (GI) muscles are mainly CHAT-positive and NOS1-positive neurons.^31,32^ Thus, we performed co-staining of GRM3 with CHAT or NOS1 in the stomach and found that GRM3 was colocalized with NOS1-positive neurons (Supplementary information, Fig. S9c). Next, we generated a gastric NOS1-positive neuron-specific *Grm3* knockout mice by injecting AAV-Nos1-Cre virus into the muscular layer of the stomach in *Grm3*^*flox/flox*^ male mice. Consistent with the binding signals in the PVN, we confirmed that Raptin exhibited strong binding signals in the stomach of control male mice, while the binding signal disappeared in *Grm3*-deficient stomach neurons (Fig. 4g).

Next, we examined the phenotypes of *Grm3*-deficient mice. No obvious changes of wakefulness, NREM sleep duration and REM sleep duration were observed between control littermates and PVN-specific *Grm3*-deficient male mice (Supplementary information, Fig. S9d). However, the faster weight gain in *Sim1*^*Cre*^*;Grm3*^*flox/flox*^ male mice was observed under ND feeding (Supplementary information, Fig. S9e). With an HFD feeding, *Sim1*^*Cre*^*;Grm3*^*flox/flox*^ male mice exhibited greater body weight gain compared to control male littermates (Fig. 4h). Consistent with these findings, PVN-specific *Grm3* knockout mice showed a greater increase in food intake compared to littermate control mice both during the light phase and night phase (Fig. 4i; Supplementary information, Fig. S9f). Moreover, the body weight and food intake were also greater in the stomach-specific *Grm3*-deficient mice compared to the *Grm3*^*flox/flox*^ mice (Fig. 4j–l). As GI motility is important for appetite and weight control,^33^ we measured gastric motility and found that *Grm3* deletion resulted in accelerated gastric emptying (Fig. 4m, n). To further explore the effects of GRM3 in the PVN and stomach, we injected AAV-Nos1-Cre virus into the stomach of *Sim1*^*Cre*^*;Grm3*^*flox/flox*^ male mice to obtain mice with *Grm3* knockout both in the PVN and stomach (dual tissue-specific *Grm3* knockout mice). *Grm3*^*flox/flox*^ mice injected with the control virus were used as controls. The dual tissue-specific *Grm3*-deficient mice showed a higher body weight and food intake compared to either single PVN-specific *Grm3*-deficient male mice or single stomach-specific *Grm3*-deficient male mice (Supplementary information, Fig. S9g, h).

To further confirm the effects of Raptin mediated by GRM3, we administered recombinant Raptin in the dual tissue-specific *Grm3*-deficient male mice. We found that Raptin decreased body weight, gastric emptying, food intake and fat accumulation in control *Grm3*^*flox/flox*^ male mice fed a HFD (Fig. 4o–r). However, the Raptin-mediated effects were blocked in the dual tissue-specific *Grm3*-deficient male mice, which exhibited no change in body weight, gastric emptying, food intake and fat accumulation when treated with Raptin (Fig. 4o–r). Regarding metabolic alterations, Raptin-induced improvements of glucose tolerance and insulin sensitivity were blocked in the dual tissue-specific *Grm3*-deficient male mice (Supplementary information, Fig. S9i–l). Of note, Raptin administration decreased body weight and food intake in control female mice fed an HFD, but not in the dual tissue-specific *Grm3*-deficient female mice (Supplementary information, Fig. S9m, n). Furthermore, *Rcn2* overexpression in the PVN of mice inhibited appetite, gastric emptying and decreased body weight, which were weakened in stomach-specific *Grm3*-deficient mice (Supplementary information, Fig. S9o–q).

Together, these results suggest that PVN-derived Raptin inhibits appetite and gastric emptying by binding GRM3 in the neurons of the PVN and the stomach, which ultimately leads to a reduction in body weight.

### Raptin-GRM3 signaling maintains the energy supply for neuron activation and anorexigenic effects

Next, we investigated whether Raptin signaling directly affects neuronal activity in a GRM3-dependent manner. To determine that GRM3-positive neurons in the PVN (PVN^GRM3^ neurons) play critical roles in appetite control, we firstly constructed AAVs carrying a 2.3 kb region of the *Grm3* promoter and an *EGFP* reporter gene (AAVs-Grm3-Flp-EGFP). By ISH utilizing a *Grm3* RNA probe, we verified the effectiveness of these viruses in marking the GRM3-positive neurons in the PVN (Supplementary information, Fig. S10a, b).

**Fig. 5:**
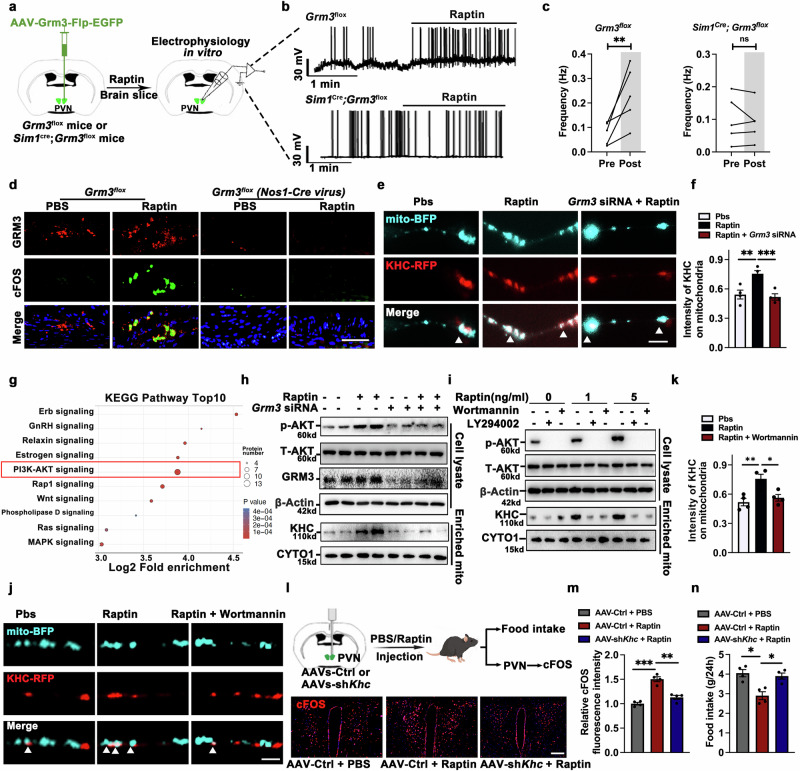
Raptin-GRM3 signaling maintains the energy supply for neuron activation and anorexigenic effects. **a** Schematic diagram illustrating the injection of AAV-Grm3-Flp-EGFP into PVN of control and PVN-specific *Grm3*-deficient male mice to label PVN^GRM3^ neurons, followed by neuronal activity recording of PVN^GRM3^ neurons in brain slice before and during Raptin treatment. Raptin was dissolved in ACSF at a concentration of 1 ng/mL and incubated with brain slices for 10 min. **b**, **c** Representative traces (**b**) and action potential frequency (**c**) of PVN^GRM3^ neurons from control and PVN-specific *Grm3*-deficient male mice (*n* = 5 per group). **d** Representative images of c-Fos (green) in the GRM3^+^ neurons of the stomach from control and stomach-specific *Grm3*-deficient male mice followed by administration of Raptin via tail vein at a dose of 1 mg/kg body weight for 1 h. Scale bar, 50 μm. **e** Representative images of labeled mitochondria (cyan) and KHC (red) in primary hypothalamus neurons transfected with control or *Grm3* siRNA, followed by treatment of Raptin or PBS. These neurons were transfected with Mito-BFP and Khc-RFP plasmids to label mitochondria and KHC protein, respectively. Scale bar, 5 μm. **f** Quantification of the fluorescent KHC present on mitochondria, defined by the intensity of KHC in the region overlapping Mito-BFP (*n* = 4 per group). **g** KEGG analysis of changed phosphorylation in the hypothalamic GT1-7 neurons through global quantitative phosphoproteomic analysis. Hypothalamic GT1-7 neurons were treated with PBS or Raptin at 10 ng/mL for 1 h. **h** Representative western blot of AKT signaling in cell lysates and KHC expression in mitochondria enriched from primary neurons treated with Raptin or *Grm3* siRNA. Raptin was used at 10 ng/mL for 1 h. CYTO1 was used as the internal control of mitochondria. **i** Representative western blot of AKT signaling in cell lysates and KHC expression in mitochondria enriched from primary neurons treated with Raptin or PI3K-AKT pathway inhibitors (Wortmannin and LY294002). CYTO1 was used as the internal control of mitochondria. **j**, **k** Representative images (**j**) and quantification (**k**) of the fluorescent KHC (red) present on mitochondria, defined by the intensity of KHC in the region overlapping Mito-BFP (cyan) in primary hypothalamus neurons. Scale bar, 5 μm (*n* = 4 per group). **l**, **m** Schematic diagram illustrating the injection of AAV-hSyn-Ctrl or AAV-hSyn-sh*Khc* in the PVN of 2-month WT male mice to generate the control and PVN-specific *Khc* knockdown mice, followed by treatment of PBS or Raptin. Representative immunofluorescence images (**l**) and quantification (**m**) of c-Fos (red) expression in PVN. Scale bar, 50 μm. **n** 24-h food intake of the PVN-specific *Khc* knockdown mice and control mice with or without treatment of Raptin (*n* = 4 per group). Data are shown as the mean ± SEM. *P < 0.05, **P < 0.01, ***/###*P* < 0.001 by a two-tailed paired Student’s *t*-test (**c**) or by two-way ANOVA (**f**, **k**, **m**, **n**). See also Supplementary information, Figs. S10–S12.

PVN^GRM3^ neurons help better define the effect of Raptin on GRM3 signaling. Therefore, we injected AAV-Grm3-Flp-EGFP into the PVN of *Grm3*^*flox/flox*^ and *Sim1*^*Cre*^*;Grm3*^*flox/flox*^ mice to label PVN^GRM3^ neurons. By patch-clamp electrophysiology analysis in vitro, we found that EGFP-labeled PVN^GRM3^ neurons were activated in the presence of Raptin (1 ng/mL; in control artificial cerebrospinal fluid (ACSF)) in the brain slice of *Grm3*^*flox/flox*^ mice, but not in *Sim1*^*Cre*^*;Grm3*^*flox/flox*^ mice (Fig. 5a–c). Similarly, c-Fos staining revealed that Raptin could activate NOS1^+^ neurons in the stomach, while *Grm3* deficiency abolished this effect of Raptin (Fig. 5d).

To determine that PVN^GRM3^ neurons played critical roles in appetite control, we manipulated PVN^GRM3^ neurons by injecting an excitatory chemogenetic virus (AAV-DIO-hM3Dq-mCherry) and AAV-Grm3-Cre into the PVN (Supplementary information, Fig. S10c). The AAV-Grm3-Cre-EGFP to mark the GRM3-positive neurons in the PVN was confirmed by ISH (Supplementary information, Fig. S10d, e). By this approach, we found an inhibition of appetite along with no change of gastric emptying upon activation of GRM3^+^ neurons in the PVN after intraperitoneal injection of 2 mg/kg CNO (Supplementary information, Fig. S10f, g), suggesting that the PVN pathway was not directly communicating with the stomach. However, the CNO-induced activation of GRM3^+^ neurons in the stomach resulted in appetite suppression along with reduced gastric emptying (Supplementary information, Fig. S10h–l).

To elucidate the mechanism of neuronal activation, we conducted a global quantitative phosphoproteomic analysis of hypothalamic neurons treated with Raptin or PBS. By COG analysis of the differentially phosphorylated proteins, we found obvious alterations of transport and catabolism, as well as cell motility (Supplementary information, Fig. S11a). Considering that mitochondrial movement is critical for energy distribution to sustain neuron activity,^34^ we sought to investigate whether mitochondrial motility was involved in the energy supply of Raptin-stimulated neuron activation. Kinesin heavy chain (KHC) serves as a primary anterograde motor for the axonal transport of mitochondria and governs aspects of mitochondrial distribution in neurons, and the localization of KHC in the mitochondria suggests the increased mitochondrial movement.^35^ Therefore, we transfected primary hypothalamic neurons with mito-BFP and *Khc*-RFP plasmids. We observed that Raptin treatment increased the colocalization of KHC and mitochondria, while this effect was abolished in *Grm3*-deficient neurons (Fig. 5e, f).

To identify the signaling pathway involved in mitochondrial movement, we conducted a KEGG analysis using phosphoproteomic data. Among the top 10 pathways, the PI3K-AKT pathway was remarkably impacted by Raptin (Fig. 5g). To assess the intrinsic potency of Raptin in vitro, GT1-7 cells expressing GRM3 were used to measure p-AKT accumulation as the downstream signaling of receptor activation (Raptin 50% effective concentration [EC_50_] = 0.31 ng/mL) (Supplementary information, Fig. S11b). We then verified that Raptin activated PI3K-AKT signaling in control neurons but not in neurons with siRNA-mediated knockdown of *Grm3* (Fig. 5h). Previous studies revealed that the primary motor complex, formed by KHC binding to MIRO1, regulated mitochondrial transport.^35,36^ KHC detection in the mitochondrial fraction reflected the mitochondrial mobility.^37^ Thus, we examined the levels of KHC heavy chain in the mitochondrial fraction and found that their levels were increased by Raptin treatment in control neurons but not in the *Grm3* knockdown neurons (Fig. 5h). Moreover, treatment with Wortmannin and LY294002 (PI3K inhibitors) effectively abolished the Raptin-induced increase of KHC associated with mitochondria (Fig. 5i).

Considering that axonal mitochondria serve as local power stations for ATP supply to sustain prolonged neural activity, we performed live imaging experiments to detect ATP levels in mitochondria in hypothalamic neurons. ATP levels were assessed using genetically encoded Förster resonance energy transfer (FRET)-based ATP probe pCMV-AT1.03.^38^ The relative intensity ratio of pCMV-AT1.03 YFP (ATP-bound) to CFP (ATP-free) reflected the ATP levels. We found that Raptin treatment resulted in greater ATP levels in mitochondria of control neurons but not in *Grm3*-deficient neurons (Supplementary information, Fig. S11c, d). Consistently, Wortmannin treatment abolished the Raptin-induced distribution of KHC in mitochondria (Fig. 5j, k) and increase of mitochondrial ATP levels (Supplementary information, Fig. S11e, f). Thus, these findings suggested that Raptin promoted GRM3^+^ neuron activity via PI3K-AKT signaling.

Previous studies showed that glutamate, as a classical endogenous ligand of GRM3, can affect neuronal activity through inhibiting cyclic AMP (cAMP) signaling.^39^ Hence, we compared the difference between glutamate and Raptin regarding the downstream signaling. Time-resolved FRET (trFRET) measurements showed the affinity between GRM3 and Raptin or glutamate in HEK293 cells (Supplementary information, Fig. S12a). However, glutamate inhibited cAMP signaling but did not affect AKT signaling, whereas Raptin promoted AKT signaling but did not affect cAMP signaling (Supplementary information, Fig. S12b–d). In addition, we treated GT1-7 cells with Raptin and GRM3 receptor agonists (LY354740, LY2794193 and LY379268), inhibitors (LY341495) or allosteric agents (VU0650786), respectively. We found that none of the GRM3 receptor agonists, inhibitors or allosteric agents could alter Raptin-GRM3-mediated AKT signaling (Supplementary information, Fig. S12e, f). Together, glutamate and Raptin did not show mutual effects on the downstream AKT signaling.

To further explore whether KHC was indispensable in GRM3-mediated neuronal activation and appetite control, we injected Raptin or PBS into mice with or without knockdown of *Khc* in the PVN (Fig. 5l). The knockdown efficiency was confirmed in isolated PVN region (Supplementary information, Fig. S12g). We found that Raptin treatment activated neurons in the PVN, however, this effect was impaired by the knockdown of *Khc* (Fig. 5l, m). Moreover, the effect of Raptin on appetite inhibition was also diminished in PVN-specific *Khc* knockdown mice (Fig. 5n). We found that LY354740 treatment in the PVN did not affect appetite in either control mice or PVN-specific *Khc*-deficient mice (Supplementary information, Fig. S12h, i). In addition, LY354740 treatment did not affect food intake or gastric emptying in either control mice or stomach-specific *Khc*-deficient mice (Supplementary information, Fig. S12j–l). Furthermore, we chemogenetically manipulated PVN^GRM3^ neurons with or without knockdown of *Khc* in the PVN (Supplementary information, Fig. S12m). Intriguingly, CNO-induced excitatory stimulation suppressed food intake in control mice, but this effect was attenuated in the PVN-specific *Khc* knockdown mice (Supplementary information, Fig. S12n). CNO-induced excitatory stimulation showed a similar trend in terms of effect on food intake, along with altered gastric emptying, in the mice with *Khc* knockdown in the stomach (Supplementary information, Fig. S12o–q).

In conclusion, we propose that Raptin-Grm3 signaling promotes the PI3K-AKT pathway, playing a crucial role in the KHC-mediated energy supply for neuron activation and appetite control.

### Raptin dysregulation is involved in sleep deficiency-induced obesity in humans

**Fig. 6:**
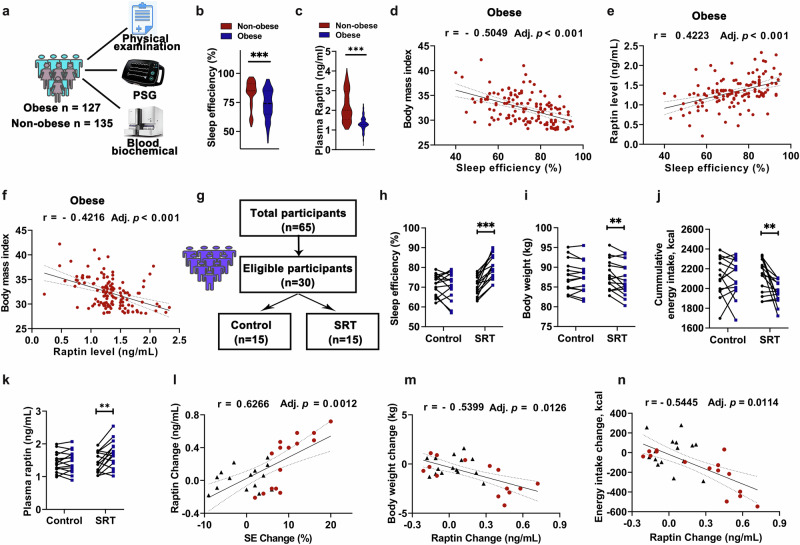
Raptin is involved in sleep deficiency-induced obesity in humans. **a** Schematic diagram of the cross-sectional study evaluating the effect of sleep on obesity. **b**, **c** Violin plot of sleep efficiency (**b**) and plasma Raptin levels (**c**) in participants with or without obesity (*n* = 127 for obese group, *n* = 135 for non-obese group). **d** Spearman’s correlation between SE and BMI in participants with obesity (*n* = 127 for obese group). **e** Spearman’s correlation between SE and Raptin levels in participants with obesity (*n* = 127 for obese group). **f** Spearman’s correlation between Raptin levels and BMI in participants with obesity (*n* = 127 for obese group). **g** Schematic diagram illustrating the workflow of a prospective study evaluating the effect of SRT on obesity (*n* = 15 per group). **h**–**k** Change of SE (**h**), body weight (**i**), cumulative energy intake (**j**) and plasma Raptin level (**k**) between control and SRT groups. **l** Spearman’s correlation between SE change and plasma Raptin level change in participants with obesity. The triangle represents the control group, and the circle represents the SRT group. **m**, **n** Spearman’s correlation between plasma Raptin level change and changes of body weight (**m**) and energy intake (**n**) in participants with obesity. The triangle represents the control group, and the circle represents the SRT group. Data are shown as the mean ± SEM or β estimate ± 95% CI. **P < 0.01, ***P < 0.001 by Mann–Whitney U test (**b**, **c**), Spearman’s correlation analysis (**d**–**f**, **l**–**n**) or a two-tailed paired Student’s *t*-test (**h**–**k**). The *P* values of Spearman correlation analysis were corrected by Bonferroni method. See also Supplementary information, Fig. S13.

Regarding clinical relevance, we sought to investigate the effects of sleep quality on the risk of obesity in humans. Therefore, we analyzed the interplay among sleep quality, Raptin release and obesity within a cohort of 262 participants that were matched for age and sex. Among these participants, 127 presented with obesity (BMI: 32.30 ± 3.00), while the remaining 135 were not obese (BMI: 22.0 ± 2.4) (Fig. 6a). Comparative analysis demonstrated that individuals with obesity exhibited lower sleep quality as indicated by reduced total sleep time (TST) and sleep efficiency (SE) along with elevated wakefulness after sleep onset (WASO) and sleep latency (SL), in contrast to their non-obese counterparts (Fig. 6b; Supplementary information, Fig. S13a–c). Moreover, participants with obesity showed significantly lower plasma Raptin levels (Fig. 6c).

We further conducted a detailed examination of these correlations by analyzing the non-obese or obese group independently. In the non-obese population, we did not observe obvious correlations between BMI and SE (Supplementary information, Fig. S13d). Moreover, Raptin levels showed no significant correlation with BMI and sleep quality indicators including TST, SE, WASO and SL (Supplementary information, Fig. S13e–i). However, in the population with obesity, we observed a negative correlation between SE and BMI (Fig. 6d). Except for TST, other sleep quality indicators (SE, SL and WASO) were correlated with Raptin levels in the population with obesity (Fig. 6e; Supplementary information, Fig. S13j–l). Consistently, we observed an obvious negative correlation of BMI with Raptin levels in the population with obesity (Fig. 6f). These intricate associations indicated a nuanced relationship between sleep quality, Raptin release, and obesity. Collectively, these results suggest that sleep deficiency may disrupt Raptin release, particularly in cases of obesity.

Given the established association between disturbed sleep quality and development of obesity, we explored whether improved sleep quality in patients with obesity and insomnia could promote Raptin release and inhibit food intake. SRT,^40,41^ a recognized treatment for long-term insomnia in clinical settings, was employed for this investigation. We conducted a prospective cohort study by recruiting male patients with obesity and insomnia. The participants were divided into a control group without any intervention (*n* = 15) and an intervention group treated with 3-month SRT regimen (*n* = 15) (Fig. 6g). The age and BMI of the control group (age: 31.7 ± 5.1, BMI: 30.0 ± 1.2) and SRT group (age: 32.1 ± 5.9, BMI: 30.4 ± 2.1) were matched. As expected, patients undergoing SRT exhibited substantial improvements in SE (Fig. 6h), a reduction in body weight and energy intake (Fig. 6i, j), as well as an increase in plasma Raptin levels (Fig. 6k). Moreover, the change of Raptin levels displayed a positive correlation with indicators of improved SE (Fig. 6l), and negative correlations with reduction of body weight and energy intake (Fig. 6m, n). These data suggest a deep connection among sleep patterns, plasma Raptin levels and obesity in humans.

### A *RCN2* nonsense mutation contributes to night-eating syndrome

**Fig. 7:**
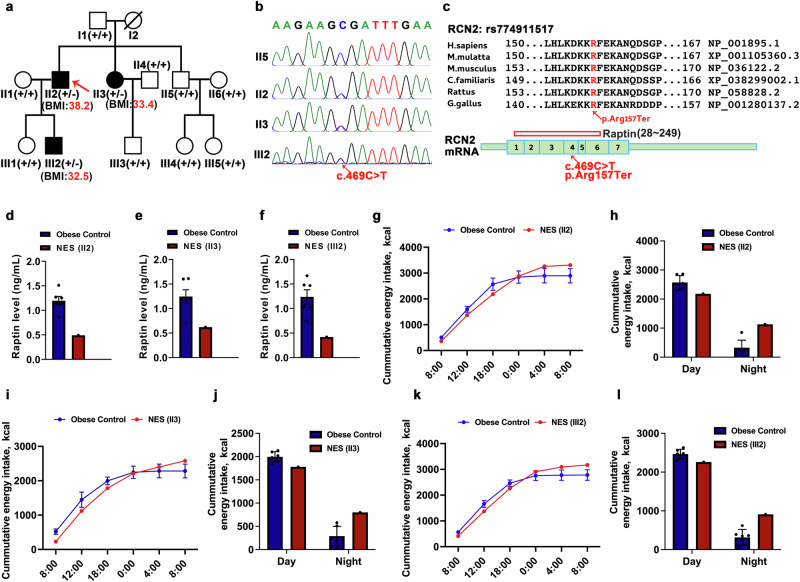
An *RCN2* nonsense mutation contributes to NES. **a** Pedigree of the family with NES. The red arrow indicates the proband in this family. WT *RCN2* genotypes are depicted as +/+, and heterozygous *RCN2* genotypes are depicted as +/–. Numbers in red brackets indicate the BMIs of three affected obese patients. **b** Sanger sequencing chromatograms from II2, II3, III2 and one representative family member (II5). The c.469 C > T transition is indicated by the red arrow. **c** Protein homology of the neighboring region of the variant among selected species. The arrow shows the location of the p.Arg157Ter substitution. The red amino acid residue indicates the altered residue by the above variant found in this family. **d**–**f** Plasma Raptin levels of NES patients (II2, **d**; II3, **e**; III2, **f**) and their respective obese control (age-, sex- and BMI-matched, *n* = 6) during night phase. **g**, **h** The cumulative energy intake curves (**g**) and quantification (**h**) of II2 and his obese control (age-, sex- and BMI-matched, *n* = 6) during day and night phases. **i**, **j** The cumulative energy intake curves (**i**) and quantification (**j**) of II3 and her obese control (age-, sex- and BMI-matched, *n* = 6) during day and night phases. **k**, **l** The cumulative energy intake curves (**k**) and quantification (**l**) of III2 and his obese control (age-, sex- and BMI-matched, *n* = 6) during day and night phases. Data are shown as the mean ± SEM. See also Supplementary information, Fig. S13.

To further investigate the genetic evidence linking *RCN2* to obesity, we conducted Sanger sequencing to screen for *RCN2* variants in 2000 Chinese participants with obesity. Notably, we identified a heterozygous *RCN2* nonsense variant that co-segregated with obesity within a specific family, as indicated by this *RCN2* variant observed in the proband with obesity (II2) and two other family members with obesity (II3 and III2) but not in other family members (Fig. 7a, b). This variant, c.469 C > T, in *RCN2* was predicted to result in the substitution to a premature termination codon at site Arg157 within the exon 4 of RCN2 (p.Arg157Ter) (Fig. 7c). Moreover, the neighboring region of this mutation is highly conserved among vertebrates (Fig. 7c). To verify the loss of functionality of this *RCN2* variant, we overexpressed the human WT *RCN2* and this *RCN2* variant in HEK293T cells. By western blotting, we found a Raptin fragment with small molecular weights in cell lysate but not in concentrated culture medium (Supplementary information, Fig. S13m), suggesting that the Raptin fragment could not be secreted to bind GRM3 to exert the downstream effects. The co-immunoprecipitation assay showed no His-tagged RCN2 binding to GRM3 VFT in the cells transfected with this *RCN2* variant (Supplementary information, Fig. S13m). Moreover, the conditioned medium from hypothalamic neurons overexpressing WT *RCN2* promoted downstream AKT signaling, but this effect disappeared in neurons expressing the mutant *RCN2* (Supplementary information, Fig. S13n).

Notably, all three patients with obesity carrying this variant suffered from night-eating syndrome (NES), characterized by nocturnal awakenings for food intake in a fully conscious state, as reported in the questionnaires. This variant was associated with an increase in BMI, waist-to-hip ratio (WHR), and body fat in patients with NES within this family (Supplementary information, Fig. S13o). Additionally, their serum triglycerides were elevated compared to the unaffected controls (Supplementary information, Fig. S13o).

Individuals with NES showed lower plasma Raptin levels compared to other family members (Supplementary information, Fig. S13o). Compared to age- and BMI-matched participants with obesity, the three patients with NES showed lower plasma Raptin levels during the night sleep phase (Fig. 7d–f). To assess food intake, we monitored the total calorie intake within a 24-h period and observed a specific increase in nocturnal food intake in the patients with NES (II2, II3 and III2) compared to their sex-, age- and BMI-matched participants during the night, respectively (Fig. 7g–l). These results suggest that impaired Raptin release might be an important contributor to NES in this family.

## Discussion

The history of endocrinology is the history of hormone discovery and their functional evaluation.^42–44^ The hypothalamus, as a central hub of the neuroendocrine system and interoception, can secrete hormones in response to metabolic signals, thus playing crucial roles in energy balance.^45,46^ For decades, scientists have been dedicated to identifying new hypothalamic hormones, with the aim to elucidate the central and peripheral networks that underlie the neuroendocrine system as a way to identify new approaches for the treatment of obesity.^46,47^ By doing so, the genetic basis of rare forms of hereditary obesity has been identified, key drivers of more common cases of obesity, such as leptin resistance, have come into focus, and new ideas for successfully targeting obesity, including GLP1 receptor agonism, have been confirmed. But even so, the identification of new hypothalamic hormones that regulate food intake and thus adiposity is still of keen interest. Here, we identified a sleep-induced hypothalamic hormone that plays a role in promoting anorexigenic effects through GRM3-dependent signaling.

Poor sleep habits contributed to overeating and obesity, which is associated with dysrhythmic release of hormones.^2,48–50^ The idea in the field behind this is that disruptions in the circadian rhythm lead to overeating in the wrong phase of the day.^5,51^ Here, we identified the rhythmic hormone Raptin, which we found peaks during sleep but is depleted by disrupted sleep, as one that maintains the low desire for energy intake during the sleep phase. Thus, our results offer new insights into how the disrupted circadian rhythm leads to an increase in appetite.

The circadian network regulates the release of hormones, which can affect the behavioral circadian rhythm in turn, such as eating, sleeping, locomotion and so forth.^52,53^ Here, we showed that the circadian release of hypothalamic Raptin decreases appetite but does not affect other important circadian behaviors, including the sleep cycle itself or physical activity. Therefore, further studies are still needed to elucidate whether Raptin derived from other locations affects unappreciated circadian behaviors or physiological processes during homeostasis or other specific states.

Consistent with the reported mechanism of canonical appetite hormones, including leptin and GLP1,^11,20,54,55^ Raptin acted locally and distally via binding to its specific receptor in the neurons of the PVN and the stomach, respectively, thereby suppressing appetite and ameliorating weight gain. Thus, another interesting observation of this study is that we identified GRM3 as the receptor of Raptin to regulate gastric emptying and appetite. GRM3 is a member of the group II of G-protein coupled receptors (GPCRs) of glutamate, which is widely expressed in the central nervous system.^56^ Ligand binding to GRM3 can cause inhibition of adenylyl cyclase cascade.^56^ Moreover, glutamate signaling in specific regions has been reported to promote appetite.^57^ Different from these findings, we identified Raptin as a novel ligand of GRM3 to suppress food intake through AKT signaling. Moreover, Raptin-mediated AKT signaling and glutamate-mediated cAMP signaling did not show mutual effects. Our findings give a hint to explain the diverse GRM3-mediated effects mediated by Raptin or glutamate. On the one hand, the key binding residues of GRM3 for its known ligands are different from the key residue for Raptin binding,^58^ which might cause differential conformational changes of GRM3. On the other hand, it is well established that GPCRs can mediate different downstream signalings by coupling different G proteins (including G_s_, G_i_, G_q_).^59^ GPCR coupling to G_i_ can inhibit cAMP cascade while their coupling to G_q_ can stimulate the AKT pathway.^59^ Previous studies have reported that GRM3-mediated signaling results in the inhibition of cAMP signaling and activation of AKT signaling.^60,61^ The glutamate–GRM3 complex inhibits the cAMP cascade by coupling to G_i_.^56^ However, whether the Raptin–GRM3 complex couples to specific G proteins to control the AKT pathway and inhibit appetite needs to be further explored.

Of note, GRM3 is expressed in various metabolic organs, such as the hypothalamus, stomach, muscle, and fat tissues. Here, we showed that Raptin-GRM3 signaling in the PVN and stomach markedly affects appetite. In addition, given that leptin from fat tissues is a canonical hormone involved in the appetite control, we tested the leptin concentration in PVN-specific *Rcn2* knockout mice. The unchange of plasma leptin levels suggested that Raptin-GRM3 signaling in adipose tissues does not affect appetite via alterations in leptin. However, whether Raptin regulates appetite and body weight through the skeletal muscle or fat tissues still needs to be explored.

Of clinical significance, firstly, numerous experimental and approved clinical medicines targeting specific receptors (such as MC4R, GLP1R and LepR) are currently being targeted to treat obesity.^62–65^ In this respect, it would be worthwhile to utilize the newly identified receptor GRM3 for further anti-obesity drug discovery. Secondly, we revealed the association of sleep-affecting Raptin levels with human metabolic obese traits. Participants suffering from sleep deficiency exhibited lower Raptin levels and aggravated obesity, whereas patients with obesity that underwent SRT had higher Raptin levels and alleviated obese phenotypes. These findings further emphasize the importance of sleep quality on metabolic homeostasis. Thirdly, we found that individuals with *RCN2* nonsense variant suffered from obesity. Our study confirmed that the protein encoded by *RCN2* nonsense variant could not be secreted, and thus could not bind to GRM3 to exert the downstream effects. In addition, given that individuals with the heterozygous variant showed obese phenotypes, we could not exclude the possibility that mutated Raptin affected WT Raptin levels in a dominant-negative fashion. Further exploration regarding the mechanism by which this heterozygous variant is involved in obesity is warranted.

Taken together, our study identify Raptin as a unique hypothalamic hormone that cooperates with GRM3 to suppress appetite and obesity, thus providing a potential new avenue to treat obesity.

## Materials and methods

### Mice

C57BL/6J mice were obtained from Hunan SJA Laboratory Animal Company (Hunan, China). *Grm3*^*flox/flox*^ mice (Cat# S-CKO-00640), Cas9-EGFP[KI/+] mice (Cat# C001475), *Pomc*-Cre mice (Cat# C001448) and *AgRP*-Cre mice (Cat# C001249) were purchased from Cyagen Bioscience. The *Avp*-Cre mice (Cat# 023530) were purchased from the Jackson Laboratory. *Sim1*-Cre mice were designed and constructed by Cyagen Bioscience. To generate *Sim1*-Cre mice, a PCR amplicon containing an optimized internal ribosome entry sequence fused to Cre recombinase was inserted 3 bp after the *Sim1* stop codon. *Rcn2*^*flox/flox*^ and the complete *Rcn2* knockout mice were purchased from BRL Medicine Inc. To generate *Rcn2*-floxed mice, two flox sequences were inserted into the two termini of *Rcn2* exon 2 by using CRISPR/Cas9 technology. For genotyping, related reagent (AG12201) was purchased from ACCURATE BIOTECHNOLOGY(HUNAN) CO.,LTD, ChangSha, China. The genomic DNA was extracted from tail tips, and the primers of genotyping are:

*Rcn2*-L-loxp-F: TTCGCTCTAGGAGACCCTG;

*Rcn2*-L-loxp-R: GTCTTTGCTGCTCTTCGTG.

*Rcn2*-R-loxp-F2: AGTTCTTATGCCTGGAAGGTG;

*Rcn2*-R-loxp-R2: AAAGGTTTGGGCAAGTGTC.

*Grm3*-loxp-F: ATGTGGCACTCAGTTCCCAAATTA;

*Grm3*-loxp-R: GTCCTGCTATAGCTCAGTAGACAA.

*Sim1*-Cre-F1: TGGTTATGCCCTGGAACACTTAT

*Sim1*-Cre-R1: CCTTCCATGTGGTATACATGCTT

*Sim1*-Cre-F2: GCATGCCTATACATTGACTGGTTAT

*Sim1*-Cre-R2: CTTTGGCGAGAGGGGAAAGAC

*Rcn2*-KO-F: GAGAGGCCTTAGCAAGAGCG;

*Rcn2*-KO-R: AAGGTTTGGGCAAGTGTCTC.

Cas9-EGFP-F1: TGAGCGACATCCTGAGAGTG;

Cas9-EGFP-R1: GAGAGCTTTCAGCAGGGTCA.

Cas9-EGFP-F2: AAGGGAGCTGCAGTGGAGTA;

Cas9-EGFP-R2: CCGAAAATCTGTGGGAAGT.

*Pomc*-Cre-F: GCGGTCTGGCAGTAAAAACTATC;

*Pomc*-Cre-R: GTGAAACAGCATTGCTGTCACTT.

*AgRP*-Cre-F: GCTTCTTCAATGCCTTTTGC;

*AgRP*-Cre-R: AGGAACTGCTTCCTTCACGA.

The mice were housed in standard, specific pathogen-free facility at the Laboratory Animal Research Center of Central South University with controlled temperature (22–24 °C) and a 12-h dark/light cycle. The mice had free access to standard food (Hunan SJA Laboratory Animal Company, China) or high-fat food (Biopike, D12492) and water. The mice were maintained in good health and utilized for in-house mating to ensure an adequate supply for experiments. All animal care protocols and experiments were reviewed and approved by the Animal Care and Use Committees of the Laboratory Animal Research Center at Xiangya Medical School of Central South University.

### Human sample studies

The participants were recruited from Xiangtan First People’s Hospital. Blood samples from participants were centrifuged at 4 °C soon after sampling, then separated and stored at –80 °C. The studies were approved by the Ethics Committee at Xiangtan First People’s Hospital and written informed consents were obtained from all participants before collecting clinical data and samples (Ethical no. 2023110814, Ethical no. 2023063010). Plasma levels of Raptin and other biochemical index were measured using MS analysis and Sandwich ELISA assay.

### Human brain tissues

Human brain tissues used in the study were obtained from Human Brain Bank of Xiangya School of Medicine, Central South University. The studies were approved by the Ethics Committee at School of Basic Medical College, Central South University (Ethical no. 2020KT-37).

### Cell culture

Hypothalamic GT1-7 neurons and HEK293T cells were cultured in DMEM (Procell, PM150210) containing 10% fetal bovine serum (FBS), 100 U/mL penicillin (Pen), 100 μg/mL streptomycin (Strep) at 37 °C in a 5% CO_2_ humid atmosphere. Primary hypothalamus neurons were prepared from E18/19 mouse embryos as previously described.^66^ Briefly, the hypothalamus was dissected from E18/19 mouse embryos and kept in ice-cold dissection buffer containing HBSS (without CaCl_2_ and MgCl_2_), 1 M HEPES, and 1% Pen/Strep (v/v). A single-cell suspension was obtained through papain dissociation. Cells were then resuspended in neuronal plating media, composed of neurobasal media supplemented with 2% B27, 0.5 mM L-GlutaMAX, 55 μM β-mercaptoethanol, 10% FBS (all from Thermo Fisher Scientific), and 30 μg/mL insulin (Sigma Aldrich). The cell culture was maintained at 37 °C in a humidified incubator chamber under a 5% CO_2_ atmosphere.

For the experiments of glutamate treatment, GT1-7 neurons were cultured in DMEM supplemented with 10% fetal calf serum. To maintain a low concentration of ambient glutamate, cells were co-transfected with the cDNA of the glutamate transporter EAAC1 and incubated in DMEM Glutamax medium for at least 2 h before the different drugs were administered. Then GT1-7 cells were treated with glutamine-free medium containing 10 ng/mL Raptin or 50 μM glutamate for 30 min.

For the experiments of testing Raptin accumulation in cell lysates, the cells were treated with brefeldin A (5 ng/mL) plus monensin (5 ng/mL).

For the experiments testing Raptin accumulation in cell culture medium, the medium was collected and concentrated via ultrafiltration tube. The aprotinin (HY-P0017), LY2794193 (HY-119243), LY379268 (HY-103558), LY341495 (HY-70059) and VU0650786 (HY-108710) were purchased from MCE.

### SF model

For construction of SF mouse model, the intervention was performed as previously described.^67^ Each mouse was placed in an SF separate chamber. Mouse sleep is polyphasic, resulting in multiple bouts of sleep lasting 2–4 min throughout their sleep cycle. Thus, the sweep bar mounted at the bottom of the chamber moved along the bottom of the cage every 2 min during the ZT0–ZT16 to interrupt the sleep and was stationary during the ZT17–ZT24 for 8 or 9 weeks. The period of ZT0–ZT12 was around onset of the sleep cycle with high sleep need. Therefore, the SF resulted in decreasing sleep duration and bout length, and in increasing sleep state transition and waking. Mice in the SF chamber was maintained in a standard, specific pathogen-free facility at a controlled temperature (22–24 °C), with a 12 h dark/ light cycle (07:00 to 19:00 light on), with standard food and water provided ad libitum and environmental enrichments.

### Metabolic cages

Indirect calorimetry was performed using a promethion metabolic cage system (Sable Systems International, USA). Mice were placed individually in chambers for 3 days under controlled conditions at a controlled temperature with 12-h light/dark cycles. Mice had free access to food and water. Respiratory measurements were taken at 20-min intervals following an initial acclimation period. Energy expenditure was calculated using the Lusk equation: energy expenditure in Kcal/h = (3.815 + 1.232 × RER) × ${{\rm{V}}_{O_{2}}}$ in mL/min. Food intake was monitored within the metabolic chambers.

### MS analysis

For MS analysis, proteins were extracted from the hypothalamus of mice with or without SF intervention. After digestion, samples were desalted using C18 Cartridges (Empore SPE Cartridges C18 standard density, bed I.D. 7 mm, volume 3 mL, Sigma Aldrich), concentrated by vacuum centrifugation and reconstituted in 40 mL of 0.1% (v/v) formic acid. LC-MS/MS sequencing and data analysis were conducted by Jingjie PTM BioLab (Hangzhou). Co. Inc. In brief, 100 mg peptide mixture of each sample was labeled using TMT reagent according to the manufacturer’s instructions (Thermo Fisher Scientific), and the mass of the peptides was identified by a Q-Exactive mass spectrometer equipped with a Nanospray Flex source (Thermo Fisher Scientific, USA). The MS/MS data were compared against the mouse fasta from UniProt using an in-house Proteome Discoverer (V2.4, Thermo Fisher Scientific, USA). Only peptides assigned to a given protein group were considered unique.

### Western blot analysis

Western blot was performed as previously described.^68,69^ Cells and tissues were lysed in the RIPA buffer (P0013B, Beyotime) with protease inhibitors and phosphatase inhibitors (C600387, Sangon). The proteins of cell lysate were separated by SDS-PAGE and blotted onto polyvinylidene fluoride (PVDF) membranes. After blocking in 5% BSA, membranes were then incubated with the primary antibody at 4 °C overnight, followed by incubation with HRP-conjugated secondary antibody at 37 °C for 1 h. Protein bands were visualized using a chemiluminescence reagent and imaged by ChemiDoc XRS Plus luminescent image analyzer. The primary antibodies used were as follows: Rcn2 (10193-2-1201 AP, 1:1000, Proteintech), Flag (14793, 1:1000, Cell Signaling Technology), Tubulin (11224-1-AP, 1:5000, Proteintech), His (TA150088, 1:1000, Origene), p-Akt (4060, 1:1000, Cell Signaling Technology), T-Akt (4691, 1:1000, Cell Signaling Technology), Grm3 (ab283572, 1:1000, Abcam), β-Actin 1202 (81115-1-R, 1:5000, Proteintech), Khc (1:2000), Cyto1 (4272, 1:1000, Cell Signaling Technology). Silver staining was performed using fast silver stain kit (P0017S, Beyotime) according to the manufacturer’s instructions.

### scRNA-seq analysis

scRNA-seq data were obtained from the NCBI SRA database (GSE87544, GSE119960, GSE132355, GSE132730, and GSE148568).^70–73^ The scRNA-seq data were integrated using the Canonical Correlation Analysis (CCA) algorithm, resulting in a final integrated reference dataset. Cells with gene counts below 500 or mitochondrial content exceeding 10% were excluded through quality control. Subsequently, 50,087 high-quality single cells meeting these criteria were retained for downstream analysis. Dimension reduction and clustering were performed using functions from Seurat V5.0.1. The cells were grouped into 27 clusters utilizing the FindClusters function with the top 20 principal components and a resolution parameter set at 0.7. Visualization was achieved through a 2D Uniform Manifold Approximation and Projection (UMAP) algorithm using the RunUMAP and DimPlot functions. Marker genes for each cluster were identified using the FindAllMarkers function in Seurat, which compared positive markers within a given cluster against all remaining clusters.

### Screening for endopeptidases

Endoprotease screening of RCN2 protein was performed in the MEROPS database. The MEROPS website (https://www.ebi.ac.uk/merops) and database were established in 1996 to present the classification and nomenclature of proteolytic enzymes. This database contains all the world’s known peptidases and their substrate cleavage site information. In the database, each protease is assigned a unique number (Peptidase Code). In brief, the endoprotease was screened by inputting the amino acid sequence around the cleavage site of Raptin (NDGR-LDPQ) in the database for subsequent research.

### Immunofluorescence staining and histochemistry

Mice were anesthetized and 4% paraformaldehyde (PFA, w/v in PBS) solutions were infused intracardially. Subsequently, the brains, adipose tissues or stomachs were paraffin embedded. The same embedding procedure was applied to human brains. Brain, stomach, and adipose tissue slices with a thickness of 5–10 µm were cut using a microtome (Leica VT1200). After paraffin removal and antigen retrieval of the slices, sections were blocked with blocking solution at room temperature for 2 h. Then they were incubated with the primary antibody working solution (diluted with blocking solution) at 4 °C for 48 h. The primary antibodies used in this study were as follows: RCN2 (10193-2-AP, 1:200, Proteintech), NeuN (ab177487, 1:500, Abcam), Iba1 (sc-32725, 1:100, Santa Cruz Biotechnology), GFAP (3670S, 1:200, Cell Signaling Technology), KLK4 (26547-1-AP, 1:200, Proteintech), KLK1 (10815-1-AP, 1:200, Proteintech), GRM3 (ab283572, 1:200, Abcam), c-Fos (ab208942, 1:200, Abcam), AVP (20069, 1:200, Immunostar), VIP (63269, 1:200, Cell Signaling Technology), GRP (20073, 1:200, Immunostar), Anti-His (12698, 1:200, Cell Signaling Technology), POMC (ab254257, 1:200, Abcam), NPY (ab120208, 1:200, Abcam). Subsequently, the sections were incubated with the secondary antibody working solution (diluted with blocking solution; 1:500, Thermo Fisher, including Alexa Fluor 488, Alexa Fluor 555, Alexa Fluor 647, Alexa Fluor 594) at room temperature for 2 h. The slides were mounted by antifade with DAPI. Images were visualized on microscope or confocal microscope.

For multi-fluorescence staining, Tyramide signal amplification (TSA) technology was used when performing co-staining with primary antibodies of the same species origin. In brief, the first round of non-covalently bound antibodies was washed out by microwave treatment, and then a second round of incubation was performed with a secondary antibody. The cycle was repeated until all antibody incubations were completed, and the final images were obtained via microscope.

### Full-length Rcn2 and Raptin synthesis

Full-length human RCN2 was purchased from Abcam (ab105594). Full-length mouse Rcn2 and Raptin were both synthesized by Sino Biological Inc (China). Briefly, protein expression was performed as follows: 1) The target gene sequence (GCGGAGGAACTGCACTACCCGCAGGGCGAGCACCGGGCGGACTACGACCGCGAAGCGCTGCTGGGTGTCCAGGAAGACGTCGATGAGTATGTTAAACTTGGCCACGAAGAGCAGCAAAGACGATTGCAGTCGATCATAAAGAAAATTGACTCGGACTCTGATGGCTTTCTTACTGAAAATGAACTCAGTCAGTGGATTCAGATGTCTTTTAAGCATTACGCTATGCAAGAAGCCAAGCAGCAGTTTGTGGAGTATGATAAGAACAGCGACGGCGCTGTGACGTGGGATGAGTACAACATCCAGATGTACGACCGGGTGATTGACTTTGATGAGAACACTGCTCTGGATGACACAGAAGAGGGGTCGTTCAGGCAGCTTCATCTAAAGGATAAGAAGCGATTTGAAAAAGCTAACCAGGATTCAGGTCCTGGTCTGAGTCTTGAAGAGTTCATTGCGTTTGAGCACCCTGAAGAAGTTGACTATATGACGGAGTTCGTCATCCAAGAGGCTTTGGAAGAACATGACAAAAATGGCGATGGGTTTGTTAGTTTGGAAGAATTTCTTGGCGATTACAGGCGGGATCCAACTGCAAATGAAGACCCAGAATGGATACTTGTTGAAAAGGACAGATTTGTGAATGATTATGACAAAGATAATGATGGCCGGATCACCACCATCACCACCATCATCACCATTAA) was inserted into baculovirus vector followed by the generation of the recombinant baculovirus according to the manufacturer’s manual. 2) Recombinant baculovirus was amplified in cells to prepare high-titer virus stocks. 3) For protein expression, cells were infected with recombinant baculovirus following standard protocols and target protein was expressed under optimal conditions. Protein purification and analysis were performed as follows: 1) Cell culture supernatant was collected via centrifugation and loaded onto an affinity purification column. 2) Target protein was eluted from the column using elution buffer. Fractions containing the protein of interest were pooled and subjected to buffer exchange into formulation buffer. 3) Protein concentration of the final product was determined by UV or BCA assays. 4) The purity of the final product was analyzed by SDS-PAGE.

### Raptin treatment

For cell experiments, Raptin was used at 10 ng/mL for 30–60 min. Control group was treated with vehicle (PBS).

For electrophysiological experiments, Raptin was dissolved in ACSF at a concentration of 1 ng/mL and incubated with brain slices for 5–20 min. The firing rates of neurons were detected before and during Raptin incubation.

For ICV infusion of Raptin in vivo, Raptin was loaded into a sustained release pump and sustained released at a dose of 40 ng/h/g body weight at the indicated time ( ~12 days or 4 weeks). In the study of WT mice with HFD feeding, ICV infusion was initiated at 9 weeks and sustained for 12 days. In the study of SF mice or *Sim1*^*Cre*^*;Rcn2*^*flox/flox*^ mice, ICV infusion was initiated at the 5th week of SF intervention and sustained for 4 weeks. In the study of *Sim1*^*Cre*^*;Rcn2*^*flox/flox*^ mice with HFD, ICV infusion was sustained for 4 weeks.

Raptin was administered into dual tissue-specific *Grm3* knockout mice via tail vein at a dose of 1 mg/kg/day every other day for 8 weeks. Raptin was injected intraperitoneally into WT mice once at a dose of 1 mg/kg/day. Control, vehicle-treated mice were injected with PBS.

### Antibody development

To evaluate plasma full-length RCN2 and Raptin levels, we produced two antibodies in company: antibody 1 specifically recognizing the RCN2 ranging from 28–249 amino acids; antibody 2 specifically recognizing the RCN2 ranging from 250–320 amino acids. The brief methods were described as follows: 10 male Balb/c mice (2–3 months, each weighing ~16–20 g) were immunized at a dose of 50 mg/mouse with the immunogen RCN2 fragment (232–267 amino acids of RCN2, LVEKDRFVNDYDKDNDGRLDPQELLSWVVPNNQGIA; this sequence includes the common fragment and different fragment of full-length RCN2 and Raptin) or His-tagged Raptin (28–240 amino acids of RCN2, AEELHYPQGEHRADYDREALLGVQEDVDEYVKLGHEEQQRRLQSIIKKIDSDSDGFLTENELSQWIQMSFKHYAMQEAKQQFVEYDKNSDGAVTWDEYNIQMYDRVIDFDENTALDDTEEGSFRQLHLKDKKRFEKANQDSGPGLSLEEFIAFEHPEEVDYMTEFVIQEALEEHDKNGDGFVSLEEFLGDYRRDPTANEDPEWILVEKDRFVNDYDKDNDGRHHHHHHHHHH) mixed with an equal volume of adjuvant (complete/incomplete Freund’s adjuvant). The adjuvant was purchased from BD Company (263910). After being mixed, they were injected subcutaneously at multiple points in the abdomen, with the second immunization at an interval of 2 weeks and the third immunization at another interval of 3 weeks. Serum titer was then measured after three immunizations. Spleens were taken for hybridoma fusion 3 days after enhanced immunization. One week after the last immunization, 50–60 mL blood was collected from the orbital vein cluster of mice. After standing at 4 °C overnight, the upper serum was separated by centrifugation for detection. An appropriate amount of detection protein was diluted to 5 mg/mL with coating buffer, then 100 μL was added to each well of the 96-well plate and coated overnight at 4 °C. The plate was washed with washing buffer at 200 μL/well once. Then the plate was sealed with 300 μL/well sealing buffer at room temperature for 1 h. The plate was washed with washing buffer twice, and then sample (gradient dilution sample and sample diluent at 100 μL/well) and detection antibody (100 μL/well to 96-well plate) were added to incubate at room temperature for 2 h. The plate was washed and added with reaction buffer at 200 μL/well, and placed at room temperature for 12 min. Finally, 50 μL/well termination buffer was added to stop the reaction, and the detection was carried out with a microplate analyzer at 450 nm wavelength. All spleen cells of immunized mice were mixed with mouse myeloma cells at a ratio of 1:1, and hybridoma cells were obtained by electrofusion method. Antigen protein was used to coat the well, the cell supernatant was analyzed by ELISA, and clones in positive wells were selected by dilution until a hybridoma cell line that stably secreted monoclonal antibodies was obtained.

### ELISA

A 96-well microplate was initially coated with 100 μL per well of the diluted Raptin capture antibody. Freshly diluted plasma samples were then added to the wells, and the plate was incubated at room temperature for 2 h. Subsequently, the HRP-Raptin detection antibody was added, and reacted with the TMB solution, leading to color change in the system. The reaction was halted with the addition of stop solution, and the absorbance at 370 nm was measured and standardized to determine Raptin concentration.

Raptin capture antibody and Raptin detection antibody were developed by Sino Biological Company. We labeled the detection antibody with HRP using HRP Labeling Reagent Kit (Innoreagents). In brief, the detection antibody was mixed with the reaction starter at 10:1 and incubated at 37 °C for 2 h. Subsequently, a termination agent was added to remove excess HRP at room temperature for 1 h. We had determined the most effective antibody combination of HRP-Raptin detection antibody and capture antibody through the following indicators and ultimately developed two available ELISA kits to test Raptin plus full-length RCN2 (called kit1 as below) or full-length RCN2 (called kit2 as below).

To ensure the reliability of Raptin concentration, we evaluated its performance from the following indicators: 1) Intra-assay accuracy: in a test, we conducted 14 repeated detections on each of three samples according to the above method. Then coefficient of variation (CV) for each sample was calculated to evaluate intra-assay accuracy. The CV for samples detected by two ELISA kits were both < 10%. 2) Inter-assay accuracy: we tested three samples in different 96-well microplates on different days, with each sample repeated 12 times. The CV for samples detected by two ELISA kits were both < 10%, which indicated good repeatability of this ELISA. 3) Dilution linearity: dilute three known concentration samples at a ratio of 1:10, 1:20, 1:40, 1:80, 1:160, and 1:320. Then, we detected the protein concentration of each diluted sample and compared the measured value with the expected value to evaluate the dilution linearity of two kits. 4) Rate of recovery: we added 1 µL target protein (Raptin or full-length Rcn2) to 100 μL blank plasma from complete *Rcn2* knockout mice, mixed well, and then detected its concentration. The ratio of measured value to expected value after dilution was the recovery rate. The experiment was repeated three times, with an average recovery rate of 116.7% and 121.4%, respectively. 5) Sensitivity: we performed 64 independent tests on blank plasma and calculated the average value. The sensitivity is determined by adding two standard deviations to average value. The sensitivity is 0.22 pmol for kit1 and 0.46 pmol for kit2. 6) The detection range are 0.54 pmol–2.17 nmol (kit1) and 0.91 pmol–1.82 nmol (kit2).

To ensure the accuracy of ELISA kits, we added target protein (Raptin or full-length mouse Rcn2 or full-length human RCN2) to 100 µL plasma from complete *Rcn2* knockout mice at a dilution rate of 1:1, 1:20, 1:40, 1:80, 1:160, and 1:320. Subsequently, the actual added concentration and actual measured concentration were found to be close without significance. In addition, we also added Raptin or full-length mouse Rcn2 simultaneously at a ratio of 1:1 into 100 µL plasma from complete *Rcn2* knockout mice, and then measured the concentration using the two kits separately to ensure that we got the acute concentration of plasma Raptin through the value of kit1 (a pmol) minus the value of kit2 (b pmol). The final concentration of plasma Raptin equals to 0.046 × (a – b) ng/mL. The same processes were confirmed by human RCN2.

### Viral vectors

We used the following viruses:

AAV-Rcn2-Cre-EGFP, AAV-Rcn2-Cre, AAV-DIO-H2B-T2A-TVA, RV-EnvA-N2C(ΔG)-tdTomato, AAV-DIO-RVG, scAAV2/1-hSyn-Cre-WPREs, AAV-Avp-Flp-mCherry, AAV-fDIO-mCherry, AAV-fDIO-hM3D(Gq), AAV-fDIO-hM4D(Gi), AAV-EF1a-DIO-GCaMp6s-EGFP, AAV-DIO-hM3D(Gq)-WPREs/AAV-DIO-hM3D(Gq)-mCherry-WPREs, AAV-DIO-hM4D(Gi)-WPREs/AAV-DIO-hM4D(Gi)-mCherry-WPREs, AAV-DIO-WPRE-hGH-pA/AAV-DIO-mCherry-WPRE-hGH-pA, AAV-DIO-ChR2, AAV-fDIO-ChR2, AAV-Avp-Cre-mCherry, AAV-hSyn-EGFP, AAV-hSyn-Rcn2-EGFP, AAV-Nos1-Cre, AAV-Grm3-Flp-EGFP, AAV-Grm3-Cre-EGFP, AAV-hSyn-shKhc-EGFP. All viruses were purchased from BrainVTA (Wuhan, China) or Hanbio Tech (Shanghai, China), and the virus titers were greater than 5 × 10^12 ^v.g/mL.

Cre-DIO system and Flp-FRT system are both well-established ways to manipulate the neuronal activity.^74^ The AAV-Avp-Flp-mCherry or AAV-Avp-Cre-mCherry was injected into SCN to label SCN^AVP^ neurons, AAV-Rcn2-Cre-EGFP were used to mark PVN^RCN2^ neurons, and AAV-Grm3-Cre-EGFP and AAV-Grm3-Flp-EGFP were used to mark PVN^GRM3^ neurons. To construct AAV-Rcn2-Cre-EGFP, we used the genomic region of the corresponding promoter of *Rcn2*, 2.3 kb chr9:55946941-55949240 (mouse GRCm39/mm39); to construct AAV-Grm3-Cre-EGFP, we used the genomic region of the corresponding promoter of *Grm3*, chr5:9775072-9777270 (mouse GRCm39/mm39).

### Stereotaxic injections

Mice were anaesthetized with 0.5%–1.5% isoflurane (RWD Life Science Co., China) and fixed on the stereotaxic apparatus (RWD Life Science Co., China). Following the exposure and disinfection of the skin area above the skull, a small incision was made to expose the skull. Bilateral small holes were drilled using a fine burr drill over the specified stereotaxic coordinates. A microsyringe containing 50–150 nL of AAV was injected bilaterally into the target area at a rate of 50 nL/min. The coordinates, defined as dorsal–ventral (DV) from the brain surface, anterior–posterior (AP) from bregma and medial–lateral (ML) from the midline, were as follows: PVN (AP: –0.9 mm; DV: –4.7 mm; ML: ±0.25 mm) or SCN (AP: –0.58 mm; DV: –5.4 mm; ML: ±0.1 mm). After completion of the injection, which included a 10-min delay, the injection pipette was gradually withdrawn, and the scalp was sutured. After the surgery, mice were given time to recover from anesthesia on a heating mat before being placed back in their home cages.

### RNAScope ISH

To detect mRNAs of *Rcn2*, *Grm3* and *Avp*, RNAScope probes were constructed (Sunpharma Co., Ltd.). The probe sequences were listed as follows: *Rcn2*, 5′- GCCAAGUUUAACAUACUCAUCGACGUCUUC-3′；*Grm3*, 5′- ACACUGCUGUACGAACCGCCAAUGACUCCUG-3′; *Avp*, 5′- ACUCCCGUGUCCCAGCCAGCUGUACCAGCCUUA-3′. As previously described,^75^ ISH was performed with a detection kit (R0306S, Beyotime). Briefly, the slides were dehydrated in graded EtOH (50, 75, and 100%) for 3 min each and dried with cool air using an ImmEdge Pen prior to allowing the development of a hydrophobic barrier around the tissue. Samples were incubated in proteinase solution at room temperature for 10 min before washing in PBS, and then incubated with *Rcn2*, *Grm3* or *Avp* probe at 60 °C for 16 h. All remaining washing steps were performed using wash buffer in the kit, unless otherwise stated. ISH signals for *Rcn2*, *Grm3* or *Avp* were detected with Zeiss confocal microscope.

### Retrograde tracing

For retrograde monosynaptic tracing, helper viruses including AAV-DIO-RVG, AAV-Rcn2-Cre and AAV-DIO-H2B-T2A-TVA were co-injected (1:1:1 ratio in 390 nL) into PVN of WT mice. Three weeks later, the ΔG-RV that encoded tdTomato (RV-EnvA-ΔG-tdTomato, 400 nL) was injected into the PVN region. Ten days after the last injection, the mice were perfused and the brain slices were collected for detecting the tdTomato signals of SCN and immunostaining with AVP-, VIP- or GRP-specific antibodies.

### Anterograde tracing

Anterograde tracing was performed as previously described. To anterogradely trace the SCN to PVN projection, scAAV2/1-hSyn-Cre-WPRE was bilaterally delivered into the SCN of mice and Cre-dependent virus (AAV-DIO-mCherry) was bilaterally injected into the PVN. Four weeks later, the mice were perfused, and its brain was sectioned for microscopy and incubated with RNAScope probe targeting *Rcn2* mRNA.

### In vivo fiber photometry for recording GCaMp signals

To investigate functional transmission from SCN^AVP^ neurons to PVN^RCN2^ neurons, viruses including AAV-AVP-Flp-mCherry, AAV-fDIO-hM4Di/hM3Dq/control were injected into the SCN of WT mice. Meanwhile, AAV-Rcn2-Cre and AAV-DIO-GCaMp6s-EGFP were injected into the PVN. As previously described,^16,76^ after virus injection, a ceramic ferrule with an optical fiber (200 mm in diameter, N.A. 0.37) was implanted with the optical fiber tip on top of PVN. The ferrule was secured on the skull with dental cement. After optical fiber implantation, antibiotics were applied to the surgical wound.

Three weeks after optical fiber implantation, GCaMp6s signals were recorded by a fiber photometry system (Thinker Tech, Nanjing, China). Recording steps were as follows: 1) Baseline recording: perform calcium signal recording under free-moving conditions for 15 min. 2) Intraperitoneal administration: mice received an intraperitoneal injection of CNO at a dose of 2 mg/kg body weight. 3) Post-administration recording: after intraperitoneal administration, continue measuring calcium signals in freely moving mice for 45 min. 4) The excitation light of 405 nm, 470 nm, and 570 nm light sources were merged into one beam through a dichroic mirror, which is then focused by an objective lens and coupled into the same multimode fiber. The laser beam was delivered by the optical fiber to excite GCaMP6s expressed in the target brain area. The excited fluorescence was collected by the same multimode optical fiber and converted into electrical signals reflecting different neuronal activity information by two weak-light detectors at the detection end. Data were analyzed using established methods. Briefly, we fit the calcium-independent isosbestic signal to the calcium-dependent signal and calculated change in fluorescence over baseline fluorescence (ΔF/F).

### Chemogenetic manipulation in vivo

For chemogenetic manipulations of SCN^AVP^ neurons, viruses (AAV-DIO-hM4Di-WPREs or AAV-DIO-WPREs) were injected into the SCN of *Avp*-Cre mice, respectively. Mice were intraperitoneally injected with CNO at a dose of 2 mg/kg body weight. After 1 h, the plasma Raptin level was measured and fluorescence staining of brain slice was performed. The 1-h or 5-h food intake was monitored upon CNO treatment.

For chemogenetic manipulations of PVN^RCN2^ neurons, viruses (AAV-Rcn2-Cre-EGFP with AAV-DIO-hM4Di-mCherry-WPREs or AAV-DIO-mCherry-WPREs or AAV-DIO- hM3Dq-mCherry-WPREs) were injected into the PVN of mice. Mice were then intraperitoneally injected with CNO at a dose of 2 mg/kg body weight. The plasma was collected for Raptin detection after 1 h of CNO treatment.

For chemogenetic manipulations of PVN^GRM3^ neurons or stomach^GRM3^ neurons, viruses (AAV-Grm3-Cre-EGFP with AAV-DIO-WPREs or AAV-DIO-hM3Dq-WPREs) were injected into PVN or stomach of mice. Mice were then intraperitoneally injected with CNO at a dose of 2 mg/kg body weight. After 1 h, the plasma Raptin, gastric emptying, and fluorescence staining of tissue slice were assessed. The 5-h food intake was monitored upon CNO treatment.

### Electrophysiology in vitro

Cas9-EGFP[KI/+];*Pomc*-Cre mice and Cas9-EGFP[KI/+];*AgRP*-Cre mice were constructed to mark ARC^POMC^ neurons or ARC^AgRP^ neurons. *Grm3*^*flox/flox*^ or *Sim1*^*Cre*^*;Grm3*^*flox/flox*^ mice were injected with AAV-Grm3-Flp-EGFP to mark PVN^GRM3^ neurons.

As previously described,^77,78^ the above mice were anesthetized with 1% pentobarbital sodium and subjected to cardiac perfusion with ice-cold NMDG cutting solution saturated with 95% O_2_/5% CO_2_ containing 93 mM NMDG, 93 mM HCl, 2.5 mM KCl, 1.2 mM NaH_2_PO_4_, 30 mM NaHCO_3_, 25 mM D-glucose, 20 mM HEPES, 5 mM Na-ascorbate, 2 mM thiourea, 3 mM Na-pyruvate, 10 mM MgSO_4_, and 0.5 mM CaCl_2_, pH 7.35 with NMDG or HCl. The brain was subsequently removed and sliced slowly with a vibrating microtome (VT 1200S; Leica Inc.). Coronal brain slices containing ARC or PVN were recovered at 37 °C for 90 min in a chamber filled with oxygenated ACSF containing 118 mM NaCl, 25 mM NaHCO_3_, 3 mM KCl, 1.2 mM NaH_2_PO_4_, 2 mM CaCl_2_, 2 mM MgSO_4_, and 10 mM glucoses, equilibrated with 95% O_2_/5% CO_2_. One half and an hour later, the holding chamber with slices was placed at room temperature and the slices were ready for patch-clamp recordings.

Slices were transferred to a recording chamber and carbogen-saturated ACSF was perfused constantly at a flow rate of 2–3 mL/min at room temperature. Raptin was added to ACSF at a concentration of 1 ng/mL. Neurons in ARC or PVN were visualized under a fluorescent infrared-phase-contrast (IR-DlC) Axioskop 2FS upright microscope, which was equipped with a Hamamatsu C2400-07E infrared camera. Recording electrodes (3–5 MΩ) were pulled with a Flaming-Brown Micropipette Puller (Sutter Instruments, Novato, CA, USA) using thin-walled borosilicate glass capillaries. Current-clamp recordings were conducted using electrodes filled with an intracellular recording solution containing 145 mM potassium gluconate, 1.3 mM MgCl_2_, 4 mM Na_2_ATP, 0.4 mM Na_3_GTP, 5 mM EGTA, 10 mM HEPES. Patch-clamp recordings were obtained with a MultiClamp 700B amplifier (Molecular Devices), a Digidata 1440 A analog-to-digital converter (Molecular Devices), and pClamp 10.7 software (Molecular Devices).

For optogenetics and electrophysiological recording in vitro,^79^ *Avp*-Cre mice injected with AAV-DIO-ChR2 into the SCN and AAV-Rcn2-Cre-EGFP into the PVN were anesthetized with pentobarbital sodium. Then the brain slices containing SCN and PVN were ready for patch-clamp recordings as described above. Synaptic currents were evoked using a 473-nm laser (20 mW, 20 Hz, 5 s) through an optical fiber placed near the slice and connected to an intelligent optogenetic system (NEWDOON, Aurora-220). Photostimulation was triggered by TTL signals from Clampex software. Action potentials of PVN^RCN2^ neurons were recorded before and during photostimulation of SCN^AVP^ neurons.

### Optogenetics in vivo

To investigate the activation effect of SCN^AVP^ neuron’s terminals on PVN^RCN2^ neurons and Raptin release, we expressed ChR2 in SCN^AVP^ neurons via injection of AAV-DIO-ChR2 into the SCN of *Avp*-Cre mice or injection of AAV-DIO-ChR2 with AAV-Avp-Cre into the SCN of *Sim1*^*Cre*^*;Rcn2*^*flox/flox*^ mice, while AAV-Rcn2-Cre were injected into the PVN to label PVN^RCN2^ neurons. In the study of combining chemogenetic and optogenetic manipulations, AAV-fDIO-ChR2 and AAV-Avp-Flp were injected into the SCN of mice, while AAV-Rcn2-Cre with AAV-DIO-mCherry or AAV-DIO-hM4Di-mCherry were injected into the PVN.

After virus injection, as previously described,^80^ the PVN of the above mice was implanted with a ceramic ferrule carrying an optical fiber (200 mm in diameter, N.A. 0.37). The ferrule was secured on the skull with dental cement. Antibiotics were applied to the surgical wound. 21 days after optical fiber implantation, a 473-nm laser was applied for generation of light pulses to activate SCN^AVP^ neuronal terminals on PVN. The laser power was 20 mW, 20 HZ. Mice received 60 min of photostimulation. Plasma Raptin and food intake were monitored before and after photostimulation.

### Glucose and insulin tolerance tests

As described previously,^81^ for the glucose tolerance tests, mice were intraperitoneally administered with glucose at a dose of 1.5 g/kg following an overnight fast. Blood glucose levels were monitored using the ACCU-CHEK active glucometer (Roche) at 0 min, 15 min, 30 min, 60 min and 120 min. In the insulin tolerance tests, mice were intraperitoneally treated with insulin at a dosage of 0.75 U/kg after a 6-h fast. Blood glucose levels were monitored at 0 min, 15 min, 30 min, 60 min, 90 min and 120 min.

### ICV infusion

The experiment of ICV infusion was performed as described previously.^77,82^ For cannulae implantation, the mice were anesthetized with 4% isoflurane and their heads were fixed on a stereotaxic instrument (RWD). The calvaria was exposed, a 0.5 mm hole was drilled in the foredeep, and a 28-gauge cannula (brain infusion kit 2, ALZA) was implanted into the third ventricle (AP: –0.94 mm; DV: –4.8 mm; ML: 0 mm) by comparing the typical bregma-lambda distance with the one measured in the experimental animal. The cannula was fixed to the skull with cyanoacrylate and connected to a perfusion osmotic pump (ALZA) placed in the subcutaneous space of the mouse back with a tube and the skin was sutured. After the surgery, mice were given time to recover from anesthesia on a heating mat before being placed back in their home cages. For Raptin infusion, Raptin dissolved in PBS was added to the perfusion osmotic pump and the delivery rate of Raptin into the third ventricle was 40 ng/h/g body weight and the final metabolic phenotype was analyzed at the indicated time according to the experimental needs.

### Intra-PVN cannulae and drug infusion

As previously described,^82^ for cannula implantation, mice with stereotaxic injection of AAV-Ctrl or AAV-sh*Khc* were implanted with guide cannula for direct intra-PVN infusions. A 26-gauge stainless steel internal cannula that was 0.5 mm longer than the guide cannula (4.2 mm long, RWD) was used. For this purpose, animals were deeply anesthetized with 4% isoflurane and their heads were fixed in a stereotaxic frame. The skull was exposed, a single hole was drilled according to coordinates (PVN, AP: –0.9 mm; DV: –4.7 mm; ML: ±0.25 mm) and a guide cannula was implanted. Two screws were fixed to the caudal part of the skull to have an anchor point for the dental cement. Acrylic dental cement was finally used to fix the cannula and the skin was sutured.

For drug infusion, we used 0.5 μL injections containing either vehicle (0.9% NaCl) or GRM3 agonist (LY354740, 1 nM, MCE, HY-18941) dissolved in 0.9% NaCl. For this procedure, an internal cannula was lowered into the guide cannula, connected via polyten tubing to a Hamilton syringe that was placed in an infusion pump, and 0.5 μL of liquid was injected into PVN over a 2-min-period. After the injection procedure, the injectors were kept in place for an additional 1 min to allow a complete diffusion of liquid throughout the tissue.

### Pair feeding

The pair-feeding experiments were performed as previously described.^83,84^ Briefly, each mouse was housed in a separate cage and pair-fed animals received the same amount of food as ingested by the corresponding groups. Mice with ICV infusion of Raptin had free access to fed HFD, and food intake was measured and averaged daily. Spillage and grind of food in cages was carefully monitored every day. The corresponding amount of food was given to the mice with ICV infusion of PBS. Food weight was recorded at a fixed time point every day to better calculate the amount of food paired fed to the mice.

### EEG/EMG implantation and recording

The mice received a prefabricated head mount containing 2 channels of electroencephalographic (EEG) and 1 channel of electromyographic (EMG) on cervical paraspinal muscles as described previously.^85^ The mice were allowed to recover for at least 10 days before initiation of sleep recording.

For EEG/EMG implantation, after the mice were anesthetized with isoflurane, their heads were placed horizontally and fixed on a brain stereotaxic apparatus. Mouse hair was removed, the scalp was disinfected with alcohol, and then the scalp was cut open to fully expose the skull. Then, the anterior fontanelle was used as the reference point, and the left prefrontal cortex (AP: +2.1 mm; ML: +0.5 mm) and the right parietal cortex (AP: –1.7 mm; ML: +1.0 mm) were selected as the EEG electrode implantation area. After that, the skull drill was used to drill holes in the corresponding skulls in the brain area, and the EEG electrode was slowly screwed into the skull hole. At the same time, a hole was drilled in the skull above the cerebellum position of the mouse, and the implanted electrode was used as a reference electrode and a grounding electrode. The small screw was fixed on the surface of the mouse skull with dental cement. After the dental cement was hard, the silver wire was connected to the EEG adapter. The bare metal wire at the end of the EMG signal line was inserted into the neck muscle and fixed with a surgical suture, and then the neck skin was sutured. Finally, the mice were placed on a heating pad. After the mice were fully awake, they were placed back in the cage and given sufficient water and food.

For EEG/EMG recordings, the mice recovered for one week after operation, and the wound condition of the mice was closely observed during the period. If there was exudation, iodine disinfection should be given in time. The recovered mice were placed in a soundproof, well ventilated, 12-h/12-h automatic light cycle sleep recording room with constant humidity and constant temperature. After that, the mice were connected to the recording system for 2 days, and then the sleep-wake recording began. Using sleepy Score software, the sleep/wake state is divided into NREM sleep (high amplitude, 1–4 Hz delta frequency, tense EEG and EMG signals), REM sleep (high amplitude, 6–9 Hz Theta frequency, EEG and EMG signal relaxation) or wakefulness (low amplitude, fast EEG and high amplitude, variable EMG signals). EEG/EMG traces were recorded for 48 h for HFD-fed mice, *Sim1*^*Cre*^*;Rcn2*^*flox/flox*^ mice, *Sim1*^*Cre*^*;Grm3*^*flox/flox*^ mice and the corresponding control mice, and data from the second day were extracted for analysis.

### Isolation of sub-hypothalamic brain regions

To label the region of PVN, the Cas9-EGFP mice were mated with PVN-specific *Rcn2* or *Grm3* knockout mice. Mice were anesthetized with 1% pentobarbital sodium and subjected to cardiac perfusion with ice-cold NMDG cutting solution saturated with 95% O_2_/5% CO_2_ containing 93 mM NMDG, 93 mM HCl, 2.5 mM KCl, 1.2 mM NaH_2_PO_4_, 30 mM NaHCO_3_, 25 mM D-glucose, 20 mM HEPES, 5 mM Na-ascorbate, 2 mM thiourea, 3 mM Na-pyruvate, 10 mM MgSO_4_, and 0.5 mM CaCl_2_, pH 7.35 with NMDG or HCl. The brain was subsequently removed into ice-cold NMDG cutting solution and sliced slowly with a vibrating microtome (VT 1200S; Leica Inc.). The brain slices (100 μm) containing PVN were recognized by EGFP fluorescence under Leica DMi8 inverted microscope and isolated using a 26 G needle on a 1 mL syringe. Isolated brain regions were immediately put into a 1.5 mL microcentrifuge tube containing RIPA protein lysate, and the tissue was sheared through frozen tissue grinder. After that, the lysate was centrifuged at 12,000 rpm for 10 min, and the supernatant was collected and stored in a –80 °C refrigerator for subsequent research.

### trFRET measurements

24 h after transfection of SNAP-tagged GRM3 plasmids, HEK293 cells were incubated at 37 °C for 1 h with a solution of 100 nM of SNAP-Lumi4-Tb and 60 nM of SNAP-Green in Tag-Lite buffer for labeling. After being labeled, cells were washed three times with Tag-Lite, and Raptin or glutamate was added.

The trFRET measurements were performed in Greiner black 96-well plates on a PHERAstar FS microplate reader with the following setup: after excitation with a laser at 337 nm (40 flashes per well), the fluorescence was collected at 520 nm for a 50-µs reading after a 50-µs delay after excitation (window 1) or for a 400-µs reading after a 1200-µs delay (window 2). The acceptor ratio was determined by dividing the signal measured in window 1 by the signal measured in window 2 and then was plotted on logarithmic scale. The intensity level of window 2 was above noise level by at least a factor of 5 to avoid erroneous divisions. We chose this representation because, in the range of acceptor ratio measured, the log ratio is correlated linearly with the distance between Lumi4Tb and the Green acceptor used (Förster radius of pair is 4.6 nm).

### Immunoprecipitation

Protein A/G magnetic beads (HY-K0202, MCE) were incubated with anti-His antibody for 2 h at room temperature. His-Raptin was incubated with cell lysates from hypothalamic GT1-7 neurons for 2 h at room temperature. Then, we mixed the above two complexes at 4 °C overnight. After rinsing two times, immunoprecipitants were separated by SDS-PAGE, and the gel was used for MS analysis or transferred onto a PVDF membrane for further study.

### Saturation binding assay

Different doses of biotin-labeled His-Raptin were incubated with HEK293T cells, with or without GRM3 overexpression or mutant GRM3-VFT (NM_000840.3, amino acids of R30–S500) overexpression, for 30 min at room temperature. To assess nonspecific binding, 50-fold excess unconjugated His-Raptin was used. After three washes with PBS, streptavidin-HRP was added to the cells. The absorbance was measured at 450 nm, and the results were normalized to protein content.

### Molecular dynamics simulation and protein–protein docking

The AlphaFold structure of the RCN2 protein was obtained from the RCSB Protein Data Bank. Six Ca^2+^ ions were manually placed in the EF-hand domain of the protein using UCSF Chimera. Molecular dynamics (MD) simulations were performed on the Yinfo Cloud Computing Platform (YCCP) using the AmberTools 20 package and AMBER ff19SB force fields for protein. The system was solvated with a truncated 34 octahedron water box using the OPC water model and a 10 Å margin. Periodic boundary conditions (PBCs) were used, and the net charge was neutralized with Na^+^ ions. Nonbonded van der Waals interactions were calculated using Lennard-Jones 12- 6 potentials with a 10 Å cutoff, and long-range electrostatics were treated using the Particle Mesh Ewald (PME) algorithm. The SHAKE algorithm was applied to constrain hydrogen atom bonds. To remove improper atom contacts, the structure was minimized using a combination of steepest descent and conjugate gradient methods under a harmonic constraint. The system was then gradually heated to 300 K through a 20 ps NVT simulation. Two equilibrations were carried out, including a 200 ps NPT simulation with constraints on heavy atoms and a 1 ns NVT simulation without restraints. Temperature was maintained at 300 K using the Berendsen thermostat, and pressure was maintained at 1 atm using the Monte Carlo barostat. Finally, a 40 ns NVT simulation with a 2 fs time step was performed, and the last frame of the simulation was used for subsequent calculations. The crystal structure of GRM3 (PDB: 3SM9) was automatically downloaded from the RCSB Protein Data Bank (http://www.rcsb.org/). Protein–protein docking of GRM3 and Raptin was performed using the ZDOCK server. The top 10 predictions of complexes were selected for further MD simulations. All ten complexes were subjected to MD simulations following the same protocol. Initially, a 2 ns NVT simulation was conducted for each system. Afterwards, the molecular mechanics/Poisson-Boltzmann surface area (MM/PBSA) method was used to calculate the binding free energy along the trajectory. The system with the most favorable binding free energy was chosen for a 100 ns NVT simulation, and the binding free energy was recalculated based on the MD trajectory. The CPPTRAJ module was utilized for analysis of root-mean-square deviation (RMSD) and hydrogen bonds. Clustering was performed using the DBSCAN algorithm. In the simulation process of 100 ns, there are always hydrogen bonds between GRM3 and RCN2, and the number of hydrogen bonds is mainly maintained in the range of 5–8. Among them, 7 pairs of hydrogen bonds with a proportion of > 20% can be seen in the key residue hydrogen bond data.

### Stomach virus injection

As previously described,^55^ after anesthesia, the mouse abdomen was shaved and cleaned, followed by a midline incision. The stomach was exposed through this incision. Corresponding viruses were loaded into a Nanofil™ 36 G beveled needle (NF36BF-2, WPI) and Silflex™ tubing (SILFLEX-2, WPI), connected to a Nanofil™ 10 μL syringe, mounted on a Pump. Multiple 30 nL careful manipulation of the needles tip to avoid major blood vessels of stomach. After completing the infusions, the needle was kept in place for 5 s before extraction to ensure full absorption. Each animal received a total volume of 1 μL. Sterile suture was then applied to the skin. The mice were given time to recover from anesthesia on a heating mat before being placed back in their home cages.

The virus of AAV9-Nos1-Cre was injected into the gastric intermuscular layer of *Grm3*^*flox/flox*^ mice to achieve specific knockout of *Grm3* in the stomach. The virus of AAV-Grm3-Cre and AAV-DIO-hM3Dq was injected into the gastric intermuscular layer of mice to chemogenetically activate GRM3^+^ neurons in the stomach.

### Acetaminophen absorption test

The gastric emptying was assessed by acetaminophen absorption. Briefly, a vehicle composed of saline, containing 1.5% (w/v) carboxymethyl cellulose (Aladdin, 9004-32-4), 1% (w/v) acetaminophen (an absorbable marker, MedChimExpress, 103-90-2) and 2% ethanol, was used to assess the gastric emptying rate. Mice were fasted and given configured suspension via oral gavage. Tail vein blood samples were collected before (0 min) and 30 min, 60 min, and 120 min after administration. Blood samples were immediately mixed with heparin and placed on ice. Plasma was separated by centrifugation at 3000 rpm for 10 min at 4 °C, and then stored at −80 °C until analysis. Plasma acetaminophen concentrations were measured using an acetaminophen detection kit (Glenbio Ltd).

### Phenol red test

The suspensions contained phenol red (5 mg/kg body weight, Sigma Aldrich, P0290), as a non-absorbable marker, to assess the gastric emptying rate. In brief, after the mice were fasted overnight, the solution containing phenol red was intragastrically administered to the mice. After 15 min, the mice were sacrificed and quickly dissected. The cardia and pylorus were clamped with a vascular clamp and the stomach was quickly removed. The stomach content was flushed twice with cold saline, and the washout solution was then collected. The debris was removed by centrifugation at 8400× *g* for 10 min at 4 °C. After adding 1 N NaOH to the supernatant (1/10 volume of the supernatant), the concentration of phenol red was spectrophotometrically measured at 560 nm. The gastric emptying rate was calculated as follows: Gastric emptying rate (%) = ((the amount of phenol red administered (mg) – the amount of phenol red remaining in the stomach (mg))/the amount of phenol red administered (mg)) × 100%

### Phosphoproteomics

Global quantitative phosphoproteomic analysis was performed on hypothalamic GT1-7 neurons treated with Raptin or PBS. The phosphopeptides were enriched using a published protocol. Briefly, peptides were digested with a solution containing 800 mL of acetonitrile (ACN), 150 mL of 3.2 M KCl, 55 mL of 150 mM KH_2_PO_4_, and 95 mL of trifluoroacetic acid. TiO_2_ beads were then added to peptides, pelleted, and washed. The beads were suspended in 100 mL of a transfer buffer (80% ACN and 0.5% acetic acid) and transferred onto the top of a C18 StageTip (Thermo Fisher Scientific). The bound phosphopeptides were eluted twice with 30 mL of an elution buffer (40% ACN and 15% NH_4_OH; high-performance liquid chromatography grade). Subsequently, samples of the phosphopeptides were concentrated in a SpeedVac for 15 min. The eluted phosphopeptides were desalted for subsequent LC-MS/MS analysis.

### Plasmid and siRNA transfection

*Grm3* siRNA sequence is: CCATGTGAGCCCTATGAAT; *Khc* siRNA/shRNA sequence is: GGAUGAGAUUAAUGAGAAATT. For transfection of siRNA or plasmids, cells were inoculated into 12-well plates and transfected using Lipofectamine 3000 (Thermo Fisher Scientific) according to the manual. Western blot was performed to detect transfection efficiency or for functional validation.

### Live-cell imaging and analysis

For live-cell imaging, neurons were imaged in special petri dish (801001, NEST) pre-equilibrated with Hibernate A low fluorescence media (BrainBits), supplemented with 2% B27, 0.5 mM L-Glutamax, and 1% Pen/Strep. Imaging was conducted using a Zeiss 880 confocal microscope with Airyscan, utilizing a 63× oil objective (Zeiss). Neurons were maintained at 37 °C and 5% CO_2_ throughout the imaging process. Axonal mitochondrial motility was visualized by transfecting KHC-RFP and mito-BFP plasmid. Presynaptic ATP levels were measured by collecting two emission images at 450–500 nm and long pass above 525 nm along axons expressing pCMV-AT1.03 (Beyotime Biotechology). The ratio of YFP/CFP represents ATP level. Ratiometric images were generated using ImageJ (NIH).

### Mitochondrial enrichment

Mitochondrial enrichment was performed as previously described.^37^ Neurons were collected and resuspended in mitochondrial isolation buffer without Ca^2+^ or Ca^2+^-chelating reagents. The cells were then disrupted through pressure filtration, passing three times through a polycarbonate syringe filter holder equipped with three 14-μm-pore-size polycarbonate track-etch membranes. Nuclei, intact cells, and large debris were pelleted by a low-speed centrifugation at 200× *g* for 5 min. The supernatant was then transferred to fresh tubes, followed by another centrifugation at 200× *g* for 5 min to eliminate any remaining debris. The collected supernatants underwent a final centrifugation at 10,000× *g* for 10 min to pellet the mitochondrial fraction. This pellet was washed once by resuspending in mitochondrial isolation buffer and subjected to another centrifugation at 10,000× *g* to pellet mitochondria for subsequent western blot analysis.

### Retrospective series

The studies were approved by the Ethics Committee at Xiangtan First People’s Hospital and written informed consents were obtained from all participants before collecting clinical data and samples (Ethical no. 2023063010).

The main inclusion criteria were as follows: 1) aged 20–50 years, with BMI of 18–35 kg/m^2^; 2) having detailed records of sleep monitoring by polysomnography (PSG). The main exclusion criteria include: 1) aged < 18 years, BMI < 18 kg/m^2^, or postmenopausal women; 2) antecedent of major neurologic or psychiatric disorders (including alcohol or drug abuse) that affect sleep; 3) presence of a chronic disease that may interfere with the interpretation of the results that could lead to secondary obesity, such as cortisolism, hypothyroidism, hypothalamic or pituitary disorders, pancreatic disorders such as pancreatic β-cell tumors, etc.; 4) current or recent treatments for insomnia disorders or obesity; 5) working at nights, evenings, early mornings or shifts.

A total of 262 patients were included. BMI ≥ 28 kg/m^2^ was administered as a standard for obesity. The age, height and weight, and reports of PSG were collected through reviewing medical records. The previously retained blood samples of all subjects were collected.

### Cohort study

This is a prospective cohort study that explored the effects of SRT on obesity and related metabolism. 65 participants with obesity and insomnia were included in this research. The studies were approved by the Ethics Committee at Xiangtan First People’s Hospital and written informed consents were obtained from all participants before collecting clinical data and samples (Ethical no. 2023110814).

The inclusion criteria were as follows: 1) male, aged 20–40 years, with BMI of 28–35 kg/m^2^, and diagnosed as simple obesity by specialist; 2) screen positive for insomnia symptoms on the Sleep Status Index and meet DSM-5 criteria for insomnia disorders or self-reported sleep efficiency of < 85%; 3) willing to accept SRT; 4) willing to accept appetite monitoring. The exclusion criteria include: 1) receiving weight loss treatment in the past two months; 2) a history of strenuous exercise, tobacco or caffeine use, a history of alcohol consumption (> 15 g of alcohol per day (equivalent to 350 mL of beer, 150 mL of wine, or 45 mL of distilled spirits) more than twice per week), or a history of substance abuse; 3) a history of a medical condition that may interfere with the interpretation of the results that could lead to secondary obesity, such as cortisolism, hypothyroidism, hypothalamic or pituitary disorders, pancreatic disorders such as pancreatic β-cell tumors, etc; 4) diseases for which SRT may be contraindicated or render inappropriate or ineffective: additional diagnoses of sleep disorders (e.g., restless legs syndrome, obstructive sleep apnea, or episodic somnambulism) or dementia or mild cognitive impairment; epilepsy, schizophrenia, or bipolar disorder; 5) working at nights, evenings, early mornings or shifts; 6) ineligible for enrollment due to the presence of a major medical condition: previous history of myocardial infarction, coronary heart disease, angina pectoris, heart failure (class II–IV), severe arrhythmia (e.g., atrial fibrillation, need for a pacemaker). Clinical data including age, sex, weight, BMI, sleep monitoring record by smart bracelet and appetite monitoring test were collected. Plasma was collected to test Raptin level.

### Participants for genotyping and Sanger sequencing

Participants with obesity (BMI ≥ 30 kg/m^2^) were eligible for entry into the genetics study. The studies were approved by the Ethics Committee at Xiangtan First People’s Hospital and written informed consents were obtained from all participants before collecting clinical data and samples (Ethical no. 2023063010). We investigated 2000 participants and performed Sanger sequencing for *RCN2* gene variant filtering. The cDNA was reverse transcribed from RNA, which was extracted from peripheral whole-blood samples. The variant was amplified by using DNA polymerase (MIX (GREEN), TSINGKE, China), and the primers (F1: TCCTCGCGTCCCTCGGTGTC; R1: TGCAGTTGGATCCCACCTGT; F2: ACACTGCTCTGGATGATGC; R2: GTGAGAAACAAGTCCGGGT). Then, the nonsense variant of *RCN2* was screened by using primers (F3: GAACACTGCTCTGGATGATGC; R3: TGGATCCCACCTGTAATCACC). Forward and reverse sequencing reactions were performed with the Big Dye Terminator Cycle Sequencing Ready Reaction Kit (PE Applied Biosystems), and the products were analyzed on an ABI 3730XL automated sequencer (PE Applied Biosystems).

### Family collection

The Chinese family who suffered from NES was recruited from Xiangtan First People’s Hospital. The study was approved by the Ethics Committee of Xiangtan First People’s Hospital (Ethical no. 2023063010). All participants involved in this study signed informed consent documents before participating in the project. There were three affected participants in this family, and neither of the affected participants was born of consanguineous parents. The patients underwent a series of clinical evaluations, including laboratory and radiography examinations in Xiangtan First People’s Hospital, such as physical examination, blood biochemical test, and body fat analysis via a dual-energy X-ray absorptiometry densitometer. Plasma was collected to test Raptin level.

Additionally, as for energy intake measurement, participants (patients in this family, 6 healthy age- and sex- and BMI-matched obese participants for each patient) could obtain sufficient foods according to their needs from our food list (namely, cereals and legumes of rice, corn, noodles, mung bean congee, soybean milk; vegetables and fruits of broccoli, cabbage, pepper, lettuce, spinach, cucumber, tomato, apple, banana, orange; meat and eggs of chicken, beef, pork, shrimp, fish, egg, milk, cheese, shakes; oils and fats of peanut oil, butter; others of chocolate, bread, French fries, ham, juice and beverages, nuts, muffin, candy, popcorn). Energy intake was calculated by converting food weight into calorie.

### Quantification and statistical analysis

In animal experiments, animals were randomly assigned to experimental groups. The experimental animals were tested according to the semi-random principle, and the experimental and the control animals were tested in turn. In all experiments involving the comparison of different time points, the different time points were randomly tested in order. We had no specific methods to blind investigators to genotype or condition during the experiments themselves, but investigators were blinded to the genotype or condition when processing data. However, non-participants analyzed each data using consistent parameters and algorithms. We chose sample sizes that are sufficient for statistical analysis based on similar techniques used in previous publications.

All inclusion and exclusion criteria of clinical studies are described in the corresponding method above. Sample sizes of cohort study (Control and SRT group) were pre-determined by power calculations. Sample sizes of retrospective series were based on the prior publications.

Data are presented as mean ± SEM. Two-tailed Student’s *t*-test was used for comparisons between two groups, and one- or two-way ANOVA was used for comparisons among multiple groups. Comparisons between two conditions were analyzed by paired two-tailed Student’s *t*-test. For clinical data, Spearman’s correlation analysis was used for the correlation coefficient. The *P* value of the multiple correlation test was adjusted by the Bonferroni method.

For consistency in comparisons, significance in all figures is denoted as follows: *P < 0.05, **P < 0.01, ***P < 0.001, ###*P* < 0.001. Statistical analysis was performed in GraphPad Prism 8 and R version 4.0.2.

## Supplementary information

Supplementary information, Fig. S1

Supplementary information, Fig. S2

Supplementary information, Fig. S3

Supplementary information, Fig. S4

Supplementary information, Fig. S5

Supplementary information, Fig. S6

Supplementary information, Fig. S7

Supplementary information, Fig. S8

Supplementary information, Fig. S9

Supplementary information, Fig. S10

Supplementary information, Fig. S11

Supplementary information, Fig. S12

Supplementary information, Fig. S13

## Data Availability

The scRNA-seq data of the hypothalamus were obtained from the NCBI SRA database (GSE87544, GSE119960, GSE132355, GSE132730, and GSE148568). The MS proteomics data and global quantitative phosphoproteomic analysis have been deposited to the ProteomeXchange Consortium via the PRIDE partner repository with the dataset identifier PXD059980 and PXD059981.
