# Author Correction: Raptin, a sleep-induced hypothalamic hormone, suppresses appetite and obesity

**DOI:** 10.1038/s41422-025-01123-6

**Published:** 2025-05-07

**Authors:** Ling-Qi Xie, Biao Hu, Ren-Bin Lu, Ya-Lun Cheng, Xin Chen, Jie Wen, Yao Xiao, Yu-Ze An, Ning Peng, Yu Dai, Genqing Xie, Qi Guo, Hui Peng, Xiang-Hang Luo

**Affiliations:** 1 Department of Endocrinology, Endocrinology Research Center, Xiangya Hospital of Central South University, Changsha, Hunan China; 2 Department of Endocrinology, The First People’s Hospital of Xiangtan City, Xiangtan, Hunan China; 3 National Clinical Research Center for Geriatric Disorders, Xiangya Hospital, Changsha, Hunan China; 4 Key Laboratory of Aging-related Bone and Joint Diseases Prevention and Treatment, Ministry of Education, Xiangya Hospital, Central South University, Changsha, Hunan China; 5 FuRong Laboratory, Changsha, Hunan China

**Keywords:** Cell biology, Molecular biology

Correction to: *Cell Research* 10.1038/s41422-025-01078-8, published online 29 January 2025

It has come to our attention that in the version of the article initially published, the image for eWAT of PBS-treated *Rcn2*^*flox*^ mice in Fig. 3m was mistakenly reused in the image for eWAT of Raptin-treated control mice in Fig. 4r. This was due to an inadvertent repeated opening the same folder during figure organization. We have meticulously reviewed our raw data and provided the correct images for Fig. 4r. This correction does not affect the quantification of the results or the conclusions of this study. We sincerely apologize for this oversight.


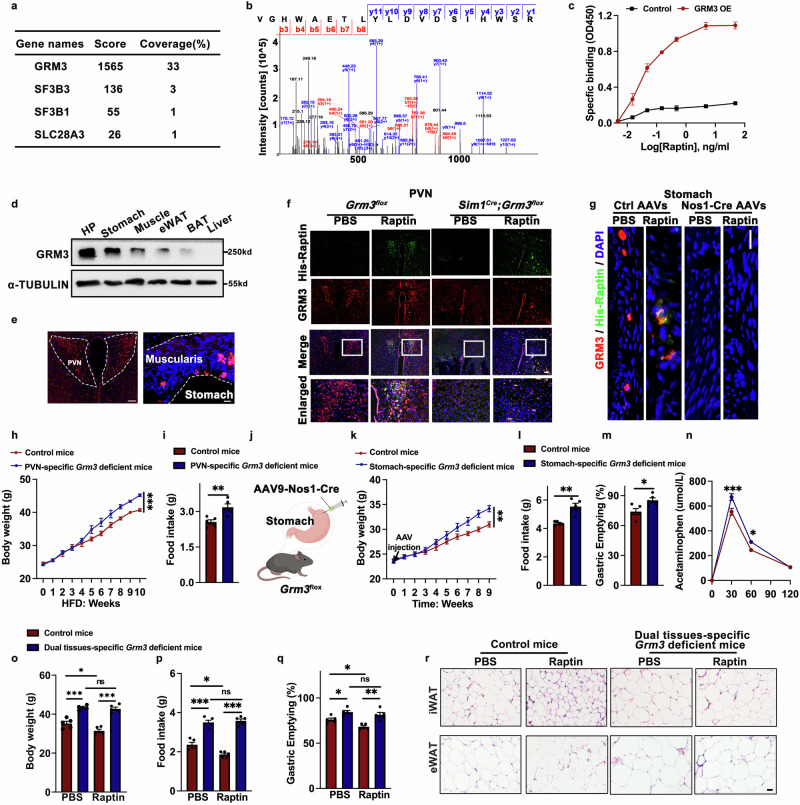


The original article has been corrected.

