# Supplementary material for: Raptin, a sleep-induced hypothalamic hormone, suppresses appetite and obesity
Source: Cell Res. 2025 Jan 29;35(3):165–85. doi: 10.1038/s41422-025-01078-8 (PMC11909135; doi:10.1038/s41422-025-01078-8)
Supplement: Supplementary file 1 — Supplementary information, Fig. S1 [file 41422_2025_1078_MOESM1_ESM.pdf]

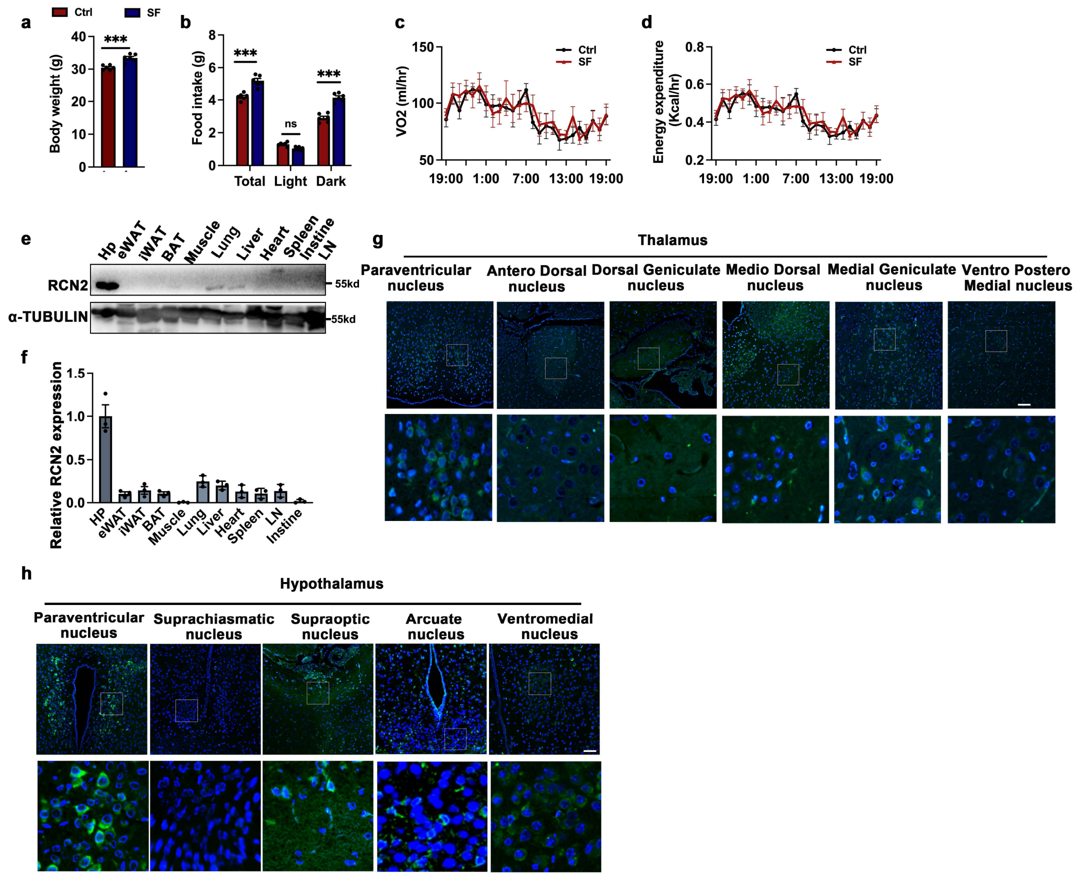

Fig. S1 RCN2 is highly expressed in PVN.

a, b Body weight (**a**) and food intake (**b**) of SF and control mice (n = 6 per group).

c, d Oxygen consumption (V_{O2}, **c**) and energy expenditure (**d**) of SF and control mice monitored over 24 hours (n = 6 per group).

e, f Representative western blot (**e**) and quantification (**f**) of RCN2 expression in different tissues.

g, h Representative images of RCN2 (green) in the different nucleus of the thalamus (**g**) and hypothalamus (**h**) (scale bar, 50 μm).

Data are shown as the mean ± SEM. ***$P < 0.001$ by two-way ANOVA (**b, c, d**) or a two-tailed, unpaired Student's *t*-test (**a**)
