# Supplementary material for: Raptin, a sleep-induced hypothalamic hormone, suppresses appetite and obesity
Source: Cell Res. 2025 Jan 29;35(3):165–85. doi: 10.1038/s41422-025-01078-8 (PMC11909135; doi:10.1038/s41422-025-01078-8)
Supplement: Supplementary file 2 — Supplementary information, Fig. S2 [file 41422_2025_1078_MOESM2_ESM.pdf]

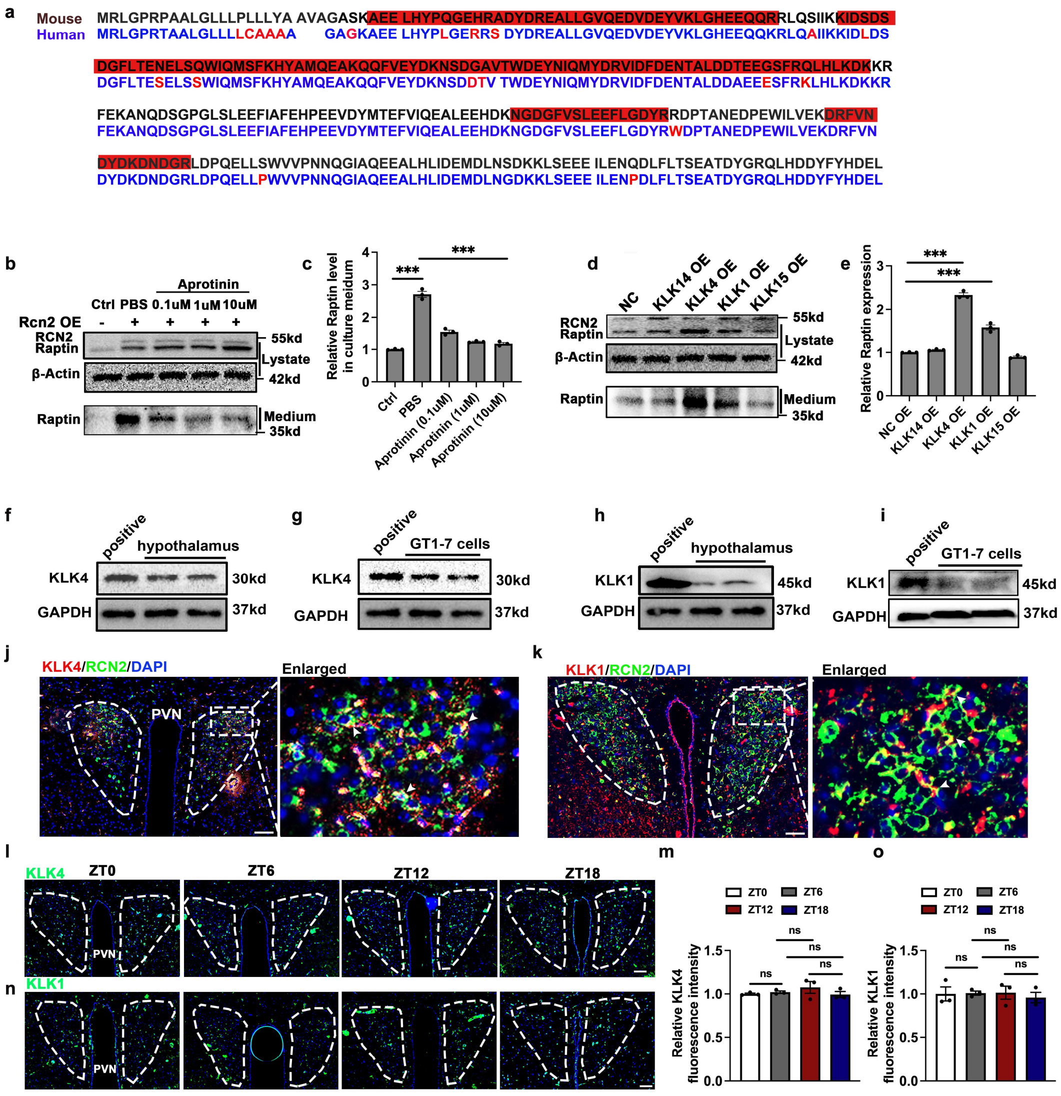

Fig. S2 Raptin is cleaved from RCN2 by KLKs.

a Alignment of RCN2 protein sequence in mouse (top) and human (bottom). The sequence of Raptin dected by MS was highlighted in red. The amino acids with red font indicate the difference between the two species.

b Representative western blot image of RCN2 expression in hypothalamic GT1-7 cell lysates and Raptin levels in the concentrated culture medium of hypothalamic GT1-7 neurons. Hypothalamic GT1-7 neurons were transfected with the Flag-*Rcn2*

plasmid with or without the treatment of indicated concentration of proprotein convertase inhibitors (Aprotinin).

c Quantification of Raptin levels in the concentrated culture medium of hypothalamic GT1-7 neurons transfected with the Flag-*Rcn2* plasmid with or without the treatment of indicated concentration of Aprotinin.

d Representative western blot image of RCN2 expression in hypothalamic GT1-7 cell lysates and Raptin levels in concentrated culture medium of hypothalamic GT1-7 neurons overexpressed with different kallikrein-related peptidases (including KLK15, 14, 1, 4)

e Quantification of Raptin levels in the concentrated culture medium of hypothalamic GT1-7 neurons overexpressed with different KLKs (including KLK15, 14, 1, 4).

f, g Representative western blot image of KLK4 expression in the hypothalamus (**f**) and GT1-7 cells (**g**).

h, i Representative western blot image of KLK1 expression in the hypothalamus (**h**) and GT1-7 cells (**i**).

j, k Representative images of co-localization staining of RCN2 (green) and KLK4 (red, **j**) or KLK1 (red, **k**) in the PVN of mice. Scale bar, 50 μm

l, m Representative images (**l**) and quantification (**m**) of KLK4 (green) expression in the PVN of mice at different time points (ZT0, ZT6, ZT12, ZT18). Scale bar, 50 μm (n = 3 per group).

n, o Representative images (**n**) and quantification (**o**) of KLK1 (green) expression in the PVN of mice at different time points (ZT0, ZT6, ZT12, ZT18). Scale bar, 50 μm (n = 3 per group).

Data are shown as the mean ± SEM. ****P* < 0.001 by two-way ANOVA (**c**) or one-way ANOVA (**e, m, o**)
