# Supplementary material for: Raptin, a sleep-induced hypothalamic hormone, suppresses appetite and obesity
Source: Cell Res. 2025 Jan 29;35(3):165–85. doi: 10.1038/s41422-025-01078-8 (PMC11909135; doi:10.1038/s41422-025-01078-8)
Supplement: Supplementary file 3 — Supplementary information, Fig. S3 [file 41422_2025_1078_MOESM3_ESM.pdf]

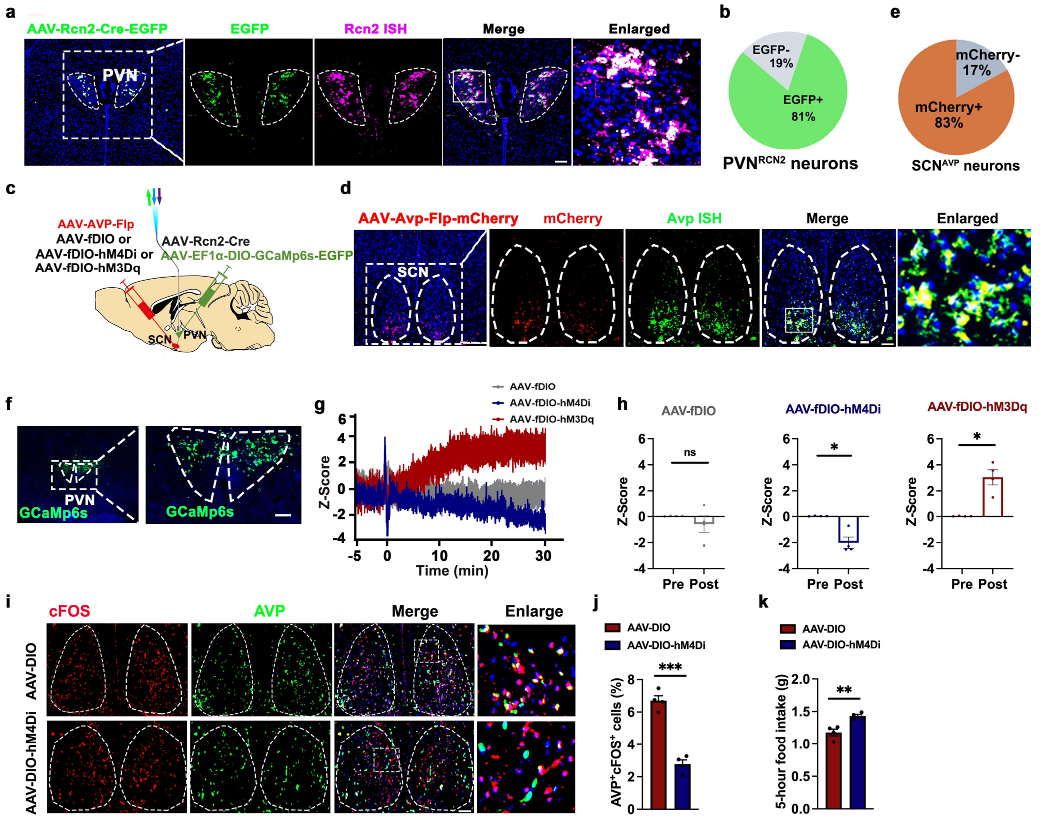


**Fig. S3 SCN$^{AVP}$ neurons control the activity of PVN$^{RCN2}$ neurons**

**a, b** Representative images (**a**) and quantification (**b**) of co-localization staining of *Rcn2* mRNA (violet) and EGFP (green) in PVN. Left: Representative low-magnification image of EGFP (green) in PVN showing the validity of constructed virus with regions of the *Rcn2* promoter (2.3 kbp) (AAV-Rcn2-Cre-EGFP) injected into the PVN of mice (scale bars, 50 μm).

**c** A schematic diagram illustrating the simultaneous chemogenetic manipulation of SCN$^{AVP}$ neuron followed by calcium imaging of PVN$^{RCN2}$ neuron. AAV-AVP-Flp along with AAV-fDIO/AAV-fDIO-hM3Dq/AAV-fDIO- hM4Di were injected into SCN. AAV-DIO-GCaMp6s-EGFP and AAV-Rcn2-Cre were injected into the PVN. The fiber was implanted into the PVN.

**d, e** Representative images (**d**) and quantification (**e**) of co-localization staining of mCherry (red) and *Avp* mRNA (green) in SCN. Left: Representative low-magnification image of mCherry (red) in SCN showing the validity of constructed virus with regions of the *Avp* promoter (AAV-Avp-Flp-mCherry) injected into the SCN of mice (scale bars, 50 μm).

**f** Representative image depicting Cre-dependent GCaMP6s expression in PVN. Scale bars, 50 μm. AAV-DIO-GCaMp6s-EGFP and AAV-Rcn2-Cre virus were injected into the PVN of mice.

**g** Line graph showing calcium levels of $PVN^{RCN2}$ neurons recorded before and after CNO-induced activation or inhibition of $SCN^{AVP}$ neurons, respectively. Mice were intraperitoneally injected with CNO at dose of 2 mg/kg body weight. (n = 4 per group).

**h** Quantification of changes in the calcium activity before and after CNO treatment in mice injected with AAV-fDIO (left), mice injected with AAV-fDIO- hM4Di (middle) and mice injected with AAV-fDIO-hM3Dq (right). (n = 4 per group).

**i, j** The representative image (**i**) and quantification (**j**) of co-localization staining c-Fos (red) and AVP (green) in SCN after CNO treatment for 60 min to induce $SCN^{AVP}$ neuron inhibition. Scale bar, 50 μm. (n = 4 per group). AAV-DIO or AAV-DIO-hM4Di were injected into SCN of *Avp*-Cre mice. Mice were intraperitoneally injected with CNO at dose of 2 mg/kg body weight.

**k** 5-hour food intake of mice after CNO-induced $SCN^{AVP}$ neuron inhibition.

AAV-DIO or AAV-DIO-hM4Di were injected into SCN of *Avp*-Cre mice. Mice were intraperitoneally injected with CNO at dose of 2 mg/kg body weight (n = 4 per group).

Data are shown as the mean ± SEM. **P* < 0.05, ***P* < 0.01, ****P* < 0.001 by a two-tailed, paired Student's *t*-test (**h**) or a two-tailed, unpaired Student's *t*-test (**j, k**)
