# Supplementary material for: Raptin, a sleep-induced hypothalamic hormone, suppresses appetite and obesity
Source: Cell Res. 2025 Jan 29;35(3):165–85. doi: 10.1038/s41422-025-01078-8 (PMC11909135; doi:10.1038/s41422-025-01078-8)
Supplement: Supplementary file 4 — Supplementary information, Fig. S4 [file 41422_2025_1078_MOESM4_ESM.pdf]

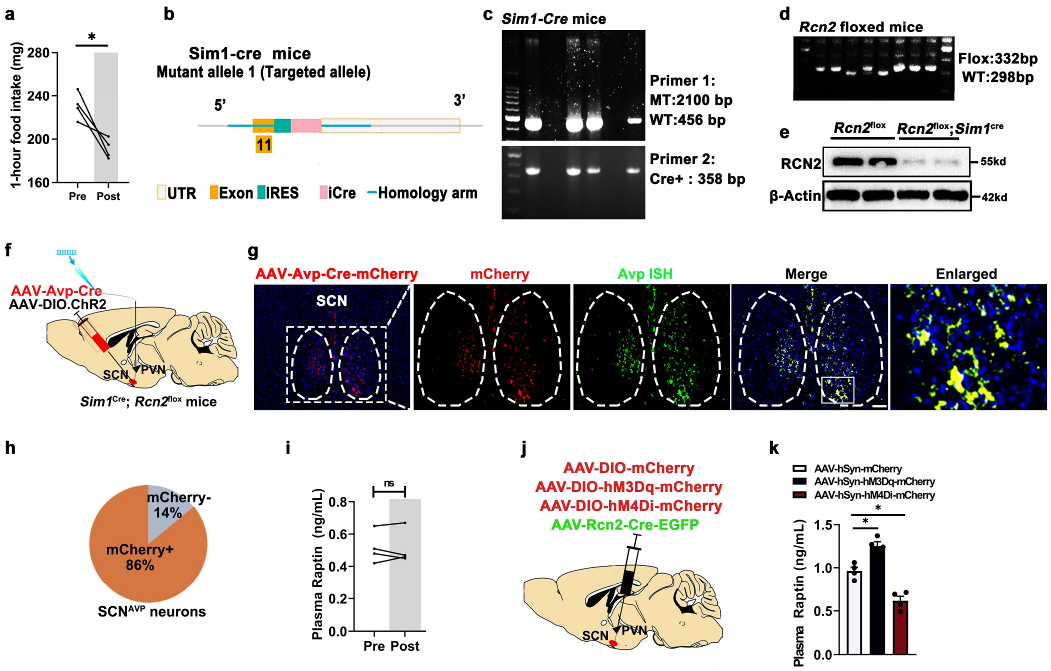

Fig. S4. PVN^{RCN2} neurons secrets Raptin.

a 1-hour food intake of mice monitored from photogenetic activation of SCN^{AVP} (n = 4 per group). AAV-DIO.ChR2 was injected into SCN and AAV-Rcn2-Cre-EGFP was injected into PVN of *Avp*-Cre mice. The fiber was implanted into the PVN concurrently to stimulate SCN^{AVP} neuronal terminal.

b Pattern diagram of the construction strategy of *Sim1^{Cre}* mice.

c, d Genotyping of *Sim1^{cre}* (**c**) and *Rcn2$^{flox/flox}$* (**d**) mice.

e Representative western blot of RCN2 in PVN of control and PVN-specific *Rcn2* knockout mice. The PVN of mice was separated by microdissection.

f A schematic diagram illustrating the simultaneous optogenetic manipulation of SCN^{AVP} neurons in PVN-specific *Rcn2* knockout mice. AAV-DIO.ChR2 and AAV-Avp-Cre-mCherry were injected into SCN of *Sim1^{cre}*; *Rcn2$^{flox/flox}$* mice. The fiber is implanted into the PVN to stimulate SCN^{AVP} neural terminal.

g, h Representative images (**g**) and quantification (**h**) of co-localization staining of mCherry (red) and *Avp* mRNA (green) in SCN. Left: Representative low-magnification image of mCherry (red) in SCN showing the validity of AAV-Avp-Cre-mCherry injected into the SCN of mice (scale bar, 50 μm).

i The plasma Raptin levels before and after optogenetic activation of SCN^{AVP} neurons (n = 4 per group).

j Schematic diagram of chemogenetic manipulations of PVN^{RCN2} neuron. AAV-Rcn2-Cre and AAV-DIO / AAV-DIO-hM4Di / AAV-DIO-hM3Dq were injected into the PVN of mice.

k Plasma Raptin levels of mice after CNO-induced inhibition or activation of PVN^{RCN2} neurons. Mice were intraperitoneally injected with CNO at dose of 2 mg/kg body weight (n = 4 per group).

Data are shown as the mean ± SEM. *$P < 0.05$ by a two-tailed, paired Student's *t*-test (**a, i**) or one-way ANOVA (**k**).
