# Supplementary material for: Raptin, a sleep-induced hypothalamic hormone, suppresses appetite and obesity
Source: Cell Res. 2025 Jan 29;35(3):165–85. doi: 10.1038/s41422-025-01078-8 (PMC11909135; doi:10.1038/s41422-025-01078-8)
Supplement: Supplementary file 5 — Supplementary information, Fig. S5 [file 41422_2025_1078_MOESM5_ESM.pdf]

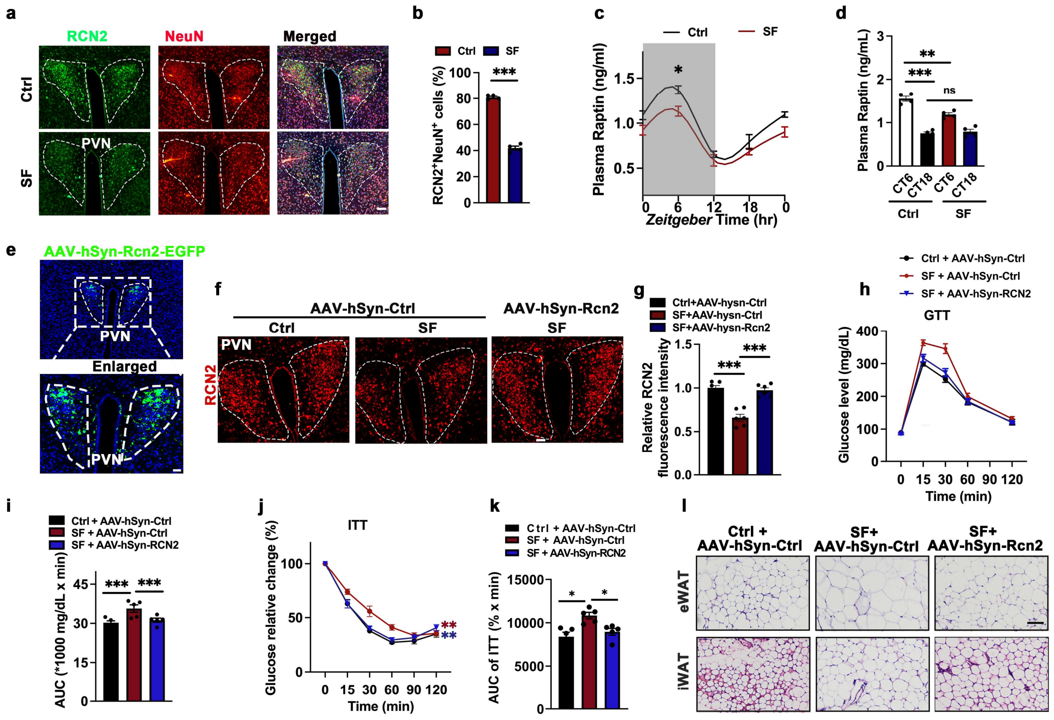

Fig. S5 Overexpression of *Rcn2* in PVN rescues SF-induced metabolic disturbance.

a, b Representative immunofluorescence images (**a**) and quantification (**b**) of RCN2 (green) and NeuN (red) in the PVN of 3-month SF and control male mice. Scale bar, 50 μm (n = 4 per group).

c Plasma Raptin levels in 3-month SF and control male mice were monitored at ZT0, ZT6, ZT12 and ZT18. (n = 4 per group).

d Plasma Raptin level of 3-month SF and control male mice at CT6 and CT18 under 12:12-dark/dark (DD) condition. (n = 4 mice per group)

e Representative images of EGFP (green) fluorescence showing the injection region of AAV-hSyn-Rcn2-EGFP in the PVN of mice (scale bars, 50 μm).

f, g Representative immunofluorescence images (**f**) and quantification (**g**) of RCN2 (red) of 3-month SF and control male mice injected with either AAV-hSyn-Rcn2 or AAV-hSyn-Ctrl (scale bar, 50um) (n = 5 per group).

h, i GTT (**h**) and AUC of GTT (**i**) in 3-month SF and control male mice injected with either AAV-hSyn-Rcn2 or AAV-hSyn-Ctrl. (n = 5 per group).

j, k ITT (**j**) and AUC of ITT (**k**) in 3-month SF and control male mice injected with either AAV-hSyn-Rcn2 or AAV-hSyn-Ctrl. (n = 5 per group).

l Representative H&E staining of eWAT and iWAT of 3-month SF and control male mice injected with either AAV-hSyn-Rcn2 or AAV-hSyn-Ctrl (scale bar, 100um) (n = 5 per group).

Data are shown as the mean ± SEM. **P* < 0.05, ***P* < 0.01, ****P* < 0.001 by a two-tailed, unpaired Student's *t*-test (**b**) or two-way ANOVA (**c, d, g-k**)
