# Supplementary material for: Raptin, a sleep-induced hypothalamic hormone, suppresses appetite and obesity
Source: Cell Res. 2025 Jan 29;35(3):165–85. doi: 10.1038/s41422-025-01078-8 (PMC11909135; doi:10.1038/s41422-025-01078-8)
Supplement: Supplementary file 6 — Supplementary information, Fig. S6 [file 41422_2025_1078_MOESM6_ESM.pdf]

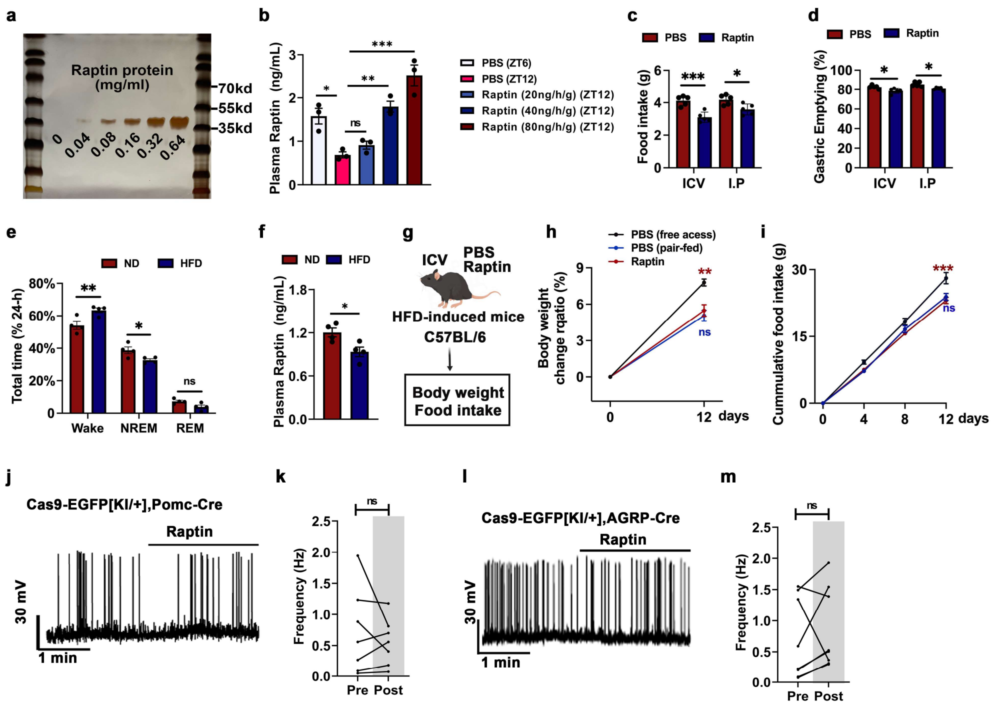

Fig. S6. Raptin treatment prevents HFD-induced obesity.

a Silver staining of purified recombinant Raptin protein, each lane containes different concentration of Raptin protein.

b Plasma Raptin levels of 3-month male mice at ZT6 and ZT12 with ICV infusion with indicated concentration of Raptin (n = 3 per group).

c The food intake of 3-month male mice with intraperitoneal injection and ICV infusion of Raptin or PBS. The intraperitoneal injection of Raptin was at dose of 1mg/kg body weight. ICV infusion of Raptin at rate of 40ng/h/g body weight (n = 5 per group).

d The gastric emptying of 3-month male mice with intraperitoneal injection and ICV infusion of Raptin or PBS. (n = 5 per group)

e The percent of wake time, NREM time and REM time of 4-month ND and HFD-fed male mice (n = 4 per group).

f Plasma Raptin levels in 4-month ND and HFD-fed male mice (n = 4 per group).

g Schematic diagram of 4-month wild-type male mice fed with a high-fat diet (HFD) with pair-feeding condition (fed PBS-treated mice with the same amount of food of Raptin-treated mice via ICV infusion).

h, i Body weight change percentage (**h**) and cumulative food intake (**i**) of wild-type mice fed with an HFD with pair-feeding condition (n = 5 per group)

j, k Representative traces (**j**) and action potential frequency (**k**) of $POMC^+$ neuron of brain slice before and after treatment of Raptin. The brain slices were from 2-month male Cas9-EGFP[KI/+], Pomc-Cre mice. Raptin dissolved in artificial cerebrospinal fluid at a concentration of 1 ng/ml were used to treat brain slices for 5-10 mins. (n = 7 per group).

l, m Representative traces (**l**) and action potential frequency (**m**) of $AGRP^+$ neuron of brain slices before and after treatment of Raptin. The brain slices were from 2-month male Cas9-EGFP[KI/+], AGRP-Cre mice. Raptin dissolved in artificial cerebrospinal fluid at a concentration of 1 ng/ml were used to treat brain slices for 5-10 mins. (n = 8 per group)

Data are shown as the mean ± SEM. $^*P < 0.05$, $^{**}P < 0.01$, $^{***}P < 0.001$ by two-way ANOVA (**b-e, h, i**), a two-tailed, unpaired Student's *t*-test (**f**) or a two-tailed, paired Student's *t*-test (**k, m**).
