# Supplementary material for: Raptin, a sleep-induced hypothalamic hormone, suppresses appetite and obesity
Source: Cell Res. 2025 Jan 29;35(3):165–85. doi: 10.1038/s41422-025-01078-8 (PMC11909135; doi:10.1038/s41422-025-01078-8)
Supplement: Supplementary file 7 — Supplementary information, Fig. S7 [file 41422_2025_1078_MOESM7_ESM.pdf]

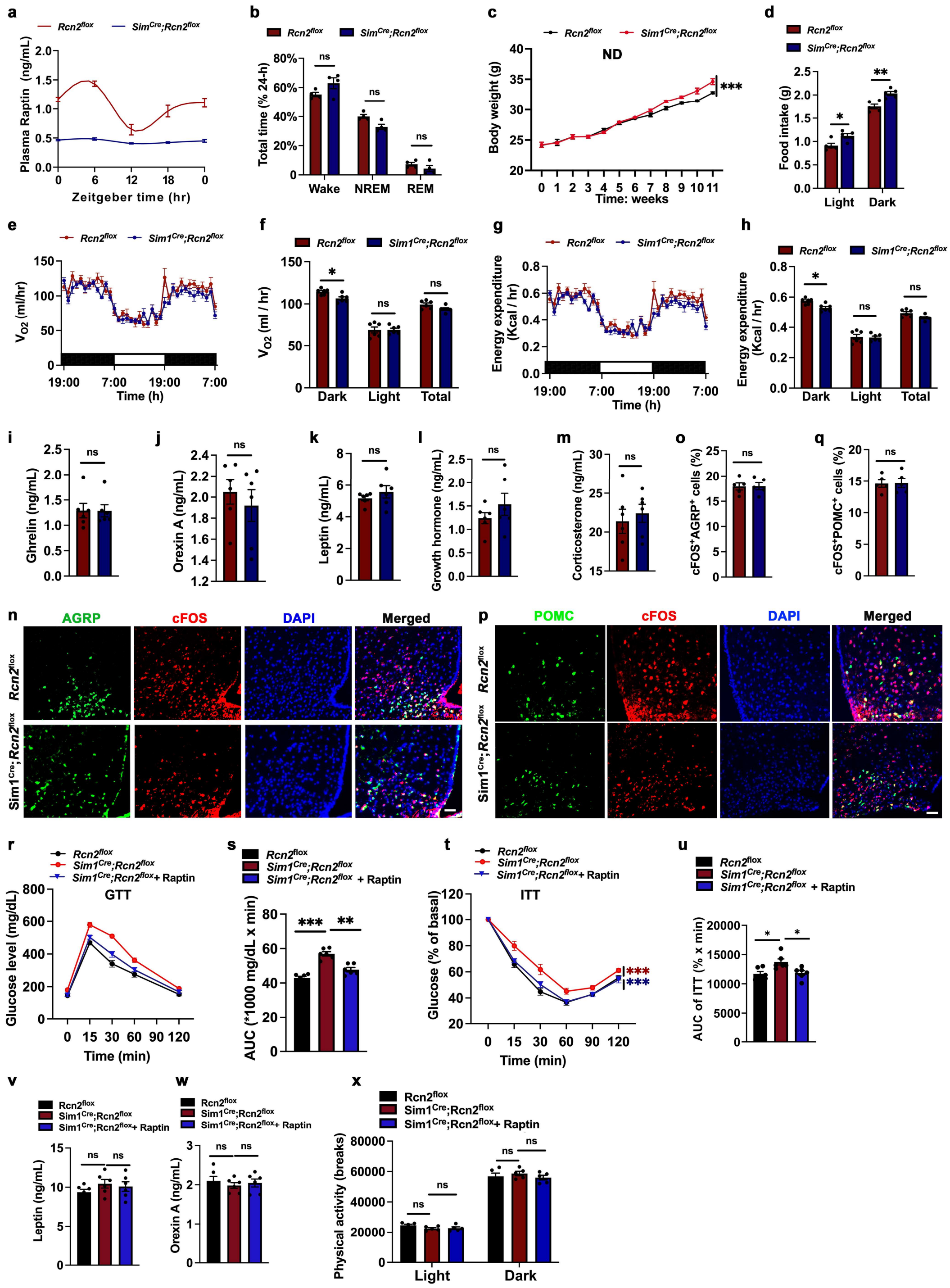

Fig. S7 PVN-specific *Rcn2* deletion causes metabolic dysfunction.

a Plasma Raptin levels in 2-month male *Rcn2*$^{flox/flox}$ and *Sim1*^{cre}; *Rcn2*$^{flox/flox}$ mice were monitored at ZT0, ZT6, ZT12 and ZT18 (n = 4 per group).

b The percent of wake time, NREM time and REM time of 5-month male *Rcn2*$^{flox/flox}$ and *Sim1*^{cre}; *Rcn2*$^{flox/flox}$ mice (n = 4 per group).

c Body weight of 5-month male *Rcn2*$^{flox/flox}$ and *Sim1*^{Cre}; *Rcn2*$^{flox/flox}$ mice fed with normal diet (ND) (n = 6 per group).

d Food intake of male *Rcn2*$^{flox/flox}$ and *Sim1*^{Cre}; *Rcn2*$^{flox/flox}$ mice.

e-h Oxygen consumption (V_{O2}, **e, f)** and energy expenditure (**g, h**) of 4-month male *Rcn2*$^{flox/flox}$ and *Sim1*^{Cre}; *Rcn2*$^{flox/flox}$ mice fed a HFD were monitored over 36 hours (n = 6 per group).

i-m The plasma levels of ghrelin (**i**), orexin A (**j**), leptin (**k**), growth hormone (**l**) and corticosterone (**m**) of 4-month male *Rcn2*$^{flox/flox}$ and *Sim1*^{Cre}; *Rcn2*$^{flox/flox}$ mice fed a HFD diet (n = 6 per group).

n, o Representative images (**n**) and quantification (**o**) of co-localization of AGRP (green) and cFos (red) staining of 4-month male *Rcn2*$^{flox/flox}$ and *Sim1*^{Cre}; *Rcn2*$^{flox/flox}$ mice (scale bar, 50um) (n = 5 per group).

p, q Representative images (**p**) and quantification (**q**) of co-localization of POMC (geeen) and cFos (red) staining of 4-month male *Rcn2*$^{flox/flox}$ and *Sim1*^{Cre}; *Rcn2*$^{flox/flox}$ mice (scale bar, 50 µm) (n = 5 per group).

r, s GTT (**r**) and AUC of GTT (**s**) of 4-month male *Rcn2*$^{flox/flox}$ and *Sim1*^{cre}, *Rcn2*$^{flox/flox}$ mice with ICV infusion of PBS or Raptin (n = 6 per group).

t, u ITT (**t**) and AUC of ITT (**u**) of 4-month male *Rcn2*$^{flox/flox}$ and *Sim1*^{cre}, *Rcn2*$^{flox/flox}$ mice with ICV infusion of PBS or Raptin (n = 6 per group). $^{***}P$ <0.001(red) when *Rcn2*$^{flox/flox}$ mice were compared to *Sim1*^{cre}, *Rcn2*$^{flox/flox}$ mice. $^{***}P$

<0.001 (blue) when *Sim1*^{cre}, *Rcn2*$^{flox/flox}$ mice were compared to *Sim1*^{cre}, *Rcn2*$^{flox/flox}$ mice treated with Raptin.

v, w The plasma levels of leptin (**v**) and orexin A (**w**) in 4-month *Rcn2*$^{flox/flox}$ and *Sim1*^{cre}; *Rcn2*$^{flox/flox}$ male mice with ICV infusion of Raptin or PBS (n = 6 per group).

x The physical activity of 4-month *Rcn2*$^{flox/flox}$ and *Sim1*^{cre}; *Rcn2*$^{flox/flox}$ male mice with ICV infusion of Raptin or PBS (n = 6 per group).

Data are shown as the mean ± SEM. $*P < 0.05$, $**P < 0.01$, $***P < 0.001$ by two-way ANOVA (**a-h, r-x**) or a two-tailed, unpaired Student's *t*-test (**i-m, o, q**).
