# Supplementary material for: Raptin, a sleep-induced hypothalamic hormone, suppresses appetite and obesity
Source: Cell Res. 2025 Jan 29;35(3):165–85. doi: 10.1038/s41422-025-01078-8 (PMC11909135; doi:10.1038/s41422-025-01078-8)
Supplement: Supplementary file 8 — Supplementary information, Fig. S8 [file 41422_2025_1078_MOESM8_ESM.pdf]

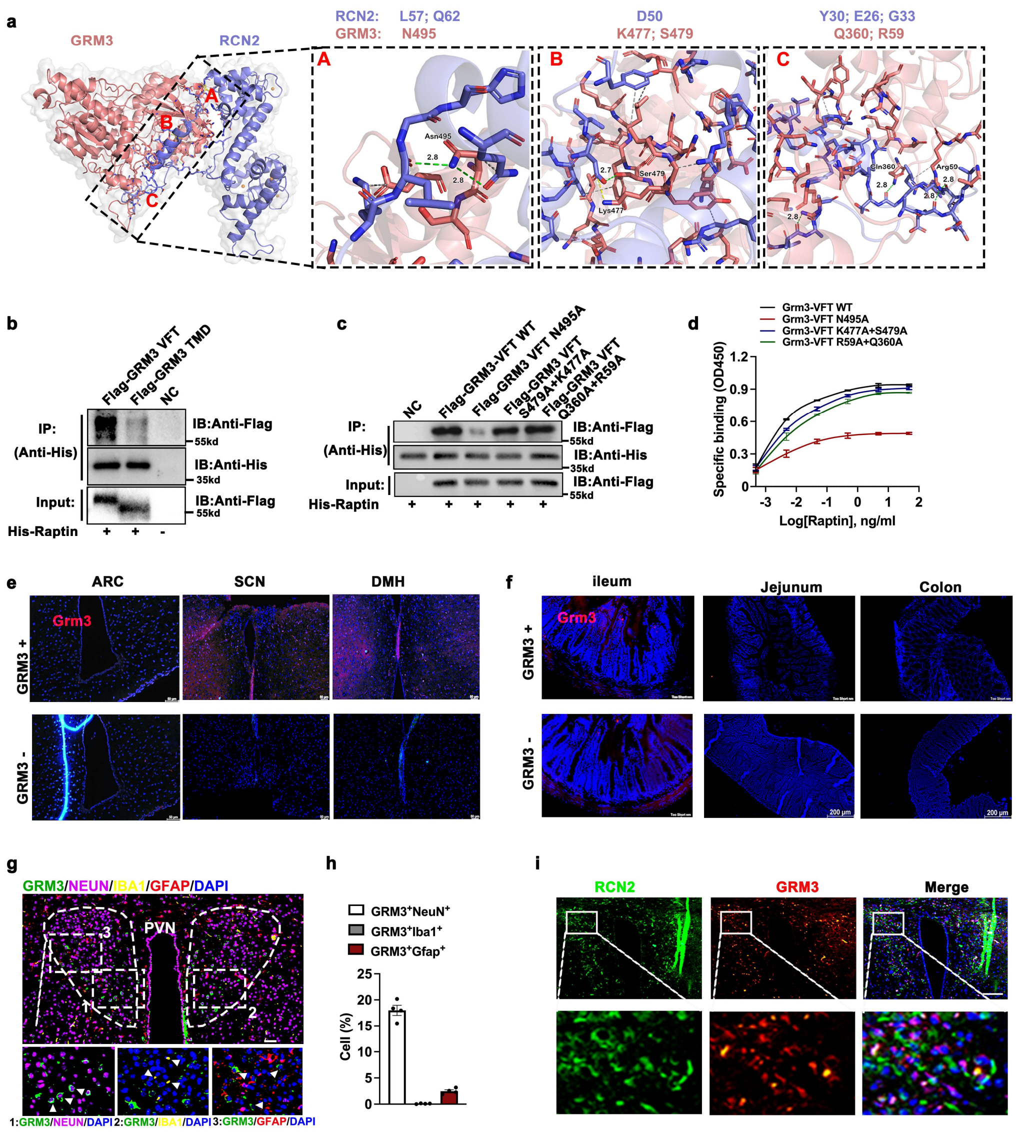

Fig. S8 GRM3 is identified as the receptor of Raptin.

a A cartoon illustrating the binding sites of human GRM3 extracellular Venus flytrap (VFT) (deep orange-red cartoon) and RCN2 (blue-gray slate cartoon) which were predicted by molecular dynamic simulation and protein-protein docking. Regions A, B and C are the key regions for the binding of GRM3 and RCN2. Asn495 (N495), Lys477(K477), Ser479(S479), Arg59(R59) and Gln360(Q360) are the key residues located on GRM3 that bind to RCN2.

b Immunoprecipitation (IP) analysis of binding between Raptin and GRM3 VFT or transmembrane domain (TMD) of GRM3 in hypothalamic GT1-7 neurons. The cell lysate of hypothalamic GT1-7 neurons with human GRM3-VFT or human GRM3-TMD overexpression was incubated with PBS or His-Raptin.

c IP analysis of binding between His-Raptin and GRM3-VFT with mutations at indicated sites in hypothalamic GT1-7 neurons (human *GRM3*-VFT N495A: the 495th asparagine of *GRM3*-VFT was mutated to alanine, *GRM3*-VFT S479A+K477A: both the 479th serine and 477th lysine of *GRM3*-VFT were mutated to alanine, *GRM3*-VFT Q360A+R59A: both the 360th glutamine and 59th arginine of *GRM3*-VFT was mutated to alanine).

d Effect of Raptin on GRM3 binding in HEK293T cells transfected either with *Grm3*-VFT plasmid or *Grm3*-VFT plasmid with mutations at indicated sites.

e Representative images of GRM3 (red) staining in brain slice section of ARC, SCN and DMH with (top) or without (bottom) incubation of GRM3 antibody (scale bar, 50 μm).

f Representative images of GRM3 (red) staining in slice section of ileum, jejunum, and colon with (top) or without (bottom) incubation of GRM3 antibody (scale bar, 200 μm).

g, h A representative image (**g**) and quantification (**h**) of GRM3 (green) expression in the neurons (NeuN, violet), microglia (IBA1, orange) and astrocytes (GFAP, red) of mouse brain slice. Scale bars, 50 μm.

i Representative images of RCN2 (green) and GRM3 (red) staining in PVN of mice (scale bar, 50 μm).

Data are shown as the mean ± SEM.
