# Supplementary material for: Raptin, a sleep-induced hypothalamic hormone, suppresses appetite and obesity
Source: Cell Res. 2025 Jan 29;35(3):165–85. doi: 10.1038/s41422-025-01078-8 (PMC11909135; doi:10.1038/s41422-025-01078-8)
Supplement: Supplementary file 9 — Supplementary information, Fig. S9 [file 41422_2025_1078_MOESM9_ESM.pdf]

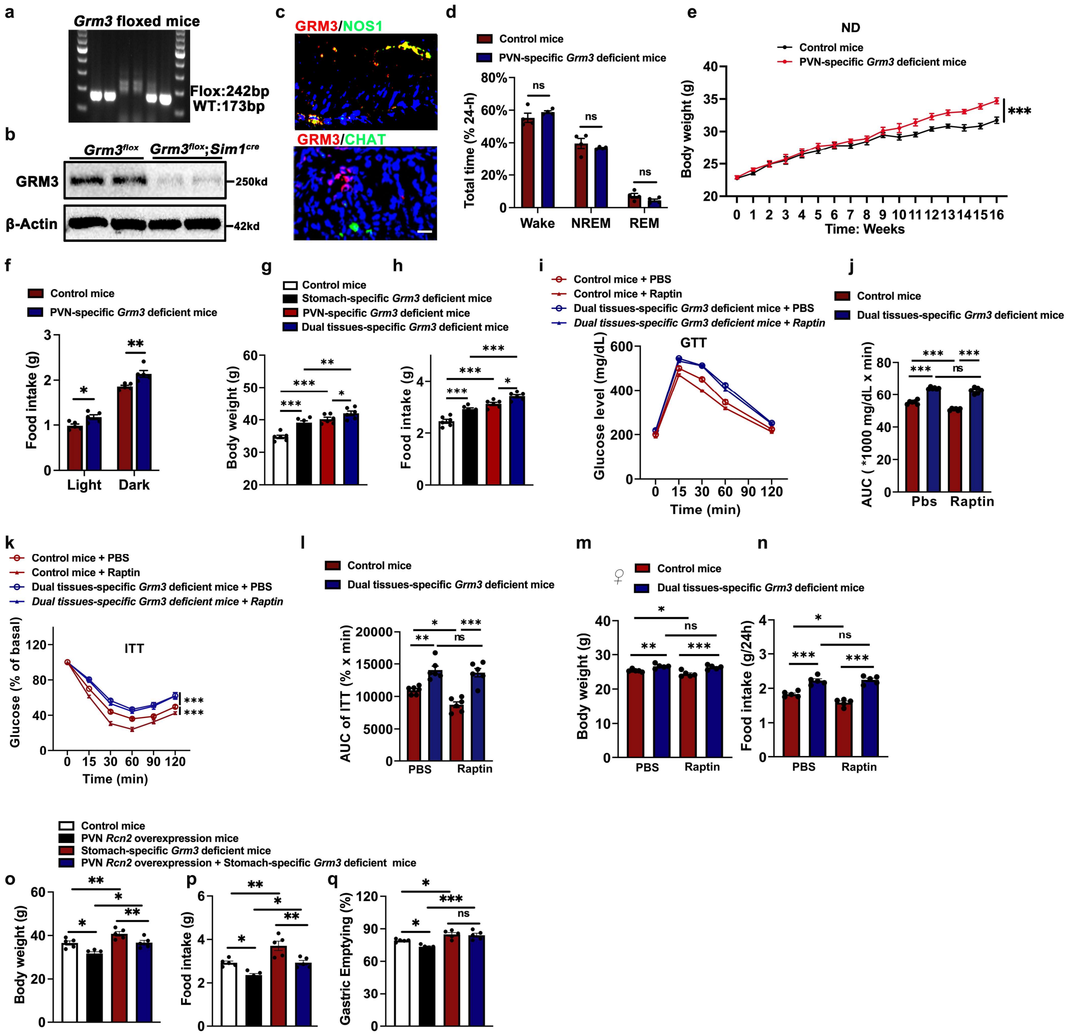

Fig. S9. Raptin alleviates obesity and metabolic dysfunction in GRM3-dependent manner.

a Genotyping of *Grm3*$^{flox/flox}$ mice.

b Representative western blot of GRM3 in PVN of *Grm3*$^{flox/flox}$ and *Sim1*^{cre}; *Grm3*$^{flox/flox}$ mice. The PVN of mice was separated by microdissection.

c Representative images of co-localization of GRM3 (red) and NOS1 (green) or CHAT (green) in the muscle layer of the stomach (scale bars, 100 μm).

d The percent of wake time, NREM time and REM time of 4-month control (*Grm3*$^{flox/flox}$ mice) and PVN specific *Grm3* deficient male mice (*Sim1*^{cre}; *Grm3*$^{flox/flox}$ mice). (n = 4 per group).

e Body weight of 6-month control (*Grm3*^{flox} mice) and PVN specific *Grm3* deficient male mice (*Sim1*^{cre}; *Grm3*$^{flox/flox}$ mice) fed a ND (n = 5 per group).

f Food intake of 4-month control (*Grm3*$^{flox/flox}$ mice) and PVN-specific *Grm3* deficient male mice (*Sim1*^{cre}; *Grm3*$^{flox/flox}$ mice) fed a HFD.

g, h The body weight (**g**) and food intake (**h**) of 4-month control male mice (*Grm3*$^{flox/flox}$ mice), stomach-specific *Grm3* deficient male mice (*Grm3*$^{flox/flox}$ mice with injection of AAV-Nos1-Cre into stomach), PVN-specific *Grm3* deficient male mice (*Sim1*^{cre}; *Grm3*$^{flox/flox}$ mice) and dual tissue-specific *Grm3* deficient male mice (*Sim1*^{cre}; *Grm3*$^{flox/flox}$ mice with injection of AAV-Nos1-Cre into stomach) (n = 6 per group).

i, j GTT (**i**) and AUC of GTT (**j**) of the control and dual tissue-specific knockout male mice treated with PBS or Raptin. Raptin was injected into mice via the tail vein at a dose of 1 mg / kg body weight every other day for 8 weeks. (n = 5 per group).

k, l ITT (**k**) and AUC of ITT (**l**) of the control and dual tissue-specific knockout male mice treated with PBS or Raptin. Raptin was injected into mice via the tail vein at a dose of 1 mg / kg body weight every other day for 8 weeks. (n = 5 per group).

m, n Body weight (**m**) and food intake (**n**) of the 4-month controls female mice and dual tissue-specific *Grm3* knockout female mice with or without treatment of Raptin. Raptin was injected into mice via the tail vein at a dose of 1 mg / kg body weight every other day for 8 weeks. (n = 5 per group).

o-q Body weight (**o**), food intake (**p**) and gastric emptying (**q**) of the 4-month male mice. Control mice: $Grm3^{flox/flox}$ mice were injected with AAV-Ctrl in PVN and stomach; PVN *Rcn2* overexpression mice: $Grm3^{flox/flox}$ mice were injected with AAV-hSyn-*Rcn2* in PVN and AAV-Ctrl in stomach; Stomach-specific *Grm3* deficient mice: $Grm3^{flox/flox}$ mice were injected with AAV-hSyn-*Ctrl* in PVN and AAV-Nos1-Cre in stomach; PVN *Rcn2* overexpression + stomach-specific *Grm3* deficient mice: $Grm3^{flox/flox}$ mice were injected with AAV-hSyn-*Rcn2* in PVN and AAV-Nos1-Cre in stomach. (n = 5 per group).

Data are shown as the mean ± SEM. $^{*}P < 0.05$, $^{**}P < 0.01$, $^{***}P < 0.001$ by two-way ANOVA (**d-q**).
