# Supplementary material for: Raptin, a sleep-induced hypothalamic hormone, suppresses appetite and obesity
Source: Cell Res. 2025 Jan 29;35(3):165–85. doi: 10.1038/s41422-025-01078-8 (PMC11909135; doi:10.1038/s41422-025-01078-8)
Supplement: Supplementary file 10 — Supplementary information, Fig. S10 [file 41422_2025_1078_MOESM10_ESM.pdf]

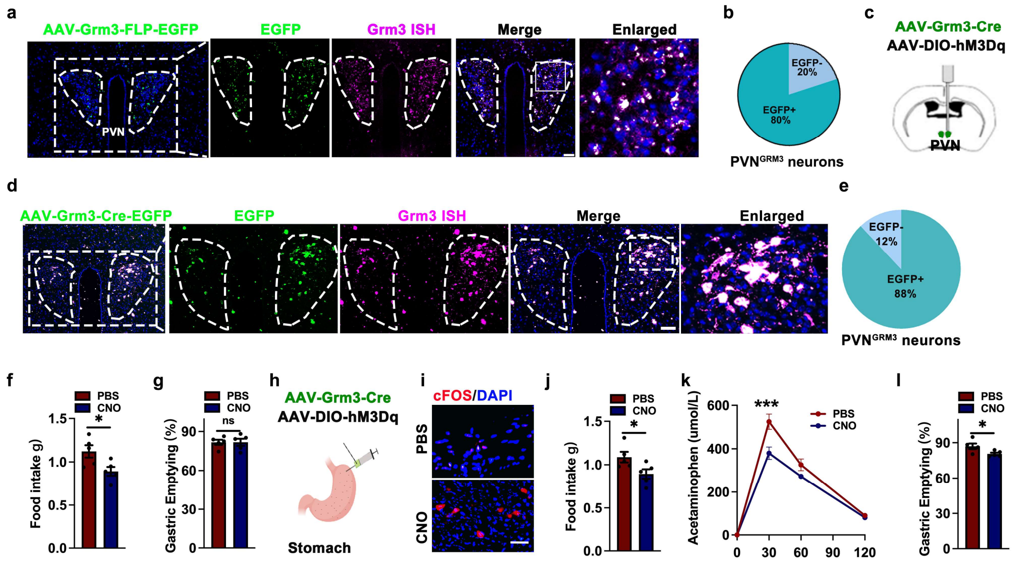

Fig. S10. Activation of GRM3^{+} neurons inhibits appetite.

a, b Representative images (**a**) and quantification (**b**) of co-localization staining of *Grm3* mRNA (violet) and EGFP (green) in PVN. Left: Representative low-magnification image of EGFP (green) in PVN showing the validity of constructed virus with regions of the *Grm3* promoter (2.3 kbp) (AAV-Grm3-Flp-EGFP) injected into the PVN of mice (scale bars, 50 μm).

c Schematic of chemogenetic manipulation of PVN^{GRM3} neuron. AAV-Grm3-Cre and AAV-DIO-hM3Dq were injected into PVN of 2-month male mice.

d, e Representative images (**d**) and quantification (**e**) of co-localization staining of *Grm3* mRNA (violet) and EGFP (green) in PVN. Left: Representative low-magnification image of EGFP (green) in PVN showing the validity of AAV-Grm3-Cre-EGFP injected into the PVN of mice (scale bars, 50 μm).

f, g 5-hour food intake (**f**) and gastric emptying (**g**) of mice after CNO-induced activation of PVN^{GRM3} neuron. CNO was intraperitoneally injected into mice at a dose of 2 mg/kg body weight.

h Schematic of chemogenetic manipulation of GRM3^{+} neuron in the stomach muscle layer of 2-month male mice. AAV-Grm3-Cre and AAV-DIO-hM3Dq were injected into stomach muscle

layer of 2-month male mice

i Representative cFos (red) staining in gastric muscle layer after CNO treatment for 60 min to activate stomach $GRM3^+$ neuron. CNO was intraperitoneally injected into mice at a dose of 2 mg/kg body weight. (Scale bars, 50 um) (n = 5 per group).

j 5-hour food intake of mice after CNO-induced activation of $GRM3^+$ neuron in stomach. (n = 5 per group).

k, l Gastric emptying of mice was measured via acetaminophen absorption test (**k**) and phenol red test (**l**) after CNO-induced activation of $GRM3^+$ neuron in stomach (n = 5 per group).

Data are shown as the mean ± SEM. $^*P < 0.05$, $^{***}P < 0.001$ by a two-tailed, unpaired Student's *t*-test (**f, g, j, l**) or two-way ANOVA (**k**).
