# Supplementary material for: Raptin, a sleep-induced hypothalamic hormone, suppresses appetite and obesity
Source: Cell Res. 2025 Jan 29;35(3):165–85. doi: 10.1038/s41422-025-01078-8 (PMC11909135; doi:10.1038/s41422-025-01078-8)
Supplement: Supplementary file 11 — Supplementary information, Fig. S11 [file 41422_2025_1078_MOESM11_ESM.pdf]

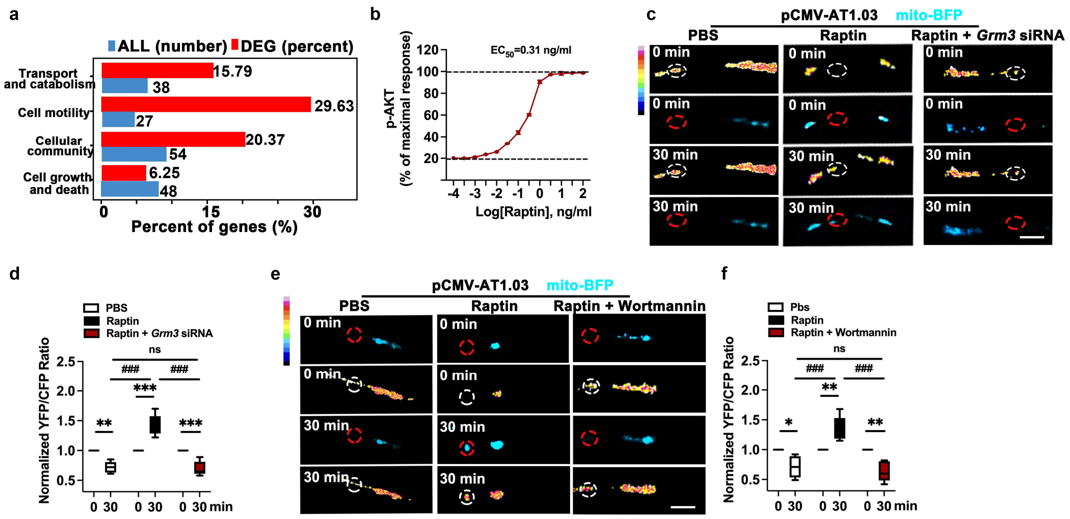

Fig. S11 Raptin-GRM3 complex controls neuronal energy supply through AKT signaling.

a Cluster of Orthologous Groups of Proteins analysis revealing the cellular processes. The changed phosphorylations were identified in the hypothalamic GT1-7 neurons treated with PBS or Raptin at 10 ng/ml for 1 hour through global quantitative phosphoproteomic analysis.

b The evaluation of Raptin treatment for p-AKT induction in GT1-7 cells.

c Representative living images of mitochondria recruiting (mito-BFP labeled, cyan) and ATP level (YFP/CFP through ratiometric images analysis) in mitochondria of control or *Grm3*-deficient primary neurons with or without Raptin treatment at 10 ng/ml. Red circles refer to the recruited mitochondria and white circles refer to ATP levels in mitochondria. Scale bar, 5 μm.

d Quantification of normalized ATP level change (YFP/CFP ratio through ratiometric images analysis) in mitochondria within 30 min in control or *Grm3*-deficient primary neurons with or without Raptin treatment.

e Representative live images of recruited mitochondria (mito-BFP labeled, cyan) and ATP level (YFP/CFP through ratiometric images analysis) in mitochondria of primary neurons with or without Raptin and wortmannin treatment at 0 min and 30 min. Red circles refer to the recruited mitochondria and white circles refer to ATP levels in mitochondria. Scale bar, 5 μm.

f Quantification of normalized ATP level change (YFP/CFP ratio through ratiometric images analysis) in mitochondria within 30 min in primary neurons with or without Raptin and wortmannin treatment .

Data are shown as the mean ± SEM. $^{*}P < 0.05$, $^{**}P < 0.01$, $^{***/\#\#\#}P < 0.001$ by two-way ANOVA (**d, f**).
