# Supplementary material for: Raptin, a sleep-induced hypothalamic hormone, suppresses appetite and obesity
Source: Cell Res. 2025 Jan 29;35(3):165–85. doi: 10.1038/s41422-025-01078-8 (PMC11909135; doi:10.1038/s41422-025-01078-8)
Supplement: Supplementary file 12 — Supplementary information, Fig. S12 [file 41422_2025_1078_MOESM12_ESM.pdf]

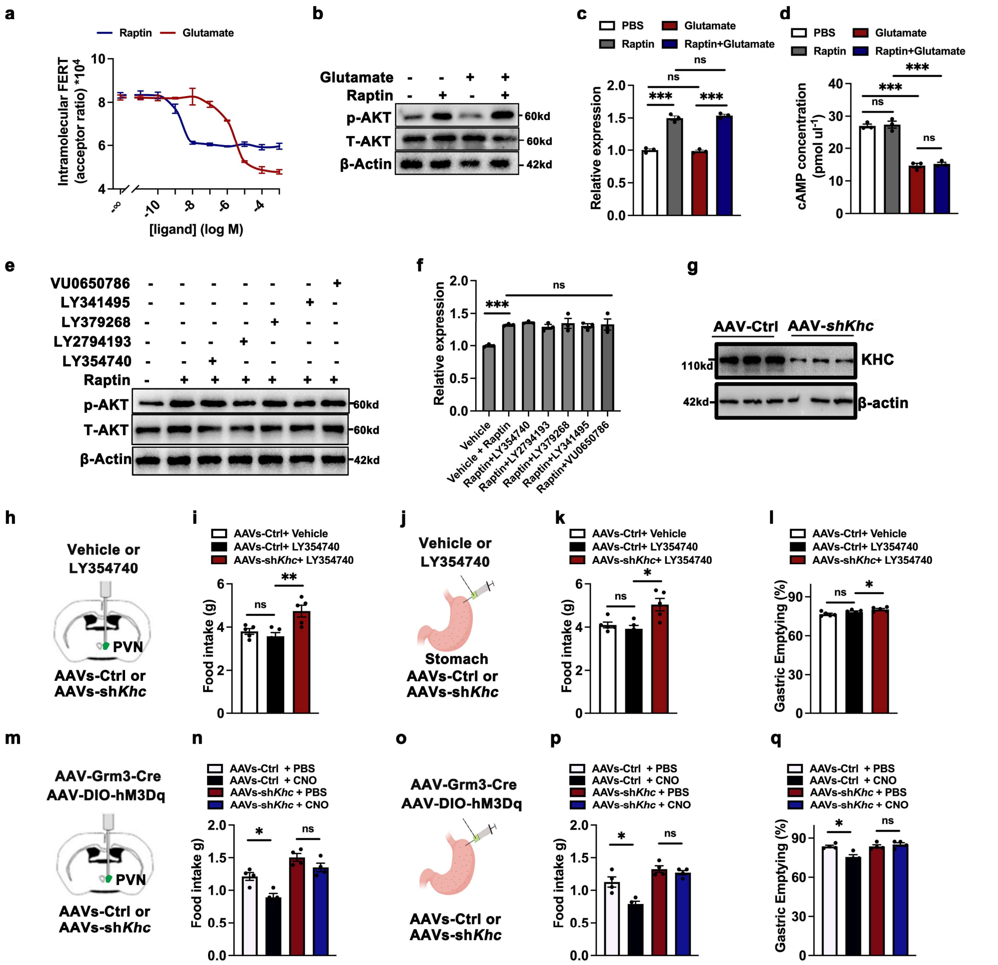

Fig. S12 Raptin shows no mutual effects with glutamate on the downstream AKT signaling.

a Time-resolved Förster resonance energy transfer (trFRET) ratios measured in GRM3-expressing HEK293T cells in the presence of Raptin or glutamate.

b, c Representative western blot (**b**) and quantification (**c**) of p-AKT signal in GT1-7 cells treated with 10 ng/ml Raptin or 50 μM glutamate for 30 mins.

d Quantification of cAMP level in GT1-7 cells treated with 10 ng/ml Raptin or 50 μM glutamate for 30 mins.

e, f Representative western blot (**e**) and quantification (**f**) of AKT signal in GT1-7 cells treated with 10 ng/ml Raptin and GRM3 agonists (LY354740, LY2794193, LY379268), inhibitors (LY341495) or allosteric agents (VU0650786).

g Representative western blot of KHC in PVN separated from mice injected with AAV-Ctrl or AAV-sh*Khc*.

h A schematic diagram illustrating injection with AAVs-Ctrl or AAVs-sh*Khc* in PVN of 2-month male mice, followed by injection of vehicle or 1 nM LY354740 into PVN.

i 24-hour food intake of the 2-month male mice injected with AAVs-Ctrl or AAVs-sh*Khc* in PVN, followed by injection of vehicle or LY354740 (n = 5 per group).

j A schematic diagram illustrating injection with AAVs-Ctrl or AAVs-sh*Khc* in stomach of 2-month male mice, followed by intraperitoneal injection of vehicle or LY354740 at dose of 15mg/kg body weight.

k, l 24-hour food intake (**k**) and gastric emptying (**l**) of 2-month male mice injected with AAVs-Ctrl or AAVs-sh*Khc* in the stomach , followed by intraperitoneal injection of vehicle or LY354740 at dose of 15mg/kg body weight. (n = 5 per group).

m A schematic diagram illustrating the chemogenetic activation of $GRM3^+$ neurons (via injection of AAV-Grm3-Cre and AAV-DIO-hM3Dq in PVN) of 2-month male mice injected with AAVs-Ctrl or AAVs-sh*Khc* in PVN.

n 5-hour food intake of 2-month male mice injected with AAVs-Ctrl or AAVs-sh*Khc* in PVN after CNO-stimulated activation of the PVN^{GRM3} neurons (n = 4 per group).

o A schematic diagram illustrating the chemogenetic manipulation in $GRM3^{+}$ neurons (via injection of AAV-Grm3-Cre and AAV-DIO-hM3Dq) of 2-month male mice injected with AAVs-Ctrl or AAVs-sh*Kh*c in the stomach.

p, q 5-hour food intake (**p**) and gastric emptying (**q**) of 2-month male mice injected with AAVs-Ctrl or AAVs-sh*Khc* in the stomach after CNO-stimulated activation of the stomach neurons (n = 4 per group).

Data are shown as the mean ± SEM. *$P < 0.05$, **$P < 0.01$, ***$P < 0.001$ by two-way ANOVA (**c, d, f, i, k, l, n, p, q**).
