# Supplementary material for: Raptin, a sleep-induced hypothalamic hormone, suppresses appetite and obesity
Source: Cell Res. 2025 Jan 29;35(3):165–85. doi: 10.1038/s41422-025-01078-8 (PMC11909135; doi:10.1038/s41422-025-01078-8)
Supplement: Supplementary file 13 — Supplementary information, Fig. S13 [file 41422_2025_1078_MOESM13_ESM.pdf]

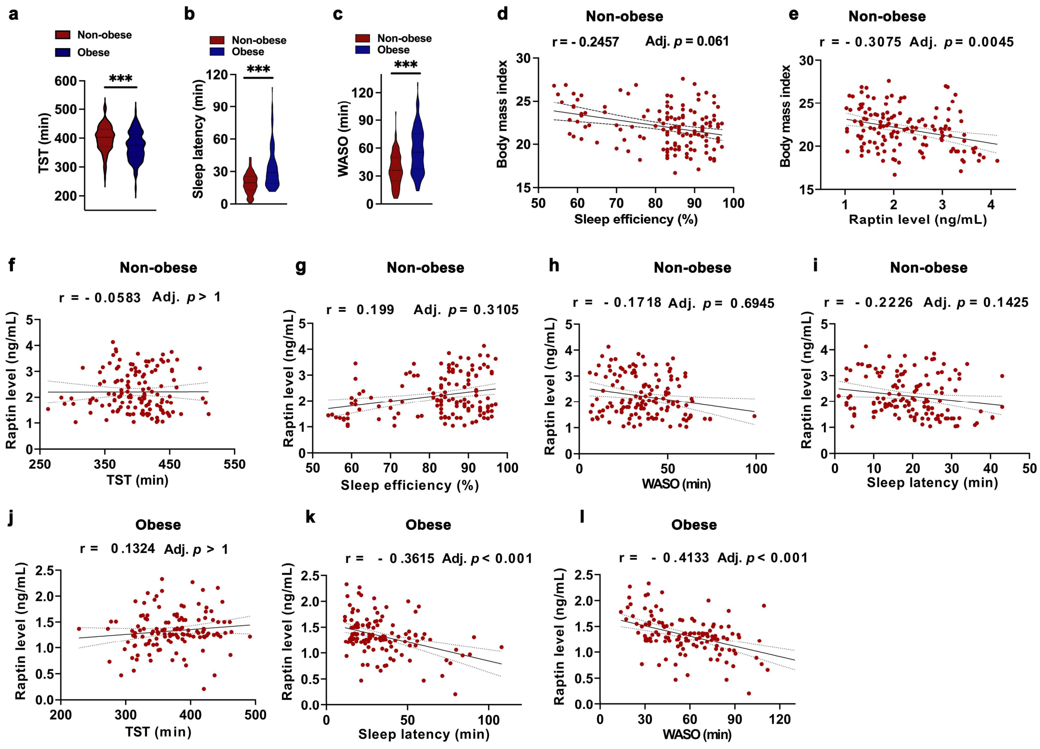

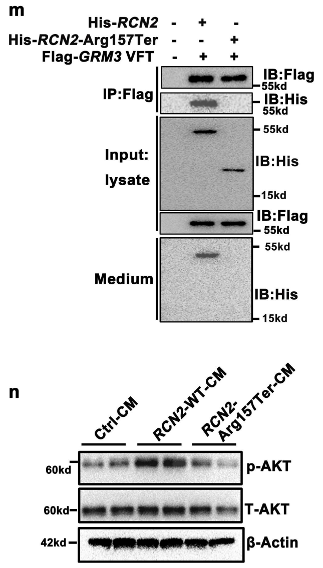

o

	Age (year)	Sex (F/M)	Weight (kg)	BMI (kg/m^2)	WHR (ratio)	Body fat (%)	TGs (mmol/L)	Raptin (ng/ml)
I1	72	M	66	22.4	0.87	25.9	1.12	1.82
II1	45	F	54	21.8	0.81	30.2	1.06	2.12
II2*	46	M	109	38.2	1.08	39.5	3.17	0.71
II3*	44	F	87	33.4	0.96	47.3	2.44	1.21
II4	45	M	68	24.2	0.85	20.1	1.26	1.96
II5	41	M	65	22.5	0.90	23.8	1.01	1.78
II6	38	F	53	21.5	0.76	23.4	0.89	2.10
III1	25	F	54	20.5	0.81	27.6	1.12	2.40
III2*	23	M	92	32.5	0.94	38.2	3.45	0.93
III3	23	M	63	21.7	0.83	28.7	1.29	2.63
III4	16	F	53	20.5	0.79	NA	1.04	2.23
III5	12	F	36	17.9	0.72	NA	0.87	2.73

Fig. S13 The clinical traits of Raptin levels, sleep quality and obesity in humans.

a-c Violin plots of total sleep time (TST, **a**), sleep latency (SL, **b**) and wakefulness after sleep onset (WASO, **c**) in obese (n = 127) and non-obese participants (n = 135).

d Spearman's correlation between sleep efficiency (SE) with BMI in non-obese participants (n = 135).

e-i Spearman's correlation between plasma Raptin levels with BMI (**e**), TST (**f**), SE (**g**), WASO (**h**) and SL (**i**) in non-obese participants (n = 135).

j-l Spearman's correlation between plasma Raptin level with TST (**j**), SL (**k**) and WASO (**l**) in participants with obesity (n=127).

m IP analysis of binding between human GRM3 and RCN2 and representative western blot images of cell lysate and medium from HEK293T cells. HEK293T cells were transfected with *GRM3*-VFT plasmid, *RCN2*-WT plasmid, and *RCN2*-Arg157Ter plasmid.

n Representative western blot images of AKT signaling in GT1-7 cell lysates. GT1-7 cell were treated with conditioned medium (CM) collected from hypothalamic cells transfected with human control, *RCN2*-WT or *RCN2*-Arg157Ter plasmid.

o Clinical characteristics of these family members, including basic information (age, sex), metabolic traits (BMI, WHR, whole body fat), blood biochemical indexes (triglycerides: TGs) and Raptin levels.

NA is not available. Data are shown as the mean ± SEM or β estimate ± 95%CI. ***$P < 0.001$ by Mann-Whitney U test (**a, b, c**) and Spearman's correlation analysis (**d-l**). The p-value of Spearman's correlation analysis were corrected by Bonferroni method.
